# Human-specific gene CT47 blocks PRMT5 degradation to lead to meiosis arrest

**DOI:** 10.1038/s41420-022-01139-6

**Published:** 2022-08-02

**Authors:** Chao Li, Yuming Feng, Zhenxin Fu, Junjie Deng, Yue Gu, Hanben Wang, Xin Wu, Zhengyun Huang, Yichen Zhu, Zhiwei Liu, Moli Huang, Tao Wang, Shijun Hu, Bing Yao, Yizhun Zeng, Chengji J. Zhou, Steve D. M. Brown, Yi Liu, Antonio Vidal-Puig, Yingying Dong, Ying Xu

**Affiliations:** 1 Cambridge-Su Genomic Resource Center, Jiangsu Key Laboratory of Neuropsychiatric Diseases, Medical School of Soochow University, Suzhou, Jiangsu 215123 China; 2 Department of Reproductive Medical Center, Jinling Hospital, Medical School of Nanjing University, Nanjing, Jiangsu 210002 China; 3 State Key Laboratory of Reproductive Medicine (SKLRM), Nanjing Medical University, Nanjing, Jiangsu 210029 China; 4 Department of Cardiovascular Surgery of the First Affiliated Hospital & Institute for Cardiovascular Science, Collaborative Innovation Center of Hematology, State Key Laboratory of Radiation Medicine and Protection, Medical College, Soochow University, Suzhou, 215000 China; 5 Department of Biochemistry and Molecular Medicine, University of California at Davis, School of Medicine, Sacramento, CA USA; 6 Medical Research Council (Mammalian Genetics Unit and Mary Lyon Centre), Harwell, UK; 7 Department of Physiology, University of Texas Southwestern Medical Center, Dallas, TX 75390 USA; 8 University of Cambridge Metabolic Research Laboratories, Institute of Metabolic Science, MDU MRC Cambridge, UK

**Keywords:** Development, Cell biology

## Abstract

Exploring the functions of human-specific genes (HSGs) is challenging due to the lack of a tractable genetic model system. Testosterone is essential for maintaining human spermatogenesis and fertility, but the underlying mechanism is unclear. Here, we identified Cancer/Testis Antigen gene family 47 (CT47) as an essential regulator of human-specific spermatogenesis by stabilizing arginine methyltransferase 5 (PRMT5). A humanized mouse model revealed that CT47 functions to arrest spermatogenesis by interacting with and regulating CT47/PRMT5 accumulation in the nucleus during the leptotene/zygotene-to-pachytene transition of meiosis. We demonstrate that testosterone induces nuclear depletion of CT47/PRMT5 and rescues leptotene-arrested spermatocyte progression in humanized testes. Loss of CT47 in human embryonic stem cells (hESCs) by CRISPR/Cas9 led to an increase in haploid cells but blocked the testosterone-induced increase in haploid cells when hESCs were differentiated into haploid spermatogenic cells. Moreover, CT47 levels were decreased in nonobstructive azoospermia. Together, these results established CT47 as a crucial regulator of human spermatogenesis by preventing meiosis initiation before the testosterone surge.

## Introduction

Understanding the function of human-specific genes (HSGs) is critically important for human biology, as they are most likely to transform our understanding of genetic and genome variation in human health [1, 2]. Relative to other organs, the testis expresses an unusually high number of species-specific transcripts [3–6]. In addition, although many mutant mouse models with reproductive phenotypes are available, our understanding of the genetic basis of human idiopathic infertility is minimal, resulting in very few therapeutic options in the clinic [7], which has led to the hypothesis that these HSGs might have participated in spermatogenesis and potential associations with human diseases. The principal obstacle has been that the physiological role of HSGs remains poorly understood due to the difficulties in studying HSGs.

Testosterone, essential for maintaining spermatogenesis and male fertility [8], binds the androgen receptor (AR) in Sertoli cells to initiate the functional response required to support spermatogenesis. There are some differences in spermatogenesis and its hormonal control between rodents and humans. Most notably, the initiation of meiosis in human spermatogenesis is evoked by the hypothalamic-pituitary-gonadal axis with a surge of testosterone at puberty after a long “prespermatogenesis” stage [9, 10]. In men, testosterone appears to be more involved in spermatogenesis than in rodents [11–14]. AR mutations are often found in patients with disorders of male reproductive development and male infertility [15]; however, the underlying mechanism of testosterone action in human spermatogenesis remains unclear.

Species-specific proteins have been proposed primarily to modulate existing signaling networks in a cell type/tissue-specific manner rather than creating new signaling hubs [16–18]. Therefore, the identification of interaction partners of species-specific proteins with an existing conserved pathway can potentially illuminate the biological functions of these novel proteins [19]. In this study, we focused on human-specific cancer/testis antigen gene family 47 (CT47), which is typically expressed in human testis but is aberrantly activated in lung cancer and esophageal cancer [20, 21]. We used co-immunoprecipitation to identify CT47, which associates with a known protein PRMT5, to study its potential function. We combined the generation of humanized bacterial artificial chromosome (BAC) transgenic mice and a CRISPR-based gene-editing method in human embryonic stem cells (hESCs) to determine the function of CT47. Our results demonstrate that CT47 interacts with and stabilizes PRMT5, acting as a gatekeeper that controls meiosis. Furthermore, CT47/PRMT5 can respond to testosterone to initiate meiosis. Notably, low expression of CT47 has been observed in human idiopathic infertility diseases, compatible with testosterone-induced initiation of meiosis (i.e., low CT47 in idiopathic infertility decreases spermiogenesis responsiveness to testosterone at puberty). Similarly, in rodents, without enough testosterone at postnatal day (P)14, a high level of human CT47 arrests mouse spermatogenesis, supporting the functional relevance of CT47 in male fertility. We thus propose that CT47/PRMT5 is synchronized by testosterone, a specific gene function in human male reproduction, which is now recapitulated in mice.

## Results

### CT47 binds and stabilizes PRMT5

**Fig. 1:**
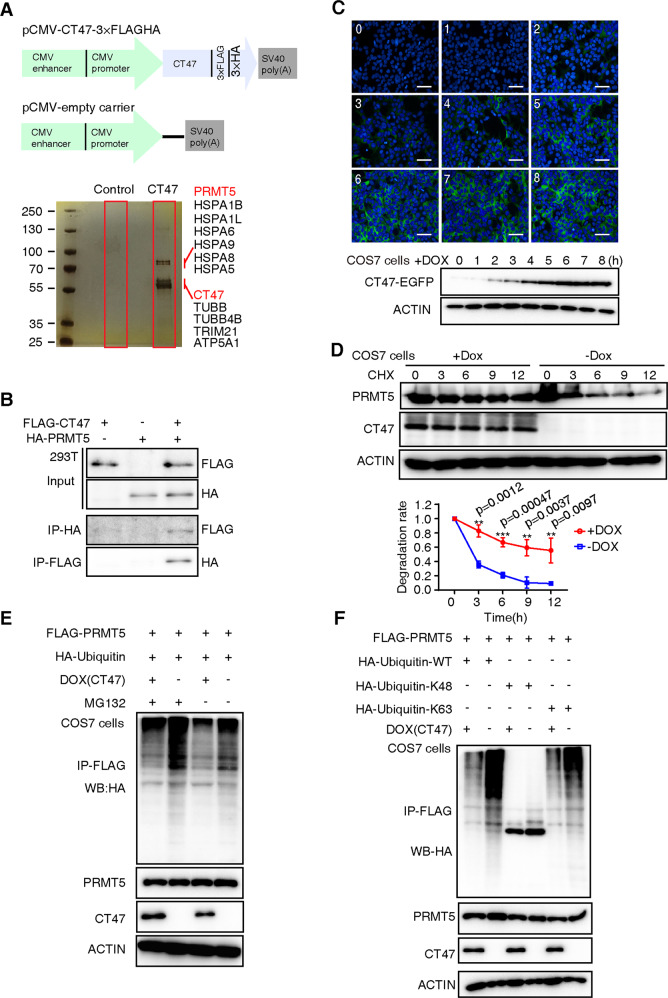
CT47 interacts with PRMT5 and inhibits its degradation. **A** Identification of CT47-interacting proteins using immunoprecipitation and mass spectrometry. Upper lane: constructs. Bottom: silver-stained polyacrylamide gels showing proteins copurified with tagged CT47 (CT47-3×FLAG.HA). The top 12 interaction candidate proteins are listed with their molecular weights. **B** The interaction between FLAG-CT47 and HA-PRMT5 was confirmed by IP assays in HEK293T cells. Reciprocal IPs were performed with anti-FLAG or anti-HA antibodies. Subsequent detection of PRMT5 or CT47 was performed using anti-HA or anti-FLAG antibodies. **C** Monitoring of time-dependent CT47 expression using a doxycycline-inducible expression system for CT47 in COS7 cells. 0–8 means doxycycline treatment time (hour). Upper: Immunofluorescence, bottom: western blot. **D** CT47 increases PRMT5 stability. HA-PRMT5 was transfected into COS7 cells. DOX (2 μg/ml) was added to induce CT47 expression. CHX or dimethyl sulfoxide (DMSO) was added after 24 h, followed by harvesting of cells at the indicated time points. Subsequent detection of PRMT5 using anti-PRMT5 antibodies. The PRMT5 protein level was quantified at each time point using ImageJ. The results shown are representative of three independent experiments. Student’s *t* test: *p < 0.05, **p < 0.01, ***p < 0.001. **E** In vitro ubiquitination assay of PRMT5. FLAG-PRMT5 was expressed in COS7 cells in which doxycycline was used to induce CT47 expression. Twenty-four hours after transfection, the cells were treated with either 20 mM MG132 or DMSO (solvent) for 6 h. The ubiquitinated PRMT5 was purified with an anti-FLAG antibody and detected with antibodies against HA. The results shown are representative of three independent experiments. **F** FLAG-PRMT5 was co-expressed with HA-Ubiquitin-WT or K48- and K63-specific ubiquitin. The results shown are representative of three independent experiments.

*CT47* consists of an array of 12 units that are identical for amino acidic sequence and tandemly arranged on human Xq24 (Fig. S1A), which specifically evolved multiple copies in the human genome after human-chimpanzee divergence (Fig. S1B). *CT47* makes more copies in humans, such as CT47A3-A12, compared to 226 species from publicly available genomes, and the CT47 protein shares no known signature motifs in the public domain databases. To identify its potential partners in a conserved pathway, we expressed and purified C-terminal 3× FLAG-HA-tagged CT47 in HEK293T cells by FLAG-immunoprecipitation followed by HA-immunoprecipitation. The immunoprecipitate was analyzed by mass spectrometry (MS) (Fig. 1A). After removing background proteins found in the control, most of the top-ranking candidate CT47-interacting proteins were from the heat shock protein family (HSPA8, HSP1B, HSP9A, HSPA5, HSPA1L, HSPA6), tubulin constituent of microtubules (TUBB, TUBB4B), a mitochondrion-related protein (ATP5A1), a ubiquitin-protein ligase complex-related protein (TRIM21), and PRMT5 (Fig. 1A). In this study, we focused on the relationship between CT47 and PRMT5 because PRMT5 is known for its ability to catalyze the methylation of specific arginine residues in a wide variety of cellular proteins to regulate transcription [22–24]. To validate their binding by MS, we tested their associations in cotransfected HEK293T cells. Reciprocal immunoprecipitation assays showed that CT47 and PRMT5 efficiently pulled down the other (Fig. 1B). These data provide evidence for the validity of the immunoprecipitation (IP)-MS results and confirm the association of the selected candidate protein PRMT5 with CT47.

We hypothesized that the CT47 protein might regulate PRMT5 stability to exert its function. We thus generated a stably transformed cell line (COS7 cells) carrying a doxycycline (DOX)-inducible *CT47* fusion variant with a green fluorescent protein (GFP) at its C-terminus (Fig. 1C). PRMT5 stability was examined in cells with/without DOX after the addition of the protein synthesis inhibitor cycloheximide (CHX). Strikingly, PRMT5 had a half-life of ~12 h in the presence of *CT47*, which was reduced to only ~2.8 h in the absence of *CT47* expression (Fig. 1D), suggesting that CT47 protects PRMT5 from degradation.

To confirm that PRMT5 is stabilized by CT47, we co-expressed FLAG-PRMT5 and HA-ubiquitin in COS7 cells in which DOX induced CT47 expression. As shown in Fig. 1E, PRMT5 was ubiquitinated, and the induction of CT47 expression led to a marked reduction in PRMT5 ubiquitination. Moreover, the proteasome inhibitor MG132 increased the levels of ubiquitinated PRMT5. These results indicate that CT47 stabilizes PRMT5 by blocking its polyubiquitination and proteasome-mediated degradation.

To determine the linkage of the ubiquitin chain on PRMT5, we co-expressed HA-ubiquitin-K48 or HA-ubiquitin-K63 (with only K48 or K63 present and other lysines mutated to arginines) instead of the wild-type (WT) ubiquitin with PRMT5 and CT47. As shown in Fig. 1F, HA-ubiquitin-K63 but not HA-ubiquitin-K48 supported polyubiquitination of PRMT5. Similarly, the induction of CT47 also reduced the polyubiquitination of PRMT5 mediated by HA-ubiquitin-K63. These results demonstrate that CT47 binds and stabilizes PRMT5 by blocking its K63-linked polyubiquitination.

### Expressing the HSG CT47 in mice impairs spermatogenesis

**Fig. 2:**
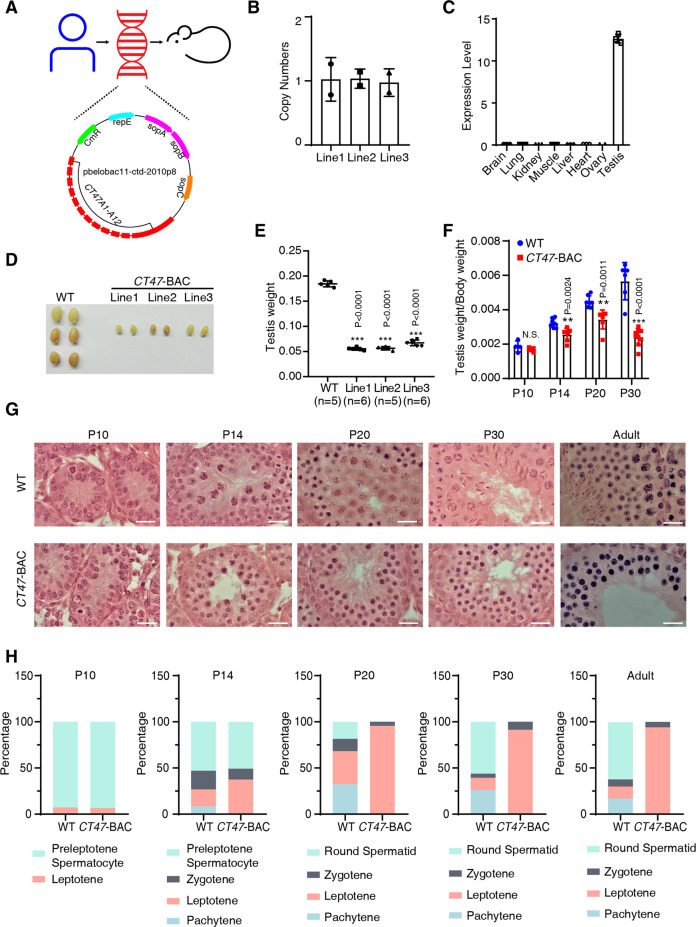
Meiotic maturation arrest in humanized *CT47*-BAC testes. **A** Generation of *CT47*-BAC transgenic mice. The 212 kb BAC clone ctd2010-p8 (containing human CT47.1 to CT47.12) was microinjected into mouse pronuclei. **B** Estimated CT47 transgene copy number in the three transgenic mouse lines estimated from qPCR data. Three independent experiments were conducted. **C** *CT47* expression level in 8 different tissues of *CT47*-BAC mice. The mean values ± standard deviation (SD) are shown. **D** Testis morphology of 2-month-old wild-type and three lines of *CT47*-BAC mice. **E** Testis weight of 2-month-old wild-type and three lines of *CT47*-BAC mice. The mean value ± SD is shown. Significance is shown by Student’s *t* test: WT (*n* = 5); Line 1 (*n* = 6); Line 2 (*n* = 5); Line 3 (*n* = 6), ***p < 0.001. **F** Relative testis weight (testis weight per g body weight) of WT mice (blue dot) and *CT47*-BAC mice (red dot) at postnatal Days 10, 14, 20, and 30. The mean values ± SD are shown. Significance is shown by Student’s *t* test, P10 WT (*n* = 4), *CT47*-BAC (*n* = 4); P14 WT (*n* = 7), *CT47*-BAC (*n* = 7); P20 WT (*n* = 7), *CT47*-BAC (*n* = 7); P30 WT (*n* = 6), *CT47*-BAC (*n* = 6) *p < 0.05, **p < 0.01. **G** Hematoxylin and eosin staining of wild-type and *CT47*-BAC testes at P10, P14, P20, P30, and adulthood (8 weeks). Scale bar = 20 μm. **H** Spermatocyte types were identified based on nuclear morphology and their positions within tubules. The proportion of spermatocyte types was analyzed in WT and *CT47*-BAC mice at P10, P14, P20, P30, and adulthood (8 weeks).

Recent studies have shown that humanized transgenic models have been successfully applied to provide more direct evidence for the functional importance of human evolution, including NOTCH2NL, SRGAP2, and ARHGAP11B [25–27]. These studies provide the first examples of HSGs that may be linked to human signatures. To elucidate the function of human CT47 in an in vivo model, we generated three independent humanized transgenic mouse lines bearing *CT47A1*-*A12* bacterial artificial chromosomes (BACs, *CT47*-BAC mice) through microinjection of CTD-2010P8 (vector pBeloBac11), which carries cis-acting genomic regulatory elements, into the pronucleus of one-cell mouse embryos [28] (Fig. 2A). Quantitative polymerase chain reaction (qPCR) analysis indicated that, on average, one copy of the transgene per mouse was successfully integrated into the genome (Fig. 2B). Similar to the *CT47* mRNA expression profile in human tissues [20], *CT47* was exclusively expressed in the testes of *CT47*-BAC mice (Fig. 2C). Furthermore, immunostaining showed that the CT47 protein was present in the testes throughout most stages of spermatogenesis in CT47 humanized mice (Fig. S2A).

Despite being healthy, the *CT47*-BAC males were sterile and failed to produce offspring when mated with C57BL/6 J females. In contrast, this sterility phenotype was not found in *CT47*-BAC females (Fig. S2B), in consistency with the absence of CT47 expression in the ovary (Fig. 2C). Consistent with its infertility phenotype, 2-month-old *CT47*-BAC males of all three lines showed a reduced size and weight of the testes (Fig. 2D, E). Their testes were significantly smaller than the testes of WT littermates from P14 onward and by P30 (Fig. 2F). Histological analysis of *CT47*-BAC and WT testes at various developmental time points (P10, P14, P20, P30, and adult), which reflect different stages of spermatogenesis [29], showed that *CT47*-BAC testes had severe abnormalities in their seminiferous tubules from P14 onward. Moreover, postmeiotic germ cells were scarce in *CT47*-BAC testes from P14 to adulthood (Fig. 2G, H). Specifically, *CT47*-BAC testes have only a few pachytene spermatocytes and spermatids with a large amount of leptotene and a few zygotene spermatocytes according to histological classifications (Fig. 2G, H). These results suggest that expressing human-specific CT47 in mouse testes impairs spermatogenesis at the meiosis stage.

To define which stage of spermatogenesis was arrested in *CT47*-BAC mice, we stained for γH2AX and SYCP3/lectin PNA at P30 testes from WT and *CT47*-BAC mice. Spermatocytes from P30 WT mice there are fully assembled synaptonemal complexes, which are typical of the pachytene stage, while in P30 *CT47*-BAC mice, only nuclei at early stages were observed with the typical simple-axis staining of leptotene (left), or only partially assembled synaptonemal complexes with portions of simple axes which looks like zygotene (right) (Fig. S3A, B). Altogether, these results suggest that *CT47*-BAC spermatocytes were arrested at an early stage of meiosis.

### CT47 in mice impedes leptotene/zygotene-to pachytene transition of meiosis I

**Fig. 3:**
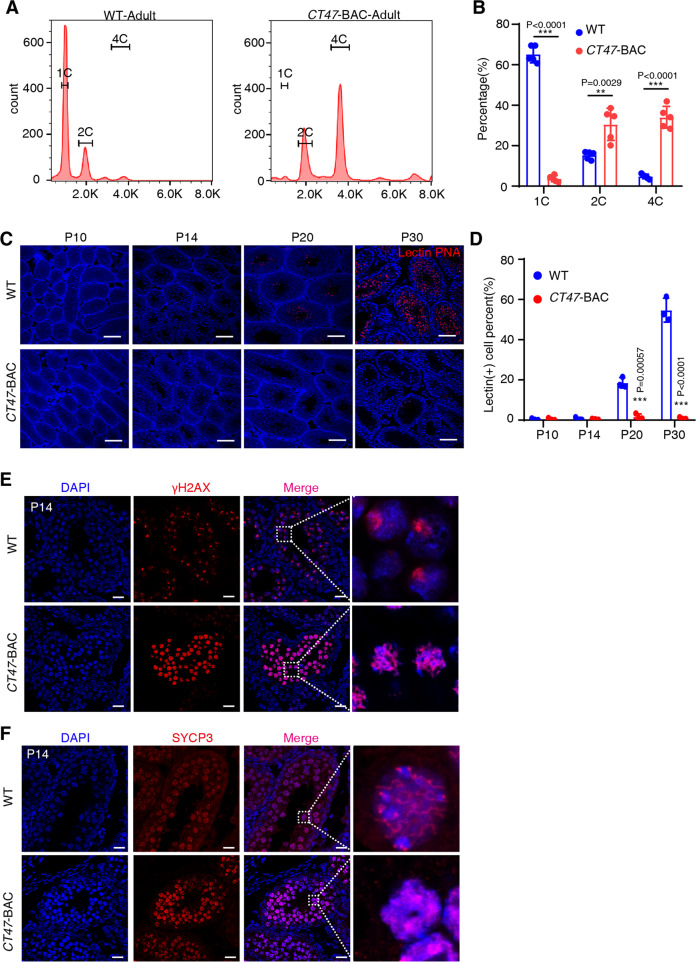
Meiotic arrest during LZ/P spermatocyte transition in *CT47*-BAC mice. **A** The proportion of different ploidy germ cells in WT and *CT47*-BAC mice analyzed by FACS according to the DNA content in spermatogenesis. **B** Statistical results of different cell types in the FACS assay. 1 C DNA content: different steps of round and elongating spermatids and spermatozoa. 2 C DNA content: G1-phase spermatogonia and secondary spermatocytes. 4 C DNA content: various stages of primary spermatocytes and G2-phase spermatogonia. The results shown are the mean ± SD, *n* = 5 for each group. Student’s *t* test: ***p < 0.001. **C** Lectin PNA Alexa Fluor 568 (red) in testis at P10, P14, P20, P30 WT, and *CT47*-BAC mice and scanned by TissueFAXS. **D** The proportion of lectin (+) cells was calculated. The results shown are the mean ± SD, *n* = 3 for each group. Student’s *t* test: ***p < 0.001, Scale bar = 40 μm. **E** Immunofluorescence staining for γ-H2AX in the testes of P14 WT and *CT47*-BAC mice. Scale bar = 40 μm. Also see immunofluorescence staining for SYCP3 in Fig. 6C. **F** Immunofluorescence staining for SYCP3 in the testes of P14 WT and *CT47*-BAC mice. Scale bar = 40 μm. Also see immunofluorescence staining for SYCP3 in Fig. 6D.

To further determine the substage of spermatocytogenesis at which meiotic arrest occurs in *CT47*-BAC testes, we first used flow cytometry to examine the effect of CT47 expression on the germ cell composition of the testes. As expected, *CT47*-BAC testes showed a drastic reduction in the proportion of 1 C DNA content cells and a significant increase in the proportions of 4 C DNA content cells (Fig. 3A, B), supporting that germ cell composition changed significantly in *CT47*-BAC mice. When visualized by immunostaining with peanut agglutinin (PNA) lectin, a marker for the acrosome, it was clear that *CT47*-BAC testes had almost no round spermatids compared with wild-type (WT) mice (Fig. 3C, D). These results together with histological analysis (Fig. 2G, H) indicate that CT47 expression in testes specifically caused spermatogenesis arrest at meiotic prophase I.

Furthermore, *CT47*-BAC and WT testes were collected at P14 and stained with γ-H2AX antibody, a key marker for the progression of meiotic prophase I. In the WT mice, the γ-H2AX signal was confined to the chromatin of sex chromosomes, whereas in *CT47*-BAC mice, the γ-H2AX signal was distributed throughout the nucleus (Fig. 3E and Fig. S4A). Furthermore, SYCP3 staining and chromosome spread slide for SYCP3 showed thread-like fibers in the WT mice, while SYCP3 pattern exhibited aggregated and irregular fibers in *CT47*-BAC mice (Fig. 3F and Fig. S4A). A consistent result was obtained followed by fluorescence-activated cell sorting (FACS) labeled with γ-H2AX antibody in P14 WT and *CT47*-BAC testes. These γ-H2AX foci appeared in WT mice but were absent in *CT47*-BAC mice counterstained by the VASA signal (Fig. S4B). None of these spermatocytes displayed the typical pachytene distribution of the γ-H2AX and SYCP3 signal (Fig. 3E, F and Fig. S4B).

Notably, immunostaining for ZBTB16, a marker of undifferentiated spermatogonia, indicated no significant difference in the number of spermatogonia between WT and *CT47*-BAC testes (Fig. S4C, D). Altogether, these results indicate that CT47 expression in testes specifically impedes spermatogenesis during leptotene/zygotene spermatocyte transition to pachytene spermatocyte after spermatogonia development.

We also examined cell proliferation using immunostaining to detect histone H3 phosphorylation (pHH3) at serine 10 in WT and *CT47*-BAC testes at P10, P14, P20, and P30. The percentage of pHH3-positive cells in seminiferous tubules also did not differ between *CT47*-BAC and WT testes (Fig. S5A,B), suggesting that germ cell proliferation is less affected in *CT47*-BAC testes.

In addition, the TUNEL assay, in which the labeled apoptotic cells were scanned by TissueFAXS high-throughput acquisition and automated analysis, revealed the presence of more apoptotic cells in *CT47*-BAC testes than in WT testes from P14 onward (Fig. S6A, B), indicating that meiotic arrest appears in *CT47*-BAC mice [30, 31].

### CT47 enhances PRMT5 accumulation in the nucleus of leptotene/zygotene spermatocytes

**Fig. 4:**
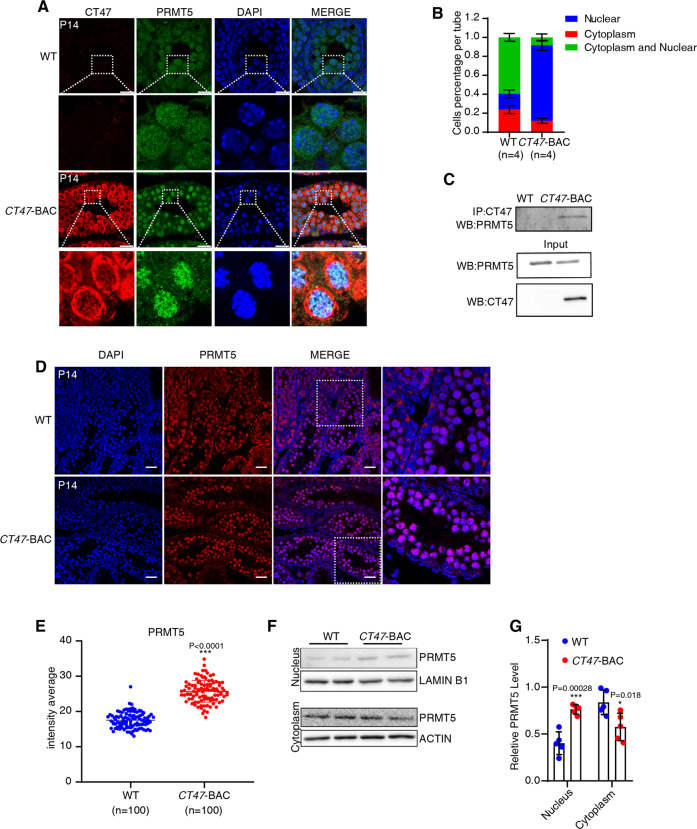
CT47 promotes the nuclear retention of PRMT5 in leptotene spermatocytes. **A** Coimmunofluorescence staining for PRMT5 (green) and CT47 (red) in P14 WT and *CT47*-BAC testes. Nuclei were stained with DAPI. Scale bar = 20 μm. **B** Quantification for cytoplasmic and nuclear localization of PRMT5 per tube in P14 WT and *CT47*-BAC mouse testis. The results shown are the mean values ± SD. *n* = 4 for each group. **C** Co-immunoprecipitation of CT47 followed by Western blot detection of PRMT5 from P14 WT and *CT47*-BAC testes. WB, western blot. **D** Immunofluorescence staining for PRMT5 in P14 WT and *CT47*-BAC testes. Scale bar = 30 μm. Also see Fig. S7. **E** Quantification for intensity average for nuclei PRMT5 in P14 WT and *CT47*-BAC mouse testis. The results are the mean values ± SD. *n* = 100 for each group, ***p < 0.001. **F**, **G** Cytoplasmic and nuclear fractions of P14 WT and *CT47*-BAC testes were separated by an active motif kit. The lysates were subjected to Western blotting. The relative PRMT5 level was measured. The results shown are the mean values ± SD. Student’s *t* test: *n* = 5 for each group, ***p < 0.001, *p < 0.05.

Based on the above results, we next investigated whether the CT47-PRMT5 interaction might function as a meiotic gatekeeper controlling the leptotene/zygotene-to-pachytene progression of spermatogenesis [32]. Analysis of the subcellular localization of CT47 and PRMT5 in P14 *CT47*-BAC mouse testes found that CT47 was present in both the cytoplasm and nucleus of meiotic spermatocytes, whereas PRMT5 is obviously retained in the nucleus in CT47 testes, in contrast, to control testes (Fig. 4A, B). We further confirmed the interaction of these two proteins with an immunoprecipitation assay using lysates from *CT47*-BAC testes (Fig. 4C). The fluorescence intensity of PRMT5 accumulation in CT47 nucleus was significantly higher than the in WT nucleus (Fig. 4D, E). This finding was further confirmed by western blotting of cytoplasmic and nuclear fractions of P14 *CT47*-BAC testes (Fig. 4F, G). Finally, co-immunostaining of γH2AX and PRMT5 in *CT47*-BAC and WT testes at P30 showed that PRMT5 signal was weaker in pachytene spermatocytes than in leptotene spermatocytes in WT testes, while PRMT5 signal is obviously stronger in *CT47*-BAC testes than in WT testes (Fig. S7). Together, these results showed that the retention of PRMT5 by expressed CT47 in the nucleus of spermatocytes is highly related to meiosis arrest.

### CT47 inhibits meiotic-related gene expression

**Fig. 5:**
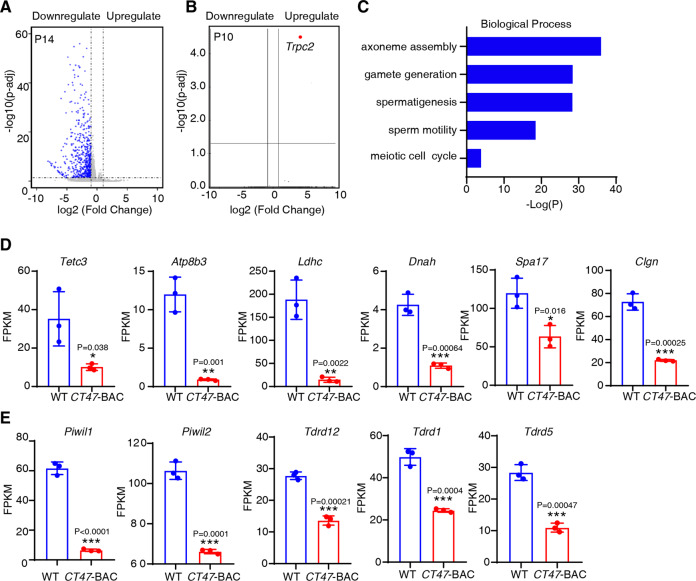
Pachytene-related genes were significantly repressed in *CT47*-BAC mice. **A**, **B** Volcano plot representing differentially expressed genes in the RNA-seq of P14 (**A**) and P10 (**B**) in the indicated testes. Volcano plots for three biological replicates of each genotype are shown. Blue dots represent 539 downregulated genes of P14 *CT47*-BAC testes. Red dots represent one upregulated gene of P10 *CT47*-BAC testes. The *P*-adj < 0.05 and log2-fold change ≥1 cutoff values. **C** Gene ontology functional enrichment (GO) of the DEGs arising from P14 *CT47*-BAC over WT testes according to the −log (*p* value). **D** RNA-seq analysis of selected genes related to pachytene expression. The mean values ± SD are shown. Student’s *t* test: *n* = 3 for each group, *p < 0.05, ***p < 0.001. **E** RNA-seq analysis of piRNA-related genes. The mean values ± SD are shown. Student’s *t* test: *n* = 3 for each group, *p < 0.05, **p < 0.01, ***p < 0.001.

PRMT5, an arginine methyltransferase, exerts a repressive epigenetic effect on chromatin structure and retrotransposon element silencing [23, 33, 34]. Thus, the CT47-PRMT5 interaction may limit the methyltransferase activity of PRMT5, thereby decreasing gene transcription during spermatogenesis. To test this hypothesis, we performed RNA-seq on testes from WT and *CT47*-BAC mice at P10 and P14. A total of 18359 expressed genes were found in these samples. The lack of differential expression at P10 is consistent with the normal spermatogenesis observed in *CT47* testes at this stage (Fig. 5B), suggesting that CT47 functions at stages beyond P10. In the P14 testes, we identified 539 differentially expressed genes (DEGs) (adjusted *p* value < 0.05 and fold change >2) (Table S2), all of which were downregulated in the *CT47*-BAC testis (Fig. 5A). Gene Ontology (GO) analysis of DEGs using Metascape revealed 13 enrichment clusters that were associated with various cellular or developmental processes, including axoneme assembly, male gamete generation during spermatogenesis, meiotic cell cycle, and sperm motility (Fig. 5C, Benjamini–Hochberg *p* value correction algorithm with *p* < 0.05 or -log p > 1.3 as significant difference). Basically, these enriched GO terms were related to pachytene spermatocyte (PS)/round sperm (RS) [35]. Notably, *Tetc3*, *Atp8b3*, *Ldhc*, *Dnah*, *Spa17*, and *Clgn* expressed in the pachytene stage were significantly repressed in *CT47*-BAC mice (Fig. 5D). Other DEGs profoundly suppressed in the *CT47*-BAC testes at P14 were Piwi-like RNA-mediated gene silencing 1 (*Piwil1*) genes, including Tudor-domain-containing *Tdrd5*, *Tdrd1*, and *Tdrd12* (Fig. 5E), which were previously implicated in the biogenesis of PIWI-interacting RNA (piRNA) and its associated silencing of transposable elements in spermiogenesis [36, 37]. Together with their annotations in spermatogenesis, we suggest that CT47 expression likely enhances meiotic arrest before pachytene stage spermatocytes at the molecular level.

### Testosterone partly rescues the arrest of leptotene/zygotene development in *CT47*-BAC mice

Given that CT47-mediated retention of PRMT5 in the nucleus leads to leptotene/zygotene arrest, we predicted that the disassociation of CT47 from PRMT5 should facilitate leptotene/zygotene progression [38]. The presence of CT47 in humans but not in mice suggests differences in spermatogenetic processes between humans and rodents. Moreover, testosterone is known to exert distinct effects on spermatogenesis in humans and mice [6]. In humans, testosterone functions as a critical regulator of the first wave of meiosis in spermatogenesis because human spermatogenesis does not proceed beyond the leptotene-stage of meiosis until the testosterone level surges at puberty [9, 11, 12, 14, 39]. Therefore, we investigated whether the interaction between CT47 and PRMT5 is sensitive to testosterone. To avoid excess testosterone effects, we used gradient doses of testosterone treatment at doses of 50 ng/g/day and 5000 ng/g/day (diluted to 0.5 mg/ml in corn oil). *CT47*-BAC and WT mice from P10 to P14 were injected with testosterone propionate subcutaneously. Note that without treatment, the testosterone levels of *CT47*-BAC and WT testes at P14 were similar (Fig. S8A), indicating that the observed leptotene arrest of spermatogenesis in the *CT47*-BAC mice is not very likely due to low testosterone levels. In addition, we noticed that 50 ng or 5000 ng doses did not induce a significant difference in intratesticular testosterone concentrations (Fig. S8A).

**Fig. 6:**
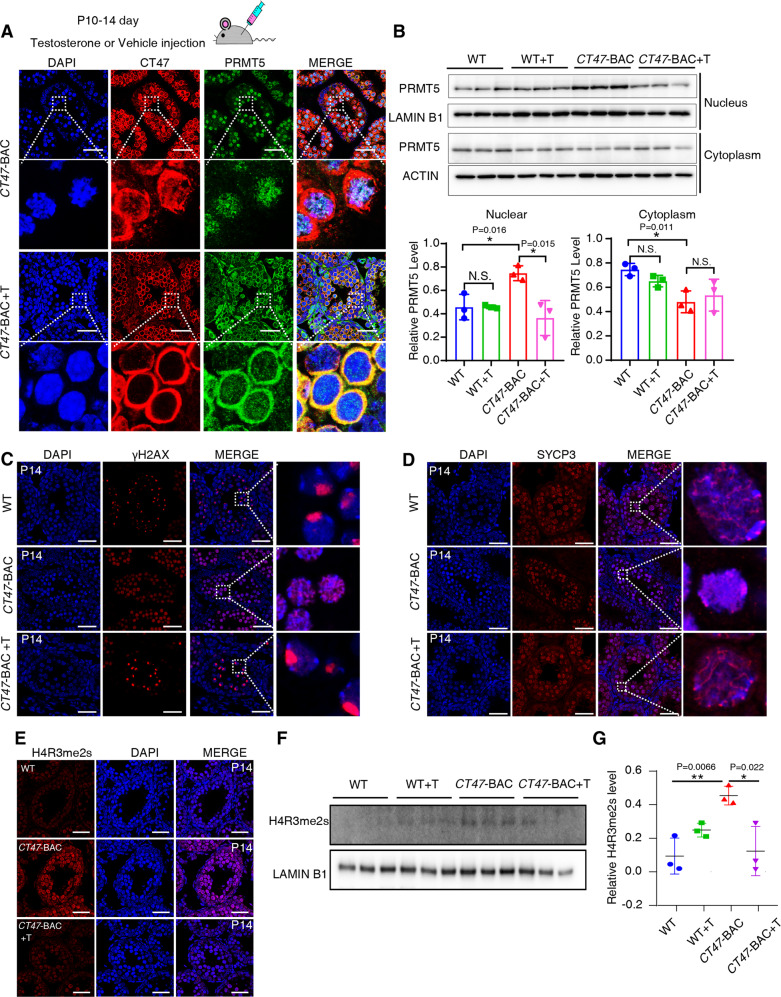
Testosterone treatment rescues meiotic arrest in *CT47*-BAC mice and causes nuclear depletion of CT47/PRMT5. **A** Coimmunofluorescence staining for PRMT5 (green) and CT47 (red) in P14 testes from corn oil-treated or testosterone(T)-treated *CT47*-BAC mice. Scale bar = 40 μm. Nuclei were stained with DAPI. **B** Cytoplasmic and nuclear fractions of testes were separated from corn oil-treated WT, *CT47*-BAC, or testosterone(T)-treated WT, *CT47*-BAC mice. Subsequent detection of PRMT5 was performed using anti-PRMT5 antibodies. The relative PRMT5 value represents the mean ± SD, *p < 0.05, *n* = 3 mice per group. **C**, **D** Immunofluorescence staining for γ-H2AX and SYCP3 in the testes of P14 WT, *CT47*-BAC, or testosterone-treated *CT47*-BAC mice. Scale bar = 40 μm. **E** Immunofluorescence staining for H4R3me2s in the testes of P14 WT, *CT47*-BAC, or testosterone-treated *CT47*-BAC mice. Scale bar = 40 μm. **F**, **G** H4R3me2s was detected in corn oil-treated WT and *CT47*-BAC or testosterone-treated WT and *CT47*-BAC testes. The relative H4R3me2s level was measured. The mean values ± SD are shown. Student’s *t* test: *n* = 3 for each group, *p < 0.05, **p < 0.01.

Remarkably, after testosterone treatment, immunostaining revealed that PRMT5 was dramatically depleted in the nucleus and became enriched in the cytoplasm of *CT47*-BAC spermatocytes (Fig. 6A). Additionally, cytoplasmic colocalization of CT47 and PRMT5 became evident, with CT47 found only in the cytoplasm and no longer detected in the nuclei of *CT47*-BAC spermatocytes after testosterone treatment (Fig. 6A). Immunoblotting results of nuclear and cytoplasmic cell fractions further confirmed the immunostaining results: the abundance of nuclear PRMT5 in the *CT47*-BAC testes was markedly reduced after testosterone treatment (Fig. 6B). Testosterone treatment had no significant effect on PRMT5 localization in WT testes (Fig. 6B), indicating that the effect of testosterone on PRMT5 localization in *CT47*-BAC testes was dependent on CT47 expression, not a direct effect on PRMT5, which explains why the absence of high endogenous testosterone emergence in the presence of CT47 prevents PRMT5 nuclear shuttling in mice. Together, these results indicate that testosterone may promote CT47 cytoplasmic localization, which then induces the nuclear exit of PRMT5.

To confirm whether testosterone interference also causes the nuclear depletion of PRMT5 in human cells, we employed androgen-sensitive human prostate adenocarcinoma LNCaP cells that can take up testosterone and express endogenous CT47. In agreement with the above results, the retention of nuclear PRMT5 was significantly decreased in LNCaP cells in response to exogenous testosterone (Fig. S8B and S8C), supporting the hypothesis that testosterone destabilizes nuclear PRMT5/CT47 in human cells.

Based on these findings, we hypothesized that testosterone-induced PRMT5 cytoplasmic localization would relieve its repressive epigenetic regulatory impacts on the progression of spermatogenesis beyond leptotene. To examine this hypothesis, we stained testis sections from P14 vehicle- or testosterone-treated *CT47*-BAC and WT mice using antibodies against the meiotic markers γ-H2AX and SYCP3. Whereas untreated WT testes exhibited strong asymmetrically localized γ-H2AX foci at the sex chromatin, as would be expected in a normal pachytene stage spermatocyte [40], γ-H2AX foci in the vehicle-treated *CT47*-BAC testes were distributed evenly throughout the nucleus. Strikingly, nuclear γ-H2AX expression in testosterone-treated *CT47*-BAC testes showed an asymmetric distribution similar to the distribution in WT spermatocytes (Fig. 6C). Similarly, the SYCP3 signals appeared as aggregated and irregular fibers in the spermatocytes of vehicle-treated *CT47*-BAC testes but as fine threads in the spermatocyte nucleus of both the vehicle-treated WT and testosterone-treated *CT47*-BAC testes (Fig. 6D). The short fine nuclear thread-like distribution of SYCP3 indicates normal pachytene development of the WT spermatocyte [41]. Together, these results indicate that testosterone-induced cytoplasmic localization of CT47 and nuclear depletion of PRMT5, phenotypically and functionally rescue the leptotene-stage meiotic arrest of *CT47*-BAC spermatocytes.

Since PRMT5 mediates the methylation of arginine-3 of histone H4 (H4R3me2s), an epigenetic regulatory modification [33], we examined the H4R3me2s level in WT and *CT47*-BAC mice with or without testosterone treatment by immunostaining and western blot analysis. The H4R3me2s levels were markedly decreased in the testosterone-treated *CT47*-BAC testes to a level similar to the level in the WT testes (Fig. 6E–G). The effect of testosterone was specific to *CT47*-BAC testes, as testosterone treatment did not decrease H4R3me2s levels in WT testes (Fig. 6F, G). These results suggest that the testosterone rescue of the leptotene-stage meiotic arrest phenotype of *CT47*-BAC spermatocytes is due to the release of PRMT5-mediated epigenetic suppression.

### CT47 mediates the testosterone-induced promotion of meiosis entrance in human cells

**Fig. 7:**
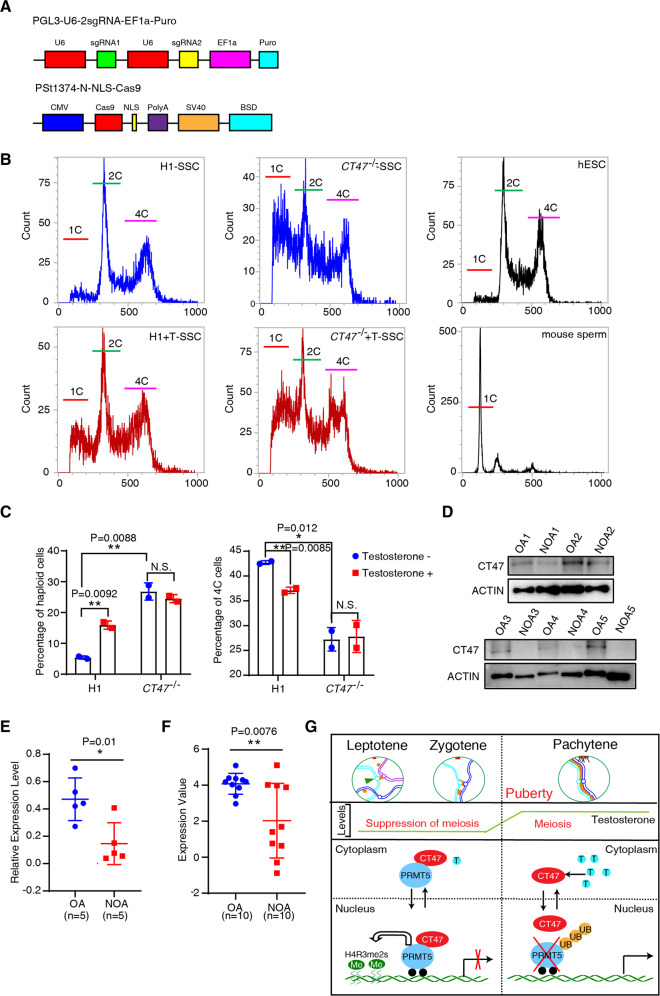
Human infertility is associated with low levels of CT47 expression. **A** The procedures for generating CT47 mutations in hESCs. A plasmid system for sgRNA transcription and Cas9 expression. PGL3-U6-2 sgRNA-EF1a-Puro for sgRNA transcription and PSt1374-N-NLS-Cas9 for Cas9 expression. **B** FACS ploidy analysis of the haploid spermatogenic cell percentage of H1 and *CT47*^*−/−*^ SSCs in SSC culture with 200 pM testosterones. hESC and mouse sperm were used as control groups. **C** The results shown are the mean ± SD from two independent technical replicates in the indicated groups. Student’s *t* test: *p < 0.05, **p < 0.01, N.S., no significant difference. **D** CT47 protein levels in human testicular biopsy samples from five nonobstructive azoospermia (NOA 1–5) and obstructive azoospermia (OA 1–5) patients. **E** Quantification of the western blot in **D**. The results shown are the mean ± SD, *n* = 5 in each of the two groups. Student’s *t* test: *p < 0.05. **F** CT47 expression levels from GSE145467. OA, obstructive azoospermia. NOA, Nonobstructive azoospermia. The results shown are the mean ± SD. Student’s *t* test: *n* = 10 per group, **p < 0.01. **G** Schematic model showing that the dynamics of CT47 translocation and interaction with PRMT5 synchronizes the meiotic process of spermatocytes with testosterone during puberty.

Our results above from the humanized mouse model suggest that human-specific *CT47* is an essential regulator of human spermatogenesis. To further determine the human meiosis relevance of this CT47 function responding to testosterone, we next performed a loss-of-function experiment in H1 human embryonic stem cell lines (H1 hESCs) using the CRISPR/Cas9 system (Fig. 7A). As CT47 is characterized by an array of tandem repeats with similar sequence identity, a suitable sgRNA can detect all potential similar sequences that would cleave the gene set with efficacy. Mapping of *CT47* knockout (*CT47*^−/−^) colonies by sequencing a single peak in the target sequence strategy allowed us to distinguish the null allele from the mosaic colonies (Fig. S9A). To further verify *CT47* knockout efficiency, we need to examine the expression level of *CT47* mRNA. However, *CT47* itself is very low expressed in ES cells, we thus proceeded to differentiate hESCs into human embryoid bodies (hEBS) and then compared *CT47* mRNA levels in control and *CT47*^*−/−*^ hEBs by real-time qPCR. The results showed that the *CT47* expression level was significantly downregulated in *CT47*^−/−^ hEBs (Fig. S9B). To exclude the possibility of widespread off-target mutations in these hESCs, we mapped and located, using an RNA-seq-based approach [42], as much as 75%-85% of the RNA single nucleotide variations (SNVs) identical in both KO and H1 hESCs. Importantly, among all the predicted potential off-target sgRNA binding sites, no single nucleotide variation (SNVs) was found to match off-target loci in the *CT47*^−/−^ cells (Fig. S9C). In addition, *CT47*^−/−^ hESCs displayed a relatively normal karyotype compared with H1 hESCs (Fig. S9D). *CT47*^−/−^ hESCs showed an undifferentiated phenotype, as evidenced by further staining with alkaline phosphatase (Fig. S9E). The expression levels of the pluripotency factors *OCT4*, *SOX2*, and *NANOG* were comparable in both *CT47*^−/−^ and H1 hESCs (Fig. S9F). Together, these results indicate that *CT47*^−/−^ hESCs showed significantly downregulated *CT47* expression levels and retained typical stem cell characteristics.

Then, we examined the ability of *CT47*^*−/−*^ hESCs to differentiate into spermatogonial stem cell (SSC)-like cells in the presence or absence of testosterone [43]. After 10 days of culture, we observed significant VASA-positive cells. These VASA^+^ germ-like cells showed typical VASA staining patterns (Fig. S10A, B), as seen in previous reports [43] and lectin PNA was employed to assess the sperm acrosomal status and the acrosome reaction (Fig. S10C). These results suggested that we successfully differentiated hESCs into germ cell lineages. We found that differentiation in SSC conditions yielded a small percentage of haploid spermatogenic cells (1 C) after a 10-day culture with SSC differentiation medium; however, the absence of *CT47* resulted in a 5-fold increase in haploid spermatogenic cells (1 C DNA content) (28.8% for *CT47*^*−/−*^ cultures vs. 5.7% for H1 cultures) and decrease in the proportion of cells with 4 C DNA content (27.245% for *CT47*^−/−^ cultures vs. 42.72% for H1 cultures) by flow cytometric analysis, indicating that CT47 protein regulates the entry of H1 hESCs into meiosis (Fig. 7B, C). Strikingly, the presence of testosterone dramatically increased the proportion of haploid spermatogenic cells in H1 hESC cultures (5.7% to 16.8%; Fig. 7B, C). In contrast, testosterone had no impact on the formation of haploid spermatogenic cells from *CT47*^*−/−*^ hESCs (Fig. 7B, C). These results, consistent with the testosterone-induced spermatogenesis effects we observed in *CT47*-BAC mice, provide further evidence that CT47 plays an important role in testosterone-induced meiosis entry of human cells.

### Low CT47 level is linked to human infertility

To examine the functional importance of CT47 expression in human spermatogenesis, we collected and analyzed the CT47 protein levels in human testicular biopsy samples from five idiopathic nonobstructive azoospermia (NOA) patients and five obstructive azoospermia (OA) patients. None of these patients had a history of orchitis or obstruction of the vas deferens or had received hormone therapy (Table S1). As shown in Fig. 7D, E, the CT47 protein levels were markedly lower in the samples from idiopathic NOA patients than in the samples from OA patients. To confirm this result, we also analyzed the data for 10 idiopathic NOA and 10 OA patients in a recently reported Agilent Human Genome Microarray dataset (GSE145467). Comparison of the two patient groups revealed that the *CT47* mRNA levels in the NOA patients were significantly lower than the *CT47* mRNA levels in the OA patients (*p* < 0.01; Fig. 7F). As high heterogeneity and limitation of sampling in human biopsies may greatly affect the reliability of differential expression results, our comparison analysis can be clearly seen from the changes in CT47 levels in the NOA patients compared to the CT47 levels in the OA patients. Together with our observations from cultured human cells and *CT47* BAC mice, these results demonstrate that a lack of sufficient CT47 in human testes may lead to failure to respond to testosterone or failure in the maintenance of the prespermatogenesis stage.

## Discussion

In this study, we combined coIP mass spectrometry with a humanized mouse model and hESC differentiation to identify the physiological role of the HSG *CT47*. Multiple lines of evidence from the present study support the functional role of CT47 in human spermatogenesis. First, we created a humanized *CT47* mouse model by integrating a 210-kb *CT47*-containing human BAC into the mouse genome which resulted in leptotene spermatocyte arrest and animal sterility. Second, the phenotype of leptotene spermatocyte arrest in *CT47*-BAC mice could be partially rescued by testosterone treatment, which is reminiscent of human spermatogenesis in which meiosis proceeding beyond leptotene does not occur until the testosterone level surges at puberty, suggesting a gatekeeper role of CT47 during spermatogenesis. Third, in vitro hESC differentiation assays revealed that *CT47* KO caused defects in hESC differentiation with greatly enhanced haploid spermatogenic cells, suggesting that the concertation of CT47 is important in human spermatogenesis. Finally, we showed that human idiopathic azoospermia disorders are associated with low levels of CT47 expression, which might decrease testosterone responsiveness at puberty.

Our study also provides critical mechanistic insights into how CT47 functions in the testosterone-regulated spermatogenesis process: CT47 binds and regulates the critical epigenetic regulator PRMT5. CT47 effectively stabilizes PRMT5 by preventing its K63 ubiquitination and proteasome-mediated degradation in human cells. Consistent with these results, in humanized *CT47*-BAC mice, CT47 and PRMT5 colocalized in the testis, and degradation of PRMT5 was inhibited, resulting in a high nuclear PRMT5 accumulation during the leptotene/zygotene-to-pachytene transition.

In vertebrates, the hypothalamic-pituitary-gonadal axis is responsible for the neuroendocrine control of reproductive processes. Although spermatogenetic development is similar across different mammalian species, different species have evolved distinct pathways to achieve optimal reproductive fitness [44]. Although the testosterone level is low until P24 in rodents [45], spermatogenesis occurs as a consecutive process starting from birth. In humans, gonad-localized steroid hormone production during pubertal development is coupled with brain maturation [46]. The initiation of meiosis in human spermatogenesis is evoked by a hypothalamic-pituitary-gonadal axis after a surge in the testosterone level at puberty following a long period of the “prespermatogenesis” stage [47–49], suggesting that testosterone-triggered meiotic initiation after puberty allows optimal male sexual maturation in men.

We showed that testosterone treatment of humanized *CT47*-BAC mice caused a dramatic reduction in nuclear CT47 and enrichment of cytoplasmic CT47 in the testis. Consequently, the PRMT5 level dramatically decreased in the nucleus, resulting in the rescue of the leptotene spermatocyte arrest phenotype in *CT47*-BAC mice. However, although testosterone allows meiosis to occur, *CT47*-BAC mice are still sterile, suggesting that the function of CT47 is not only limited to an early meiosis arrest. Similarly, testosterone treatment of androgen-sensitive human prostate LNCaP cells also caused a decrease in the CT47 level and nuclear depletion of PRMT5, indicating the relevance of such a mechanism in human cells. However, how testosterone treatment induces a CT47 decrease and the nuclear shuttling of PRMT5 remains unclear. CT47 inhibited hESC differentiation into haploid spermatogenic cells, suggesting that CT47 blocks the entry point into meiosis and contributes to the maintenance of the prespermatogenesis stage and, therefore, the readiness to progress to the subsequent stages. We thus propose that at puberty, the testosterone surge (evoked by pituitary gonadotropins) releases the function of CT47, resulting in a commitment to the first wave of meiosis (Fig. 7G). Together, these results establish the testosterone-induced nuclear depletion of CT47/PRMT5 in the testes as an essential mechanism that regulates human spermatogenesis and demonstrates the critical role of CT47 in regulating the epigenetic functions of PRMT5. Although how testosterone triggers the shuttling of CT47 between the cytoplasm and nucleus remains to be investigated, the AR pathway and the hypothalamic-pituitary-gonadal axis are likely involved [9, 10, 13, 15, 47]. As testosterone treatment did not rescue the late pachytene stage onward, it is likely that multiple epigenetic and hormonal events cooperatively and sequentially regulate spermatogenesis.

*CT47* is expressed in human testes. However, CT47 has also been shown to be activated in some cancers, such as lung, esophageal and endometrial cancer [20, 21]. PRMT5 has previously been implicated in oncogenic processes, regulating the methylation of histones and other regulatory proteins involved in cell cycle progression, cell death, and metabolism [50–52]. Because of the potent role of CT47 in regulating PRMT5 function, CT47 may also function as an oncogene and contribute to tumorigenic processes. In the present study, we also showed that CT47 could associate with proteins other than PRMT5, including heat shock proteins, microtubule proteins, a mitochondrial protein, and a ubiquitin-protein ligase protein. The functional importance of these interactions has not been verified. They may be involved in CT47/PRMT5 regulation or other PRMT5-independent functions of CT47.

In our analysis of clinical samples, we also detected a strong correlation between the significantly diminished level of CT47 in human idiopathic azoospermia. Although the precise underlying mechanisms warrant further investigation, diminished CT47 expression in patient testes may dampen the functional response to testosterone involving nuclear-to-cytoplasmic translocation of PRMT5 for the initiation of spermatogenesis in adults, resulting in azoospermia. Alternatively, diminished CT47 levels may result in defective gatekeeping of nuclear PRMT5 in prespermatogenesis before puberty. Conversely, the selectivity with which CT47 mediates the effects of testosterone on spermatogenesis without alteration in androgen levels provides a new target for male anticonception without side effects related to the peripheral effects of testosterone or effects on libido, which may explain why humans need so many CT47 copies and work together to perform the same functions.

Although the lack of a mouse ortholog makes the exploration of HSGs function challenging, our study lays a foundation for future HSG research. By combining the creation of hESC KO and in vitro hESC differentiation assays, the potential functional involvement of HSGs can be uncovered. Afterward, humanized HSG-specific mouse models together with immunoprecipitation experiments can be employed to determine the in vivo function of an HSG by analysis of their interactions with other essential genes (PRMT5 in this case), with the results further confirmed by studies in human cell lines. Our study demonstrates that despite genetic limitations, the combination of cellular, biochemical, and mouse genetic approaches is a powerful strategy for determining the functional importance of HSGs. While it is unlikely that every single HSG is functionally indispensable, the CT47 function and its mechanism demonstrated in the present study further support the notion that newly evolved genes gain functions by integrating into already existing biomolecular networks in cells to achieve optimal organismal fitness [17, 18, 53]. Therefore, the identification of HSG-interacting proteins represents a very informative approach to deciphering the potential functions of HSGs.

## Materials and methods

### Human samples

The hospital Medical Ethics Review Board approved the studies involving human subjects, and written informed consent was obtained from the patients. Five patients with idiopathic azoospermia and five control individuals were recruited from the Infertility Clinic affiliated with Nanjing Medical University. All clinical samples were collected with informed consent and approved by the Research Ethics Committee of Jinling Hospital. The approval number is 2015NZKY-017-02. The consent has been uploaded as supplemental material.

### Cell lines and culture conditions

HEK293T and Cos7 cells were grown in Dulbecco’s modified Eagle’s medium (DMEM)/high glucose containing 10% fetal bovine serum (FBS), 50 U/ml penicillin, and 50 μg/ml streptomycin. The H1 hESC line was maintained in mTeSR^TM1^ media in dishes precoated with Corning Matrigel hESC-Qualified Matrix along with 50 U/ml penicillin and 50 μg/ml streptomycin. All cell lines were maintained under standard conditions (37 °C, 5% CO_2_).

### Generation of gene-edited hESCs

The plasmids for the expression of *Streptococcus* pyogenes Cas9 and sgRNA were described previously [54]. In brief, the Cas9 expression plasmid pST1374-Cas9-N-NLS-Flag linker (Addgene 44758, Cambridge, MA, USA) has been optimized for nuclear import by two nuclear localization sequences and contains cytomegalovirus (CMV) promoter for expression in human cells. Furthermore, this plasmid also contains a blasticidin selection cassette for obtaining transfected cells expressing Cas9. Guide RNA was designed using WGE (Wellcome Sanger Institute Genome Editing) (https://www.sanger.ac.uk/htgt/wge/). Paired gRNAs targeting the flanking regions of each gene were designated as forward and reverse primers, respectively. The insert cassette (Guide RNA1 + scaffold RNA + U6 + Guide RNA2) obtained by PCR was cloned into the sgRNA plasmid (pGL3-U6-sgRNA-EF1a-Puro), which contained a puromycin selection cassette and a type II restriction enzyme Esp3I for linearization. The resulting dual-sgRNA plasmids for each targeted gene were inserted into H1 hESCs by electroporation with PSt1374-N-NLS-Cas9, and the colonies arising from single hESCs were picked under puromycin and blasticidin selection. Targeted regions were amplified by PCR from gDNA as the template, followed by sequencing.

### Generation of cell lines with DOX-inducible CT47 expression

The pPBH-TRE-tight vector for Tet-inducible eukaryotic protein expression was used to construct a *CT47* expression vector. Between the inverted terminal repeat sequences, the plasmid contained the hygromycin-resistant gene expression cassette for eukaryotic selection. The 5’ end of the hygromycin resistance cassette contained a TRE-tight promoter-driven expression cassette. The CT47 coding DNA sequence with a GFP fusion tag was inserted into the multiple cloning sites. pPBH-TRE-tight-CT47-EGFP with pSPB-transposase was cotransfected into COS7 cells using Lipofectamine 2000 (Invitrogen). The PiggyBac transposase will recognize the inverted terminal repeat and insert the DNA sequence between these terminal repeats into TTAA sites of the host cell chromosomes, allowing site-specific integration of plasmid DNA with high efficiency. To induce *CT47* expression, 2 μg/ml DOX was added to the culture medium.

### Generation of *CT47*-BAC transgenic mice

Ctd-2010p8 is a BAC clone from the human genomic library containing the complete human *CT47* copies from *CT47*.1 to *CT47*.12 on a 212-kb genomic region, purchased from Children’s Hospital Oakland Research Institute. The BAC clone was then microinjected into fertilized mouse eggs of a mixed genetic background of C57BL/6 J and 129/Ola. The founders were backcrossed into C57BL/6 J background to obtain transgenic mice. We characterized three independent lines based on their copy numbers by qPCR spanning 10 kb covering the *CT47* locus. All mice were housed in a specific pathogen-free animal facility. The animal protocol was approved by the Animal Care and Use Committee of Cam-Su Genomic Resource Center, Soochow University. The approved number is YX-2021-1 and YX-2017-3. Animal study protocol has been uploaded as supplemental material.

### H1 hESC culture

H1 hESCs were maintained in mTeSR^TM1^ media on dishes precoated with Corning Matrigel hESC-Qualified Matrix under standard conditions (37 °C, 5% CO_2_).

### Human SSC differentiation and FACS

Human SSC differentiation was carried out as previously described [43]. Briefly, H1 hESCs were cultured for 10 days in SSC medium consisting of minimum essential medium (MEM) alpha containing 0.2% bovine serum albumin, 5 mg/ml insulin, 10 mg/ml transferrin, 60 mM putrescine, 2 mM L-glutamine, 50 mM β-mercaptoethanol, 1 ng/ml human bFGF, 20 ng/ml GDNF, 30 nM sodium selenite, 2.36 mM palmitic acid, 0.21 mM palmitoleic acid, 0.88 mM stearic acid, 1.02 mM oleic acid, 2.71 mM linoleic acid, 0.43 mM linolenic acid, 10 mM HEPES, and 0.5X penicillin/streptomycin. To detect haploid cells, H1 SSCs were stained with propidium iodide (PI) and run on a FACS Aria sorter (BD Biosciences).

### Mass spectrometry-based tandem affinity purification

HEK293T cells from 24 mm × 150-mm dishes were transfected separately with pCMV-CT47-C-3×FLAG. HA plasmid or the pCMV-tag2b plasmid using Lipofectamine 2000 according to the manufacturer’s instructions. Cell lysates were obtained after centrifugation at 4 °C for 10 min at 13,000 × *g*. Immunoprecipitation of CT47 FLAG-HA was carried out according to the FLAG® HA Tandem Affinity Purification Kit. The purified protein complexes were eluted with sodium dodecyl sulfate (SDS) sample buffer and heated at 95 °C for 5 min. The samples were then run on a Bolt 4-12% NuPAGE Bis-Tris gel and stained using the ProteoSilver™ Plus Silver Stain Kit. The gel was cut horizontally according to molecular weight, and each gel strip was transferred to a 1.5-ml Eppendorf tube for further processing using an established method [55]. Liquid chromatography-tandem mass spectrometry (LC-MS/MS) analyses were performed at Shanghai Applied Protein Technology.

### RNA-seq sample collection and RNA-seq

For mice, testes were collected from control or *CT47*-BAC mice at P10 and P14. Samples from three mice in each group were pooled to perform RNA-seq. RNA-seq experiments were performed by Novogene Bioinformatics Institute (Beijing, China). Differential expression analysis was performed by DESeq2 with a threshold false discovery rate (FDR) < 0.05 with a 2-fold change. A volcano plot of differentially expressed genes was generated using the R program.

### RNA mutation analysis

High-throughput mRNA sequencing was carried out using the Illumina Novaseq 6000. FastQC (v0.11.8) and Trimmomatic (v.0.33) were used for quality control. Qualified reads were mapped to the reference genome (Ensemble GRCh38) using RSEM (v.1.3.0) and Bowtie2 (v.2.2.6). Then, SNVs were identified from the aligned sequence datasets using Picard tools to sort and mark duplicates and GATK (v.4.1.4.1) with SplitNCigarReads and HaplotypeCaller. To identify variants with high confidence, we filtered at least five SNVs that were within a window of 35 bases and retained variants with a base-quality score >25, a mapping quality score >20, Fisher strand values <30.0, qual by depth values >2.0 and a sequencing depth >20.

### Pathway analysis

GO and pathway analyses were performed using DAVID or Metascape. The *p* value indicates the importance of the path related to the condition (the recommended critical *p* value was 0.05).

### Cell transfection and treatment

HEK293T and COS7 cells were transfected with the indicated plasmids using Lipofectamine 2000 according to the manufacturer’s instructions. For the protein stability assay, cycloheximide (CHX) and MG132 were used. At 24 h posttransfection, cells were treated with the protein biosynthesis inhibitor CHX at a concentration of 10 μg/ml and then incubated for the indicated times. Total cell lysates were analyzed by Western blotting.

### Immunofluorescence, Immunohistochemistry, and histological analysis

At P10, P14, P20, P30, and adulthood, the mice were anesthetized and sacrificed. Testis tissues were removed and immersion fixed in Bouin’s solution or 4% paraformaldehyde (PFA) for up to 24 h. After tissue processing and paraffin embedding, sections of 3–5 μm were cut. To perform immunohistochemistry, sections were deparaffinized, immersed in 10 mM citrate buffer pH 6.0 or 1 mM ethylenediaminetetraacetic acid (EDTA) buffer pH 9.0, for 90 s at 120 °C in pressure cookers, followed by blocking with 5% normal donkey serum before overnight incubation with primary antibodies at 4 °C. Appropriate secondary antibodies conjugated with fluorophores were used the following day to complete the staining, and diamidino-2-phenylindole (DAPI) was used as a nuclear counterstain [56].

At each time point, the mice were anesthetized and sacrificed. Testis tissues were removed and fixed in Bouin’s solution for up to 12 h, stored in 70% ethanol, and embedded in paraffin. Sections of 3-μm thickness were cut and mounted on glass slides. After deparaffinization, sections were processed for hematoxylin and eosin (H&E) staining.

### TUNEL assay

Testis slides were processed with the ApopTag Fluorescein in Situ Apoptosis Detection Kit (Millipore) according to the manufacturer’s instructions.

### Mouse testis testosterone measurement

P14 WT and *CT47*-BAC testes were collected and stored at –80 °C. Testis testosterone was extracted as described previously [57]. Each frozen tissue sample was transferred to a 1.5-ml Eppendorf tube containing 500 µl buffer (0.5% BSA [wt/vol, 5 mM EDTA in PBS [pH 7.4]) and homogenized on ice for 60 s using a high-throughput homogenizer at 70 Hz. Homogenates were centrifuged at 3000 rpm for 10 min at 4 °C to settle the insoluble debris, and supernatants were transferred to fresh 1.5-ml Eppendorf tubes for analysis. Testis testosterone was measured by a testosterone enzyme-linked immunosorbent assay kit.

### Flow cytometric analysis of testicular cell populations

Tissue dissociation was carried out using previously described methods [58]. Briefly, 5 ml digestion medium per sample with 1 mg/ml collagenase and 1 mg/ml DNase I in DMEM/F12 was prepared. Testes were collected in ice-cold phosphate-buffered saline (PBS), and the tunica albuginea was removed carefully. The testicular tissue was minced in 1 ml of the digestion medium until all seminiferous tubule pieces were ≤1 mm long, and 4 ml digestion medium was added to each sample for 15 min incubation at 37 °C under slow continuous rotation. After centrifugation at 400 × *g* for 10 min at room temperature, the obtained pellet was washed twice with 5 ml PBS and resuspended in 5 ml 0.25% trypsin for 20 min at 37 °C, with careful shaking every 5 min. The cell suspension was filtered using a 40-μm pore size filter to achieve a single-cell suspension. The pellet obtained after centrifugation at 400 × *g* for 10 min at 4 °C was then suspended and washed twice with 5 ml PBS. Then, cell pellets were fixed in 75% ethanol for 4 h at 4 °C and stained with PI (50 μg/ml) in the presence of RNase A (50 μg/ml). Samples of 12 × 10^4^ cells/tube were analyzed using a FACS Area flow cytometer (Becton Dickinson and Company, Franklin Lakes, NJ, USA). Debris and doublets were excluded based on the pulse width vs. height ratio; only single cells were analyzed [59].

### Subcutaneous injection of testosterone

Testosterone propionate powder was dissolved in corn oil to a dose concentration of 0.5 mg/ml and stored at –80 °C. The mice were injected subcutaneously with concentrations of 50 ng/g/day and 5000 ng/g/day.

### Quantification and statistical analysis

The unpaired two-tailed Student’s *t* test was performed to identify significant differences between the two groups based on a *p* value < 0.05. GraphPad Prism 8 (for Windows) and Excel were used to perform the statistical analysis.

## Supplementary information

Supplementary information

Figure S1

Figure S2

Figure S3

Figure S4

Figure S5

Figure S6

Figure S7

Figure S8

Figure S9

Figure S10

Table S1

Table S2

Table S3

original western data

Authorship confirmed

Ethics Statement of human specimens

Language proofs

animal protocal

## Data Availability

All raw RNA-seq data are available in our SRA submission (PRJNA728395 and PRJNA728660).

## References

[1] Stoeger T, Gerlach M, Morimoto RI, Nunes Amaral LA (2018). Large-scale investigation of the reasons why potentially important genes are ignored. PLoS Biol.

[2] Zhang YE, Long M (2014). New genes contribute to genetic and phenotypic novelties in human evolution. Curr Opin Genet Dev.

[3] Necsulea A, Kaessmann H (2014). Evolutionary dynamics of coding and non-coding transcriptomes. Nat Rev Genet.

[4] Khaitovich P, Hellmann I, Enard W, Nowick K, Leinweber M, Franz H (2005). Parallel patterns of evolution in the genomes and transcriptomes of humans and chimpanzees. Science.

[5] Xia B, Yan Y, Baron M, Wagner F, Barkley D, Chiodin M (2020). Widespread transcriptional scanning in the testis modulates gene evolution rates. Cell.

[6] Wyckoff GJ, Wang W, Wu CI (2000). Rapid evolution of male reproductive genes in the descent of man. Nature.

[7] Matzuk MM, Lamb DJ (2008). The biology of infertility: research advances and clinical challenges. Nat Med.

[8] Walker WH (2011). Testosterone signaling and the regulation of spermatogenesis. Spermatogenesis.

[9] Cole LA. *Biology of Life: Biochemistry, Physiology and Philosophy*. (Academic Press; 2016).

[10] Kaprara A, Huhtaniemi IT (2018). The hypothalamus-pituitary-gonad axis: tales of mice and men. Metab: Clin Exp.

[11] McLachlan RI, O’Donnell L, Meachem SJ, Stanton PG, de Kretser DM, Pratis K (2002). Identification of specific sites of hormonal regulation in spermatogenesis in rats, monkeys, and man. Recent Prog Horm Res.

[12] Ramaswamy S, Weinbauer GF (2014). Endocrine control of spermatogenesis: Role of FSH and LH/ testosterone. Spermatogenesis.

[13] Tsai MY, Yeh SD, Wang RS, Yeh S, Zhang C, Lin HY (2006). Differential effects of spermatogenesis and fertility in mice lacking androgen receptor in individual testis cells. Proc Natl Acad Sci USA.

[14] Paniagua R, Nistal M (1984). Morphological and histometric study of human spermatogonia from birth to the onset of puberty. J Anat.

[15] O’Hara L, Smith LB (2015). Androgen receptor roles in spermatogenesis and infertility. Best Pract Res Clin Endocrinol Metab.

[16] Han JD, Bertin N, Hao T, Goldberg DS, Berriz GF, Zhang LV (2004). Evidence for dynamically organized modularity in the yeast protein-protein interaction network. Nature.

[17] Stahl PD, Wainszelbaum MJ (2009). Human-specific genes may offer a unique window into human cell signaling. Sci Signal.

[18] Lee YCG, Ventura IM, Rice GR, Chen DY, Colmenares SU, Long M (2019). Rapid evolution of gained essential developmental functions of a young gene via interactions with other essential genes. Mol Biol Evol.

[19] Kellis M, Wold B, Snyder MP, Bernstein BE, Kundaje A, Marinov GK (2014). Defining functional DNA elements in the human genome. Proc Natl Acad Sci USA.

[20] Chen YT, Iseli C, Venditti CA, Old LJ, Simpson AJ, Jongeneel CV (2006). Identification of a new cancer/testis gene family, CT47, among expressed multicopy genes on the human X chromosome. Genes Chromosomes Cancer.

[21] Balog J, Miller D, Sanchez-Curtailles E, Carbo-Marques J, Block G, Potman M (2012). Epigenetic regulation of the X-chromosomal macrosatellite repeat encoding for the cancer/testis gene CT47. Eur J Hum Genet.

[22] Mersaoui SY, Yu Z, Coulombe Y, Karam M, Busatto FF, Masson JY (2019). Arginine methylation of the DDX5 helicase RGG/RG motif by PRMT5 regulates resolution of RNA:DNA hybrids. EMBO J.

[23] Blanc RS, Richard S (2017). Arginine methylation: the coming of age. Mol cell.

[24] Li Z, Yu J, Hosohama L, Nee K, Gkountela S, Chaudhari S (2015). The Sm protein methyltransferase PRMT 5 is not required for primordial germ cell specification in mice. EMBO J.

[25] Charrier C, Joshi K, Coutinho-Budd J, Kim JE, Lambert N, de Marchena J (2012). Inhibition of SRGAP2 function by its human-specific paralogs induces neoteny during spine maturation. Cell.

[26] Florio M, Albert M, Taverna E, Namba T, Brandl H, Lewitus E (2015). Human-specific gene ARHGAP11B promotes basal progenitor amplification and neocortex expansion. Science.

[27] Suzuki IK, Gacquer D, Van Heurck R, Kumar D, Wojno M, Bilheu A (2018). Human-specific NOTCH2NL genes expand cortical neurogenesis through delta/notch regulation. Cell.

[28] Xu Y, Toh KL, Jones CR, Shin JY, Fu YH, Ptacek LJ (2007). Modeling of a human circadian mutation yields insights into clock regulation by PER2. Cell.

[29] Meistrich ML, and Hess RA. *Spermatogenesis*. 299–307 (Springer, 2013). 10.1007/978-1-62703-038-0_27 22992924

[30] Hamer G, Novak I, Kouznetsova A, Höög C (2008). Disruption of pairing and synapsis of chromosomes causes stage-specific apoptosis of male meiotic cells. Theriogenology.

[31] Panigrahi SK, Manterola M, Wolgemuth DJ (2017). Meiotic failure in cyclin A1-deficient mouse spermatocytes triggers apoptosis through intrinsic and extrinsic signaling pathways and 14-3-3 proteins. PLoS One.

[32] Wang Y, Zhu T, Li Q, Liu C, Han F, Chen M (2015). Prmt5 is required for germ cell survival during spermatogenesis in mice. Sci Rep.

[33] Girardot M, Hirasawa R, Kacem S, Fritsch L, Pontis J, Kota SK (2014). PRMT5-mediated histone H4 arginine-3 symmetrical dimethylation marks chromatin at G + C-rich regions of the mouse genome. Nucleic Acids Res.

[34] Kim S, Gunesdogan U, Zylicz JJ, Hackett JA, Cougot D, Bao S (2014). PRMT5 protects genomic integrity during global DNA demethylation in primordial germ cells and preimplantation embryos. Mol Cell.

[35] da Cruz I, Rodríguez-Casuriaga R, Santiñaque FF, Farías J, Curti G, Capoano CA (2016). Transcriptome analysis of highly purified mouse spermatogenic cell populations: gene expression signatures switch from meiotic-to postmeiotic-related processes at pachytene stage. BMC Genomics.

[36] Gou LT, Kang JY, Dai P, Wang X, Li F, Zhao S (2017). Ubiquitination-deficient mutations in human piwi cause male infertility by impairing histone-to-protamine exchange during spermiogenesis. Cell.

[37] Yang F, Lan Y, Pandey RR, Homolka D, Berger SL, Pillai RS (2020). TEX15 associates with MILI and silences transposable elements in male germ cells. Genes Dev.

[38] Dong J, Wang X, Cao C, Wen Y, Sakashita A, Chen S (2019). UHRF1 suppresses retrotransposons and cooperates with PRMT5 and PIWI proteins in male germ cells. Nat Commun.

[39] Guo J, Nie X, Giebler M, Mlcochova H, Wang Y, Grow EJ (2020). The dynamic transcriptional cell atlas of testis development during human puberty. cell stem cell.

[40] Fernandez-Capetillo O, Mahadevaiah SK, Celeste A, Romanienko PJ, Camerini-Otero RD, Bonner WM (2003). H2AX is required for chromatin remodeling and inactivation of sex chromosomes in male mouse meiosis. Dev Cell.

[41] Yang F, De La Fuente R, Leu NA, Baumann C, McLaughlin KJ, Wang PJ (2006). Mouse SYCP2 is required for synaptonemal complex assembly and chromosomal synapsis during male meiosis. J Cell Biol.

[42] Zhou C, Sun Y, Yan R, Liu Y, Zuo E, Gu C (2019). Off-target RNA mutation induced by DNA base editing and its elimination by mutagenesis. Nature.

[43] Easley CAT, Phillips BT, McGuire MM, Barringer JM, Valli H, Hermann BP (2012). Direct differentiation of human pluripotent stem cells into haploid spermatogenic cells. Cell Rep..

[44] Wistuba J, Mittag J, Luetjens CM, Cooper TG, Yeung CH, Nieschlag E (2007). Male congenital hypothyroid Pax8−/− mice are infertile despite adequate treatment with thyroid hormone. J Endocrinol.

[45] Wu X, Arumugam R, Zhang N, Lee MM (2010). Androgen profiles during pubertal Leydig cell development in mice. Reproduction.

[46] Hochberg Z, Belsky J (2013). Evo-devo of human adolescence: beyond disease models of early puberty. BMC Med.

[47] Smith LB, Walker WH (2014). The regulation of spermatogenesis by androgens. Semin Cell Dev Biol.

[48] Cooke HJ, Saunders PT (2002). Mouse models of male infertility. Nat Rev Genet.

[49] de Rooij DG (2001). Proliferation and differentiation of spermatogonial stem cells. Reproduction.

[50] Fedoriw A, Rajapurkar SR, O’Brien S, Gerhart SV, Mitchell LH, Adams ND (2019). Anti-tumor activity of the type I PRMT inhibitor, GSK3368715, synergizes with PRMT5 inhibition through MTAP loss. Cancer Cell.

[51] Deng X, Shao G, Zhang HT, Li C, Zhang D, Cheng L (2017). Protein arginine methyltransferase 5 functions as an epigenetic activator of the androgen receptor to promote prostate cancer cell growth. Oncogene.

[52] Kim H, Ronai ZA (2020). PRMT5 function and targeting in cancer. Cell Stress.

[53] Wu DD, Zhang YP (2013). Evolution and function of de novo originated genes. Mol Phylogenet Evol.

[54] Shen B, Zhang W, Zhang J, Zhou J, Wang J, Chen L (2014). Efficient genome modification by CRISPR-Cas9 nickase with minimal off-target effects. Nat methods.

[55] Shevchenko A, Tomas H, Havlis J, Olsen JV, Mann M (2006). In-gel digestion for mass spectrometric characterization of proteins and proteomes. Nat Protoc.

[56] Soh YQS, Mikedis MM, Kojima M, Godfrey AK, de Rooij DG, Page DC (2017). Meioc maintains an extended meiotic prophase I in mice. PLoS Genet.

[57] Handelsman DJ, Jimenez M, Singh GK, Spaliviero J, Desai R, Walters KA (2015). Measurement of testosterone by immunoassays and mass spectrometry in mouse serum, testicular, and ovarian extracts. Endocrinology.

[58] Rotgers E, Cisneros-Montalvo S, Jahnukainen K, Sandholm J, Toppari J, Nurmio M (2015). A detailed protocol for a rapid analysis of testicular cell populations using flow cytometry. Andrology.

[59] Ferreiro ME, Amarilla MS, Glienke L, Mendez CS, Gonzalez C, Jacobo PV (2019). The inflammatory mediators TNFalpha and nitric oxide arrest spermatogonia GC-1 cell cycle. Reprod Biol.

