# Supplementary material for: Human-specific gene CT47 blocks PRMT5 degradation to lead to meiosis arrest
Source: Cell Death Discov. 2022 Aug 2;8:345. doi: 10.1038/s41420-022-01139-6 (PMC9345867; doi:10.1038/s41420-022-01139-6)
Supplement: Supplementary file 1 — Supplementary information [file 41420_2022_1139_MOESM1_ESM.docx]

**Human-specific gene CT47 blocks PRMT5 degradation to lead to meiosis arrest**

Chao Li^1,9^, Yuming Feng^2,9^, Zhenxin Fu^1^, Junjie Deng^1^, Yue Gu^1^, Hanben Wang^3^, Xin Wu^3^, Zhengyun Huang^1^, Yichen Zhu^1^, Zhiwei Liu^1^, Moli Huang^1^, Tao Wang^1^, Shijun Hu^4^, Bing Yao^2^, Yizhun Zeng^1^, Chengji J. Zhou^5^, Steve D. M. Brown^6^, Yi Liu^7^, Antonio Vidal-Puig^8^, Yingying Dong^1*^, Ying Xu^1,10*^

**Supplementary information**

Supplementary legends:

Figure S1

Figure S2

Figure S3

Figure S4

Figure S5

Figure S6

Figure S7

Figure S8

Figure S9

Figure S10

Table S1

Table S2

Table S3

**Fig. S1 *CT47* produces multiple copies in the human genome specifically.**

**(A)** The alignment of amino acid sequence of *CT47A1*-*CT47A12*.

**(B)** *CT47* gene phylogenetic tree was constructed by the Ensemble database. Multiple *CT47* copies yielded (*CT47A1*-*CT47A12*) in the human branch when separated from chimpanzee.

**Fig. S2 CT47 Protein during Spermatogenesis of *CT47*-BAC Testis.**

**(A)** Immunofluorescence for CT47 (red) in the testes of P10, P14, P20 and P30 *CT47*-BAC testes. Nuclei were stained with DAPI. WT testis as a negative control. Scale bar = 25 μm.

**(B)** Histology of adult ovary by HE staining.

**Fig. S3 Immunofluorescence staining of γH2AX and SYCP3 at P30 WT and *CT47*-BAC testes.**

**(A)** Immunofluorescence staining of γH2AX at P30 WT and *CT47*-BAC testes. Scale Bar = 20 μm. L, leptotene. P, pachytene.

**(B)** Co**-**immunofluorescence of SYCP3 and Lectin PNA at P30 WT and *CT47*-BAC testes. Scale Bar = 20 μm.

**Fig. S4 Meiotic Maturate Arrest during Spermatogenesis of *CT47*-BAC Mice.**

**(A)** Chromosomal spread assay in WT and *CT47*-BAC spermatocytes. Double immunofluorescence staining for anti SYCP3 (red) and anti γ-H2AX (green). Nuclei were stained with DAPI. Scale bar = 5 μm.

**(B)** Immunofluorescence staining for γ-H2AX in sorted 4C spermatocytes from P14 WT and *CT47*-BAC mice. Scale bar = 40 μm.

**(C)** Representative results showing immunofluorescence staining for ZBTB16 in testes from P14 WT and *CT47*-BAC mice. DNA stained by DAPI. Scale bar = 12 μm.

**(D)** Numbers of ZBTB16(+) and ZBTB16(-) cells per tube in testes from P14 WT and *CT47*-BAC mice. The results shown are the mean values ± SD. n = 4 for each group.

**Fig. S5 The proliferation of germ cells.**

**(A)** Immunohistochemistry for PHH3 (phosphorylated histone H3, brown) in P10, P14, P20 and P30 of WT and *CT47*-BAC testes, with hematoxylin counterstaining to enable identification of germ cell types by nuclear morphology.

**(B)** PHH3-positive cells at P10, P14, P20, and P30 from WT and *CT47*-BAC mice were scanned by TissueFAXS. Each section including all seminiferous tubules was scanned and positive signals were automatically counted. Then the proportion was calculated. The results shown are the mean ± SD, P10 WT(n=4), *CT47*-BAC(n=4); P14 WT(n=7), *CT47*-BAC(n=7); P20 WT(n=3), *CT47*-BAC(n=3); P30 WT(n=4), *CT47*-BAC(n=4). Scale bar = 100 μm. N.S. = no significant difference between the two groups.

**Fig. S6 The numbers of apoptotic cells in WT and *CT47*-BAC testes.**

**(A)** Immunofluorescence staining for TUNEL (red) in P10, P14, P20, and P30 WT and *CT47*-BAC testes. Scale bar = 100 µm.

**(B)** TUNEL-positive cell numbers per 100 μm^2^ testis tissue area measured in P10, P14, P20, and P30 WT and *CT47*-BAC testes. Positive signals were detected by Tissue FAXS. Each section including all seminiferous tubules was scanned and positive signals were automatically counted. The mean values ± SD are shown. Student’s t*-*test: P10 WT(n=5), *CT47*-BAC(n=4); P14 WT(n=6), *CT47*-BAC(n=6); P20 WT(n=5), *CT47*-BAC(n=8); P30 WT(n=6), *CT47*-BAC(n=6), **p* < 0.05, ****p* < 0.001, N.S. = no significance.

**Fig. S7 Double staining of γH2AX and PRMT5 at P30 WT and *CT47*-BAC testes.** Scale Bar = 20 μm.

**Fig. S8 Exogenous testosterone effect on LNCaP cells and CT47 BAC mice.**

**(A)** Measurement of testis testosterone concentrations in P14 day from WT and CT47-BAC mice administered by different concentrations of testosterone. Student’s t-test: n = 6 for each group, N.S. means *p* > 0.05, ****p*<0.001.

**(B)** Western blot analysis of PRMT5 and CT47 from cytoplasmic and nuclear fractions of LNCaP cells without or with testosterone treatment.

**(C)** Quantification of PRMT5 and CT47 levels in each group. The mean values ± SD are shown. Student’s t-test: n = 3, **p* < 0.05.

**Fig. S9 Generation of *CT47*^-/-^ hESCs using CRISPR/Cas9.**

**(A)** Sanger sequencing of *CT47*^-/-^ hESCs. The 26 nt bases were deleted in the open reading frame of CT47.

**(B)** *CT47* expression levels in H1 and *CT47*^-/-^ hEBs differentiated from hESCs were analyzed by real time (RT)-qPCR. Error bar, mean ± error. Two technical replicates were performed.

**(C)** Upper: The shared RNA SNV sites between H1 and *CT47*^-/-^ hESCs. n = 2 for each sample. Bottom: Off-target identified among the predicted off-target sites.

**(D)** Karyotypic analysis of H1 and *CT47*^-/-^ hESCs.

**(E)** Characterization of representative H1 and *CT47*^-/-^ hESCs by alkaline phosphatase (ALP) staining. Scale bar = 20 μm.

**(F)** The expression of the stem cell markers *OCT4*, *NANOG*, and *SOX2* in H1 and *CT47*^-/-^ hESCs.

**Fig. S10 Immunofluorescence staining using the germ cell marker VASA and postmeiotic spermatid marker acrosin.**

**(A)** Immunofluorescence staining of VASA in H1 and *CT47*^-/-^ SSCs with or without testosterone treatment. Scale bar = 50 μm.

**(B)** Quantitative analysis of VASA^+^ and VASA^-^ cells in indicated groups.

**(C)** Lectin PNA (green) staining on H1 and CT47^-/-^ SSCs with or without testosterone treatment. Scale bar = 20 μm.

**Table S1. Information for five Nonobstructive azoospermia (NOA 1 - 5) and obstructive azoospermia (OA 1 - 5).**

**Table S2. The list of differentially expressed genes between *CT47*-BAC and WT mice at P14.**

**Table S3. Resources Table of experimental material.**
