# Supplementary figures and images for: Human-specific gene CT47 blocks PRMT5 degradation to lead to meiosis arrest
Source: Cell Death Discov. 2022 Aug 2;8:345. doi: 10.1038/s41420-022-01139-6 (PMC9345867; doi:10.1038/s41420-022-01139-6)

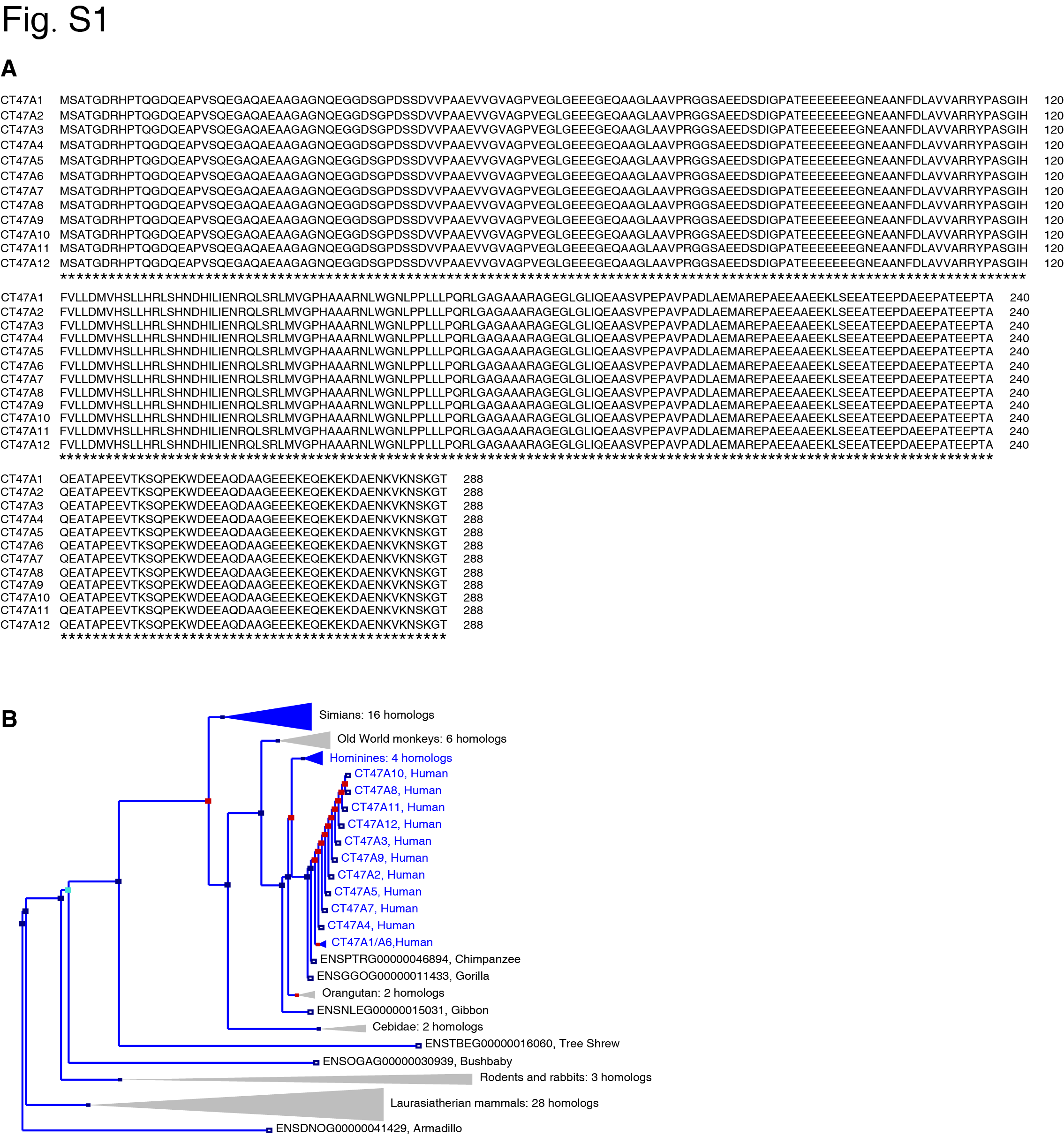

Supplement: Supplementary file 2 — Figure S1 [file 41420_2022_1139_MOESM2_ESM.tif]

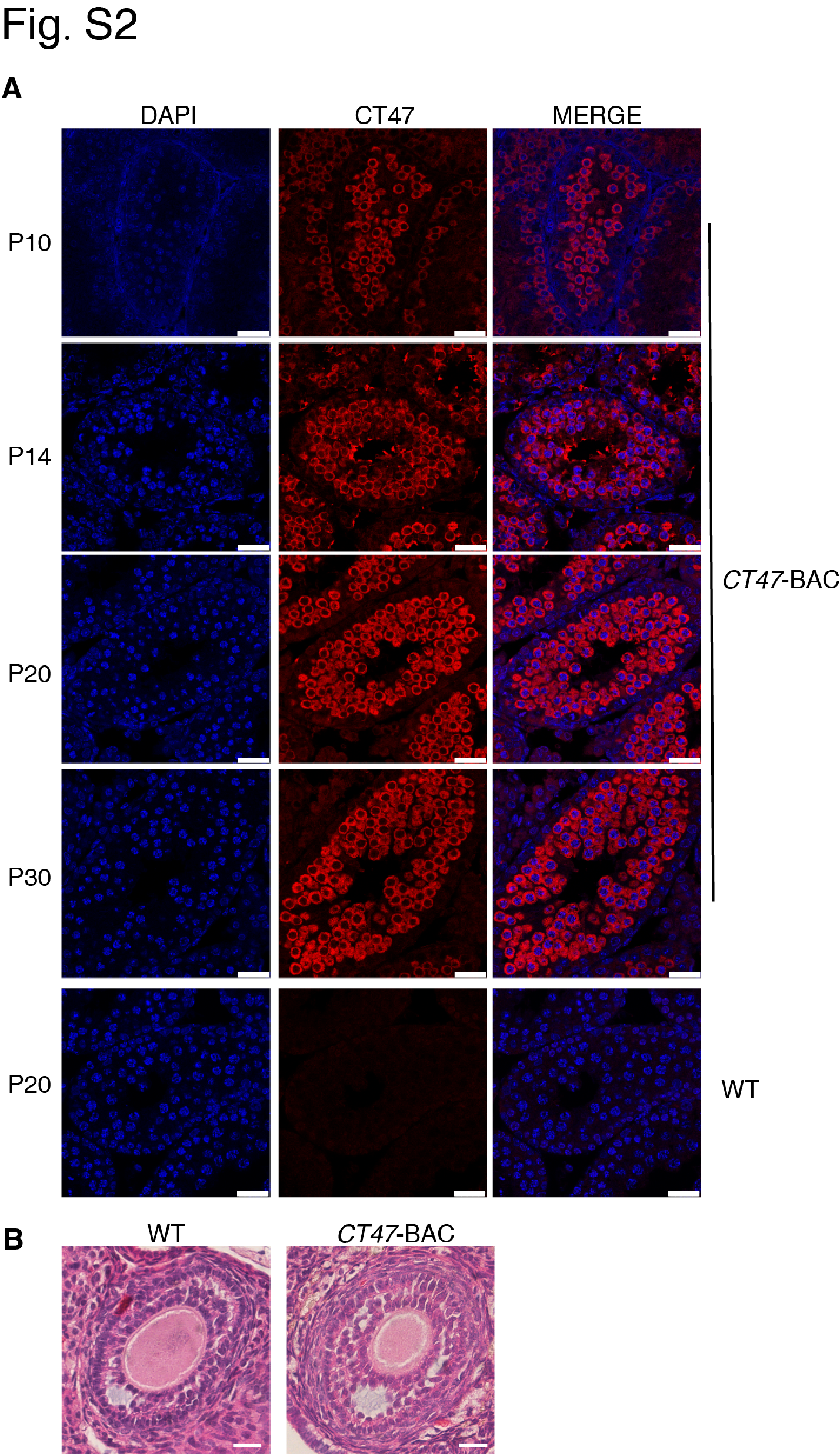

Supplement: Supplementary file 3 — Figure S2 [file 41420_2022_1139_MOESM3_ESM.tif]

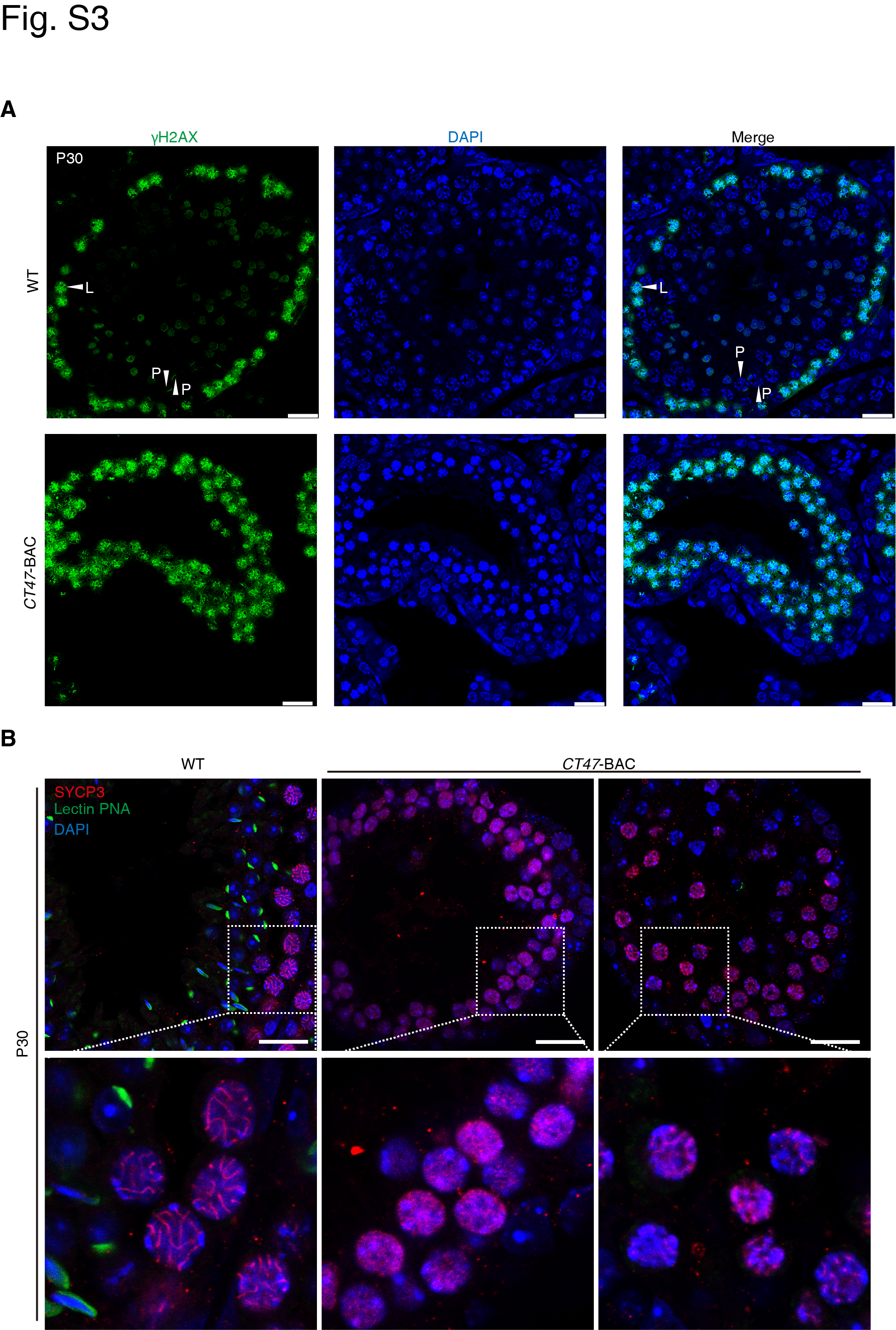

Supplement: Supplementary file 4 — Figure S3 [file 41420_2022_1139_MOESM4_ESM.tif]

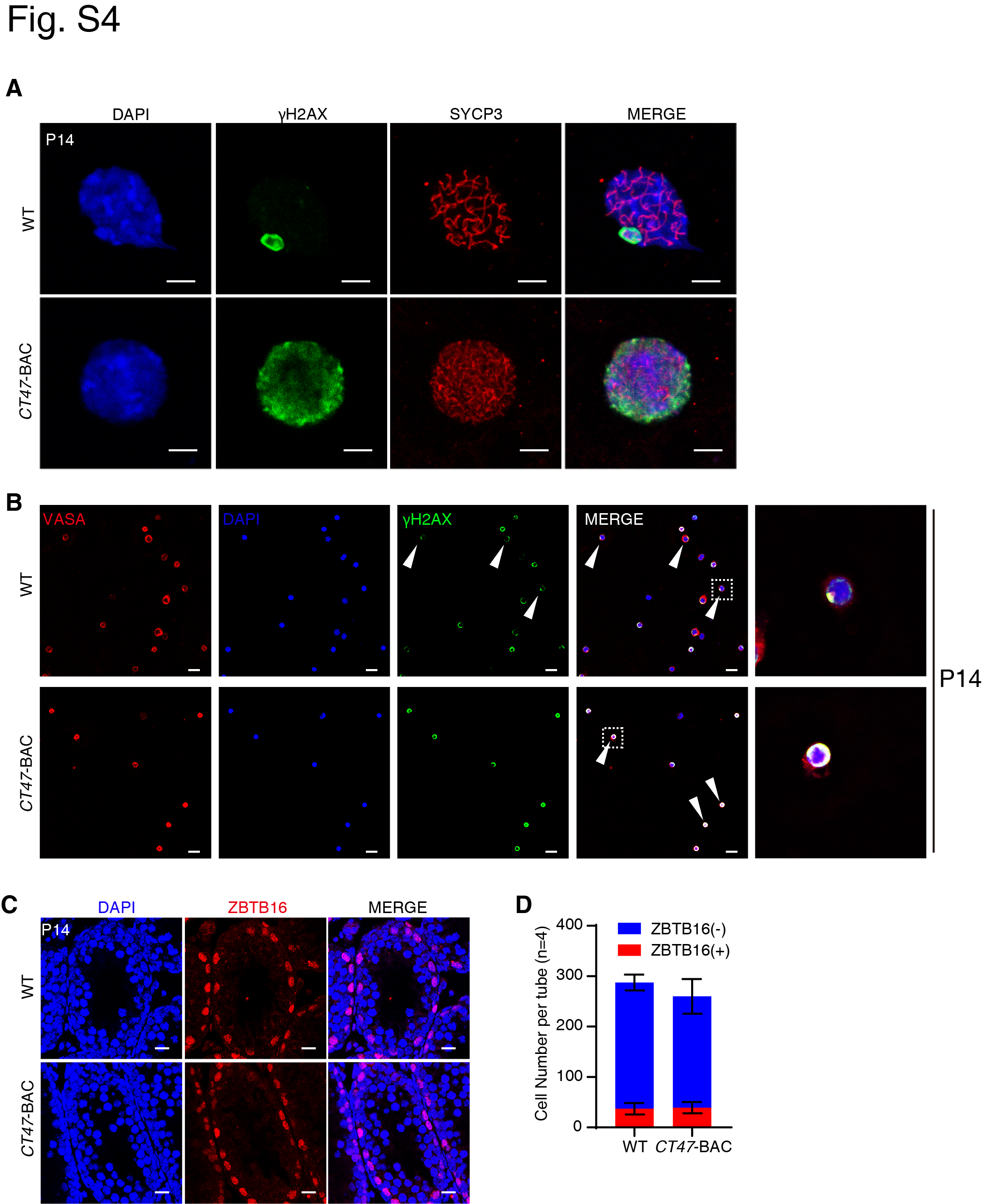

Supplement: Supplementary file 5 — Figure S4 [file 41420_2022_1139_MOESM5_ESM.tif]

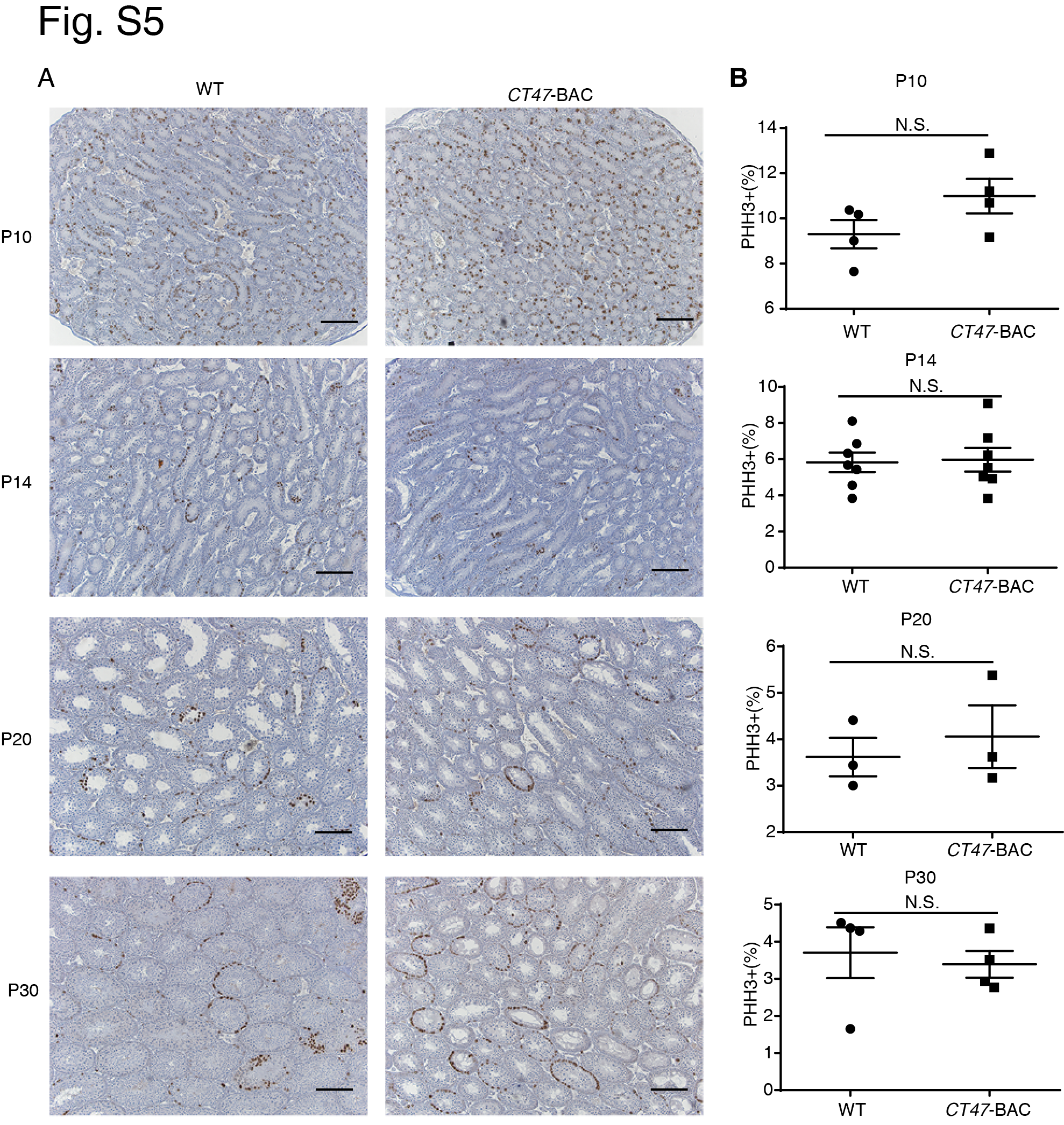

Supplement: Supplementary file 6 — Figure S5 [file 41420_2022_1139_MOESM6_ESM.tif]

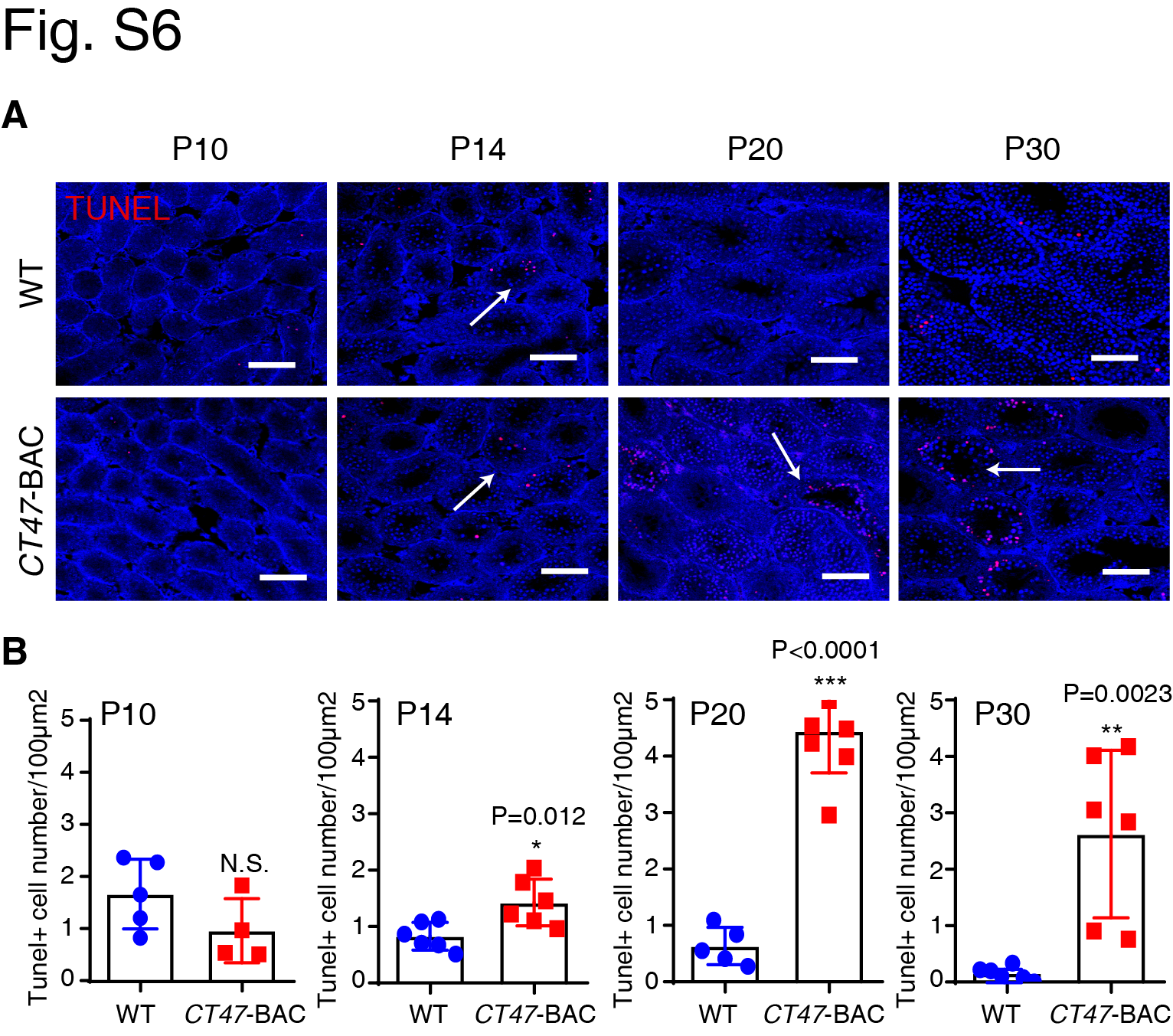

Supplement: Supplementary file 7 — Figure S6 [file 41420_2022_1139_MOESM7_ESM.tif]

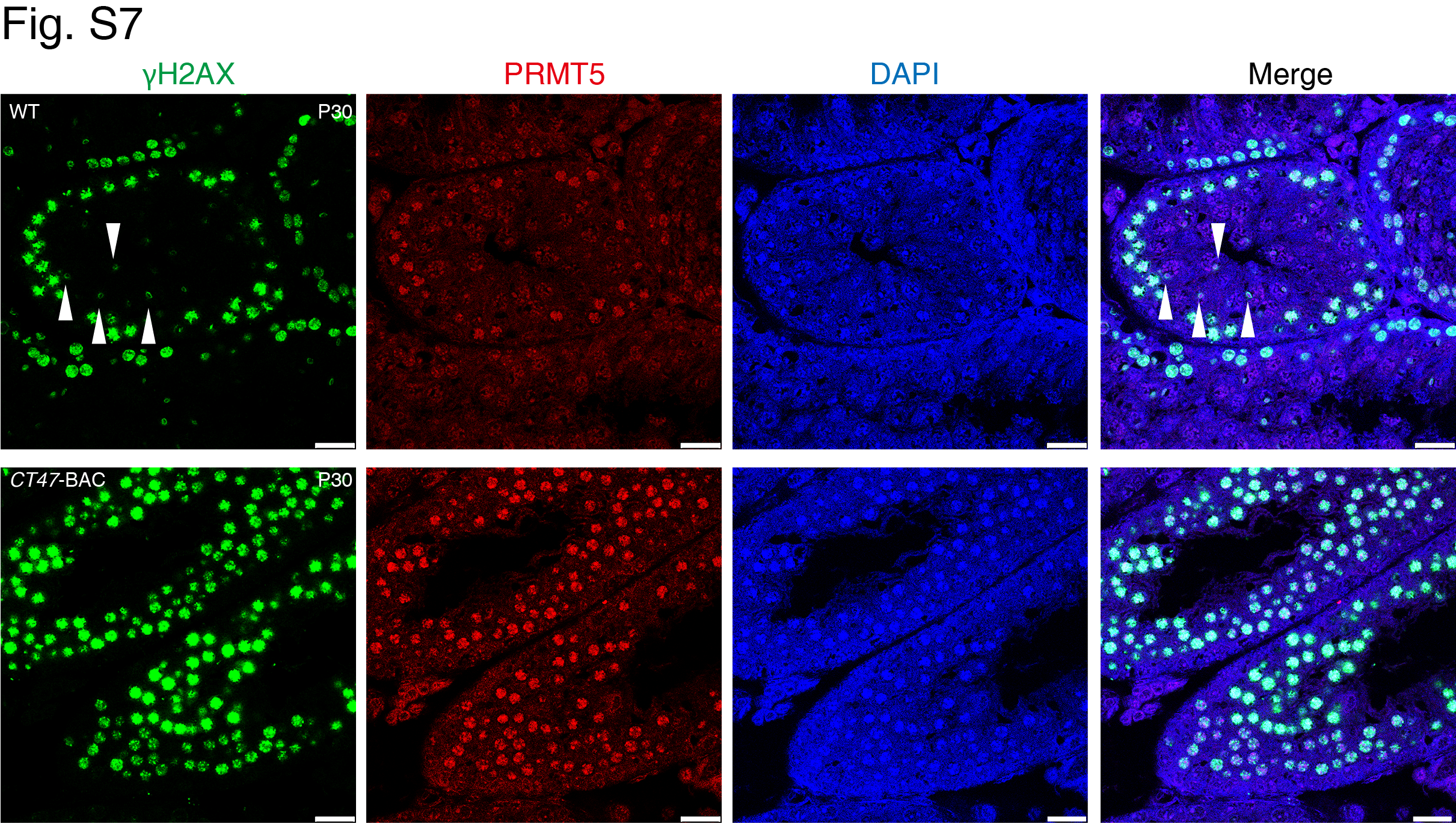

Supplement: Supplementary file 8 — Figure S7 [file 41420_2022_1139_MOESM8_ESM.tif]

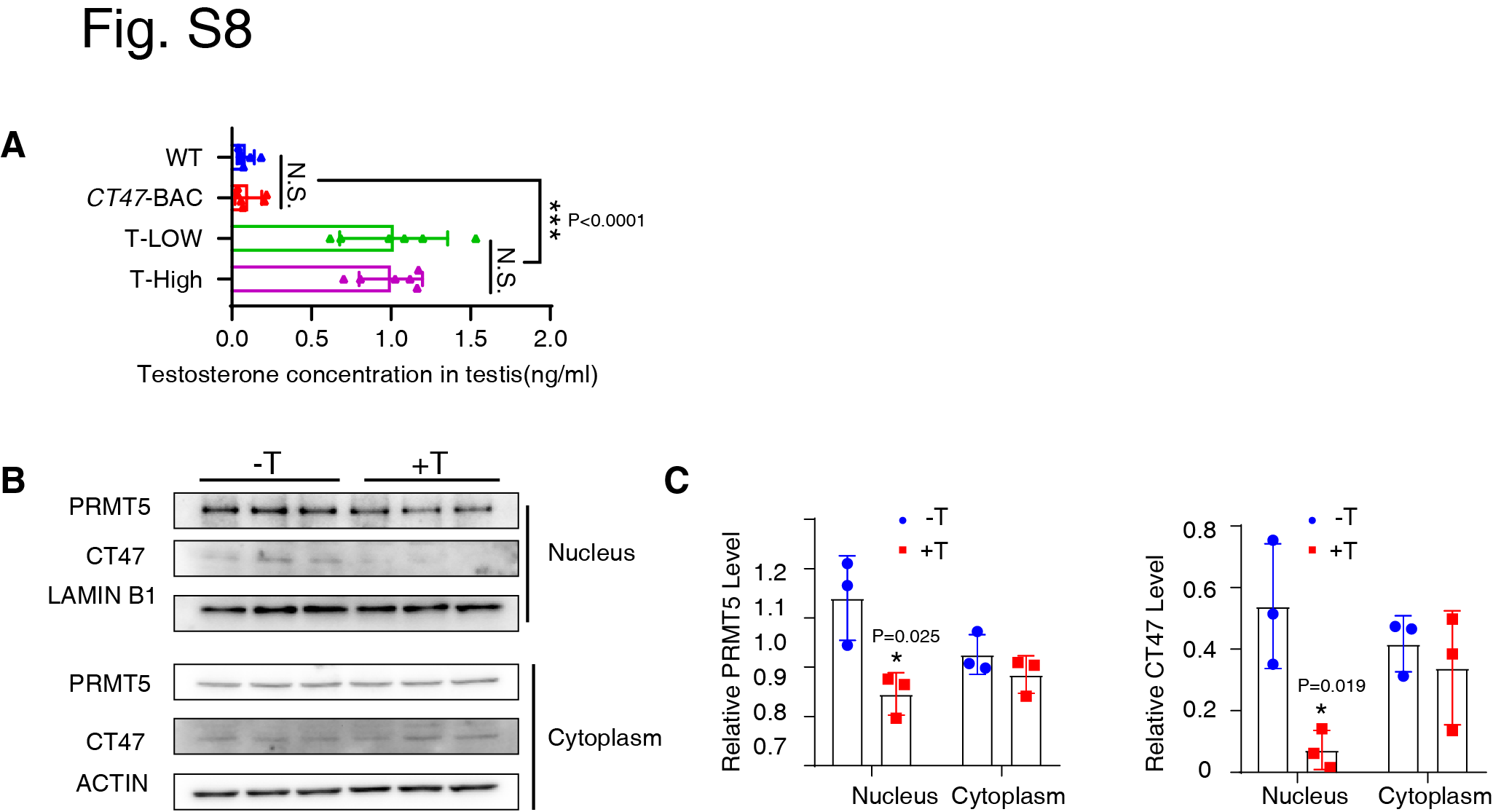

Supplement: Supplementary file 9 — Figure S8 [file 41420_2022_1139_MOESM9_ESM.tif]

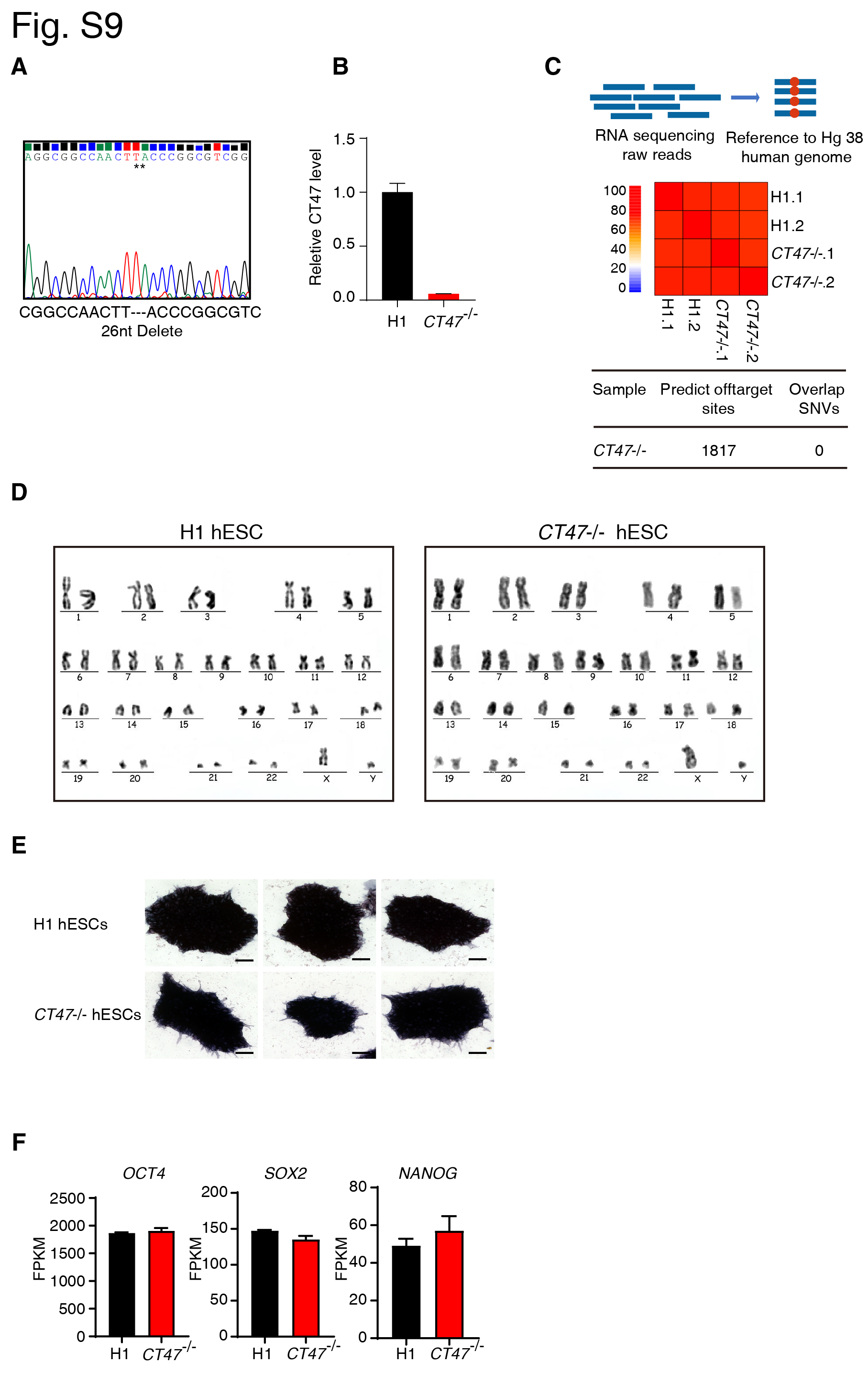

Supplement: Supplementary file 10 — Figure S9 [file 41420_2022_1139_MOESM10_ESM.tif]

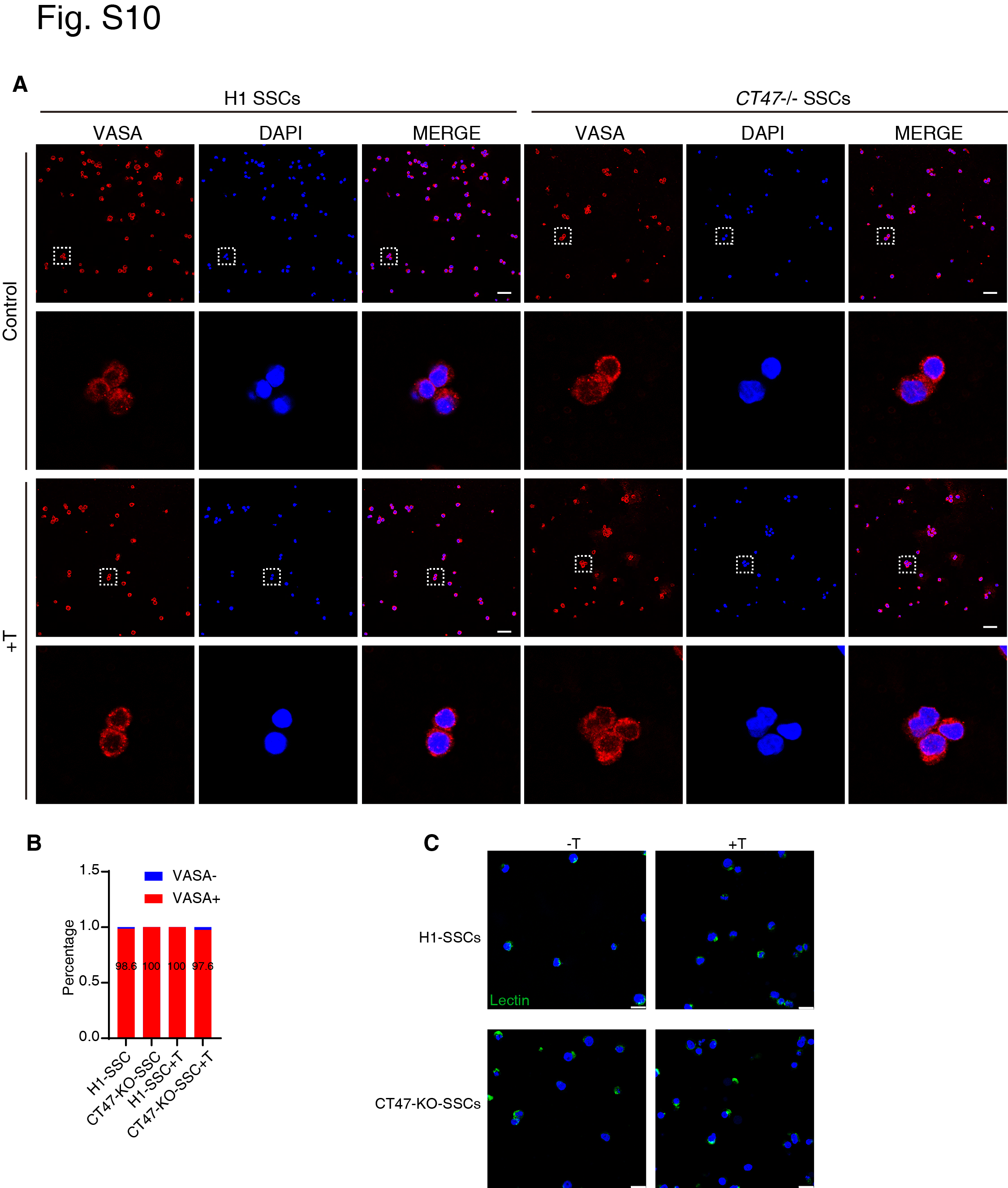

Supplement: Supplementary file 11 — Figure S10 [file 41420_2022_1139_MOESM11_ESM.tif]

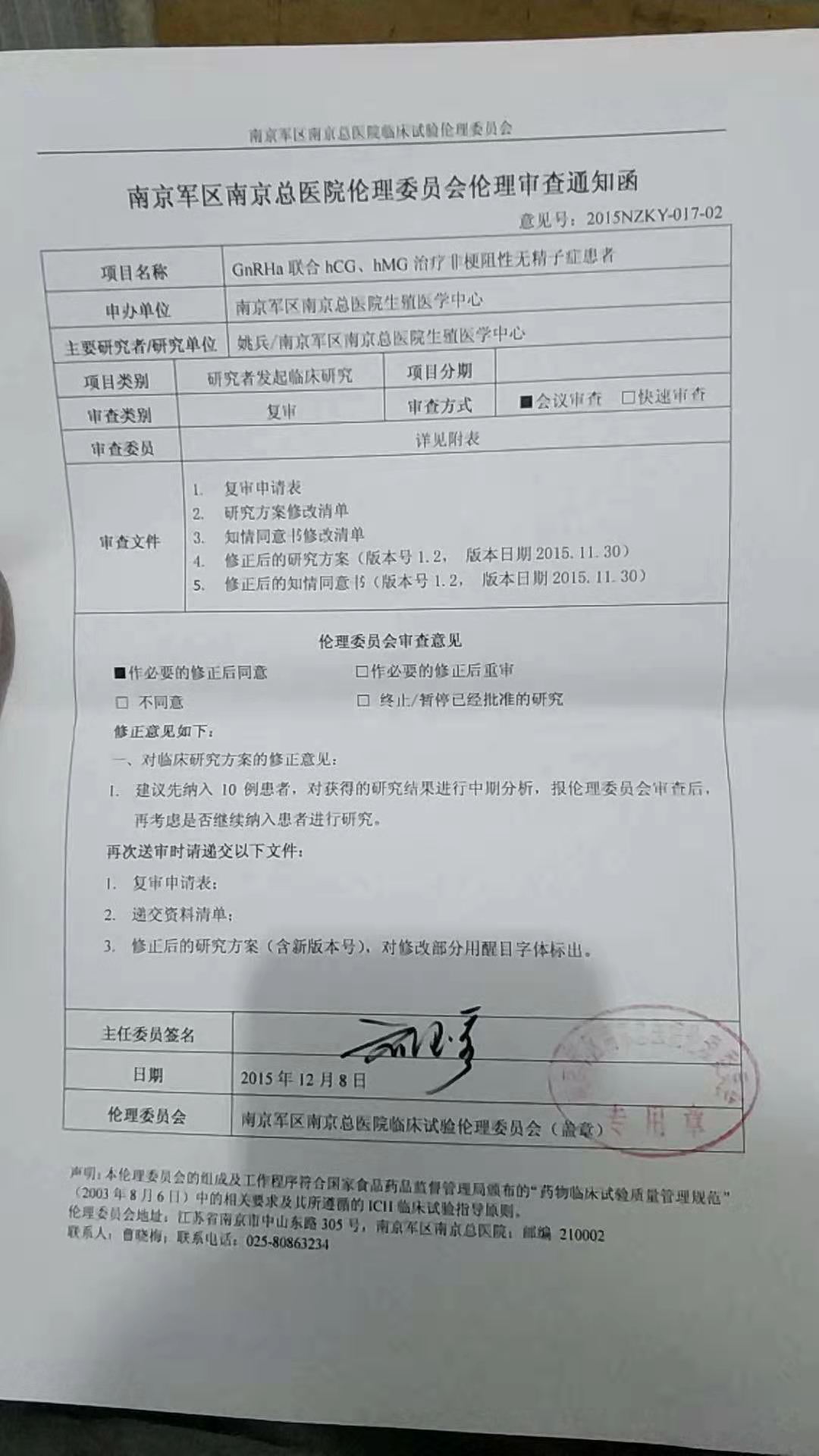

Supplement: Supplementary file 17 — Ethics Statement of human specimens [file 41420_2022_1139_MOESM17_ESM.jpg]
