# Supplementary material for: Human-specific gene CT47 blocks PRMT5 degradation to lead to meiosis arrest
Source: Cell Death Discov. 2022 Aug 2;8:345. doi: 10.1038/s41420-022-01139-6 (PMC9345867; doi:10.1038/s41420-022-01139-6)
Supplement: Supplementary file 12 — Table S1 [file 41420_2022_1139_MOESM12_ESM.doc]

**Table S1. Information for five Nonobstructive azoospermia (NOA 1 - 5) and obstructive azoospermia (OA 1 - 5).**

Obstructive Azoospermia Group (Normal sperm)

| Pacients | OA1 | OA2 | OA3 | OA4 | OA5 |
| --- | --- | --- | --- | --- | --- |
| Sex | Male | Male | Male | Male | Male |
| DOB (Date of Birth) | 1985-09-01 | 1965-12-08 | 1989-09-05 | 1994-06-26 | 1993-06-19 |
| Age | 34 | 53 | 29 | 25 | 26 |
| Height (cm) | 175 | 165 | 168 | 176 | 161 |
| Weight (kg) | 66 | 65 | 60 | 75 | 54 |
| T (nmol/L) | 6.46 | 13.85 | 15.91 | 26.51 | 11.24 |
| PRL (mIU/L) |  | 225.5 | 246.09 | 92.75 | 523.11 |
| E2 (pmol/L) |  | 131 | 130 | 69 | 100 |
| LH (IU/L) | 3.86 | 4.97 | 3.43 | 3.3 | 5.61 |
| FSH (IU/L) | 6.46 | 9.04 | 3.87 | 5.69 | 5.11 |
| INHB (ng/L) |  | 91.9 | 131 | 132 | 132 |

Nonobstructive Azoospermia Group (Azoospermia)

| Pacients | NOA1 | NOA2 | NOA3 | NOA4 | NOA5 |
| --- | --- | --- | --- | --- | --- |
| Sex | Male | Male | Male | Male | Male |
| DOB (Date of Birth) | 1987-06-18 | 1988-01-22 | 1989-03-08 | 1989-11-23 | 1990-06-19 |
| Age | 32 | 31 | 30 | 30 | 29 |
| Height (cm) | 168 | 165 | 170 | 178 | 178 |
| Weight (kg) | 70 | 68 | 80 | 70 | 57.5 |
| T (nmol/L) | 30.14 | 16.91 | 41.19 | 13.87 | 17.89 |
| PRL (mIU/L) | 146.13 | 351.59 | 666.04 | 315.63 | 123.07 |
| E2 (pmol/L) | 65 | 68 | 62 | 159 | 221 |
| LH (IU/L) | 12.3 | 3.86 | 16.72 | 9.86 | 5.59 |
| FSH (IU/L) | 26.57 | 5.26 | 15.4 | 17.89 | 3.71 |
| INHB (ng/L) | 23.1 | 132 | 16.6 | 15.7 | 80 |
