# Supplementary material for: Human-specific gene CT47 blocks PRMT5 degradation to lead to meiosis arrest
Source: Cell Death Discov. 2022 Aug 2;8:345. doi: 10.1038/s41420-022-01139-6 (PMC9345867; doi:10.1038/s41420-022-01139-6)
Supplement: Supplementary file 13 — Table S2 [file 41420_2022_1139_MOESM13_ESM.docx]

**Table S2. The list of differentially expressed genes between *CT47*-BAC and WT mice at P14.**

| **Gene_Symbol** | **log2FoldChange** | **padj** |
| --- | --- | --- |
| 1110017D15Rik | -2.324641248 | 0.014695753 |
| 1700001C02Rik | -5.743003313 | 0.004689481 |
| 1700001G11Rik | -1.527247371 | 0.0066279 |
| 1700001J11Rik | -3.559978034 | 0.002436707 |
| 1700003E16Rik | -1.679513865 | 8.27E-09 |
| 1700003G18Rik | -1.405333779 | 0.022861135 |
| 1700003H04Rik | -5.432915683 | 0.01187515 |
| 1700003I22Rik | -5.172829269 | 0.035669416 |
| 1700003M02Rik | -2.95248345 | 2.19E-11 |
| 1700006A11Rik | -3.728518112 | 8.42E-38 |
| 1700007G11Rik | -2.046644072 | 0.001190849 |
| 1700008K24Rik | -2.321704635 | 1.03E-05 |
| 1700009C05Rik | -3.116514936 | 0.001427811 |
| 1700010B08Rik | -2.464889438 | 4.72E-06 |
| 1700010I14Rik | -2.389181698 | 1.22E-19 |
| 1700011E24Rik | -5.612003914 | 1.04E-10 |
| 1700011I03Rik | -3.089365858 | 2.08E-10 |
| 1700012B09Rik | -3.011766709 | 2.90E-13 |
| 1700016K19Rik | -1.626242094 | 7.50E-07 |
| 1700017D01Rik | -6.045250903 | 2.22E-06 |
| 1700018F24Rik | -6.967540431 | 1.72E-06 |
| 1700018L02Rik | -1.365768499 | 0.007249026 |
| 1700019A02Rik | -1.731950607 | 4.27E-11 |
| 1700020N18Rik | -4.267816494 | 5.32E-19 |
| 1700021F07Rik | -2.253441989 | 0.025710943 |
| 1700026D08Rik | -1.322726581 | 0.028575031 |
| 1700026D11Rik | -4.090675579 | 0.03300039 |
| 1700028J19Rik | -1.923450361 | 1.79E-07 |
| 1700028P14Rik | -2.782778436 | 0.049691918 |
| 1700029F12Rik | -7.860681633 | 1.69E-08 |
| 1700029M20Rik | -2.02068414 | 1.45E-45 |
| 1700029P11Rik | -1.855320938 | 2.52E-20 |
| 1700040L02Rik | -1.567659835 | 1.28E-15 |
| 1700042G15Rik | -4.008855377 | 7.68E-40 |
| 1700047E10Rik | -1.244717679 | 0.000529017 |
| 1700051A21Rik | -6.437899962 | 8.36E-05 |
| 1700074P13Rik | -5.260289396 | 7.93E-08 |
| 1700086L19Rik | -3.893338499 | 0.006787665 |
| 1700088E04Rik | -1.044895664 | 0.000435551 |
| 1700097N02Rik | -2.684605701 | 2.91E-09 |
| 1700100I10Rik | -2.653010182 | 6.19E-05 |
| 1700102P08Rik | -1.486473666 | 1.31E-07 |
| 1700108J01Rik | -4.788707804 | 5.59E-06 |
| 1700110I01Rik | -5.891616068 | 0.000270935 |
| 1700123L14Rik | -4.151751562 | 6.35E-08 |
| 1700129C05Rik | -5.14118818 | 0.00569101 |
| 4833427G06Rik | -2.160786167 | 0.000511594 |
| 4921506M07Rik | -1.691500918 | 0.011889721 |
| 4921524L21Rik | -6.12113506 | 0.001123756 |
| 4921525O09Rik | -4.770882705 | 5.22E-40 |
| 4921536K21Rik | -1.034821344 | 0.014238505 |
| 4922502D21Rik | -3.897080497 | 6.62E-46 |
| 4930430A15Rik | -7.475998086 | 7.82E-08 |
| 4930442J19Rik | -1.587767923 | 0.000833747 |
| 4930444F02Rik | -4.086558132 | 7.39E-41 |
| 4930444P10Rik | -2.437694896 | 1.11E-05 |
| 4930455B14Rik | -3.354777672 | 0.009127952 |
| 4930463O16Rik | -1.742028729 | 5.71E-10 |
| 4930465K10Rik | -2.819656052 | 1.09E-06 |
| 4930488L21Rik | -3.658150371 | 2.14E-05 |
| 4930503E14Rik | -1.607038725 | 0.002212721 |
| 4930504O13Rik | -1.442323221 | 0.00040115 |
| 4930525D18Rik | -6.871713794 | 2.28E-08 |
| 4930544G11Rik | -5.318709518 | 5.22E-08 |
| 4930558J18Rik | -1.268725186 | 5.30E-08 |
| 4930579G24Rik | -1.133875145 | 4.97E-16 |
| 4931408D14Rik | -3.620461148 | 2.72E-09 |
| 4931417E11Rik | -4.027443385 | 6.03E-06 |
| 4931423N10Rik | -2.485317029 | 0.039192657 |
| 4931428L18Rik | -1.984307442 | 1.83E-08 |
| 4931429I11Rik | -2.018589128 | 0.00046256 |
| 4932414N04Rik | -3.51951285 | 3.25E-13 |
| 4933402N22Rik | -7.052554423 | 3.76E-06 |
| 4933404O12Rik | -2.058971483 | 3.46E-36 |
| 4933406C10Rik | -1.469678815 | 8.38E-05 |
| 4933406F09Rik | -5.406521324 | 1.54E-08 |
| 4933408B17Rik | -1.534372123 | 5.13E-09 |
| 4933409G03Rik | -2.456843944 | 0.003921877 |
| 4933412E24Rik | -2.675863217 | 4.50E-10 |
| 4933415F23Rik | -2.333004806 | 1.50E-12 |
| 4933425L06Rik | -1.214305494 | 4.96E-05 |
| 4933431G14Rik | -2.611543091 | 0.013369368 |
| 4933433G15Rik | -1.138566827 | 0.0004387 |
| 4933440M02Rik | -3.090513658 | 6.96E-24 |
| 5330417C22Rik | -1.572471446 | 4.34E-08 |
| 6430531B16Rik | -1.481390308 | 1.26E-06 |
| 9230105E05Rik | -2.136247516 | 2.25E-06 |
| 9330102E08Rik | -1.235616954 | 1.28E-25 |
| AA467197 | -1.703457672 | 1.00E-06 |
| AI464131 | -1.428023067 | 3.48E-05 |
| AY702103 | -5.212128784 | 8.60E-11 |
| Abca14 | -6.248055783 | 0.000329026 |
| Abcc12 | -5.164355883 | 1.66E-28 |
| Abhd14a | -1.129622512 | 1.87E-08 |
| Abo | -1.343505529 | 0.012001823 |
| Acr | -2.999375602 | 1.79E-14 |
| Acrbp | -1.125038332 | 6.47E-08 |
| Actl7b | -4.235410244 | 8.68E-16 |
| Acyp1 | -1.15280613 | 5.54E-10 |
| Adad2 | -1.432680496 | 7.26E-34 |
| Adam18 | -6.550654545 | 5.82E-05 |
| Adam1b | -3.419757943 | 3.21E-11 |
| Adam2 | -2.76454622 | 3.04E-40 |
| Adam3 | -7.15529277 | 9.96E-07 |
| Adam32 | -4.076532609 | 1.91E-20 |
| Adam5 | -4.467569537 | 3.56E-48 |
| Adgb | -1.286262233 | 5.54E-05 |
| Agbl2 | -1.857690684 | 5.94E-37 |
| Ak7 | -1.5864721 | 1.06E-15 |
| Ak8 | -1.704411279 | 0.000667215 |
| Als2cr11 | -1.168174143 | 3.24E-09 |
| Als2cr12 | -2.553828073 | 7.18E-15 |
| Alx1 | -1.767720533 | 0.000955538 |
| Ankar | -4.226657121 | 1.53E-11 |
| Ankef1 | -1.568905615 | 2.78E-06 |
| Ankmy1 | -1.096560394 | 0.008173204 |
| Ankrd36 | -2.75066547 | 1.03E-22 |
| Aqp9 | -3.141410176 | 0.000185787 |
| Arhgap33os | -1.910820678 | 0.000103831 |
| Armc3 | -3.075804788 | 1.92E-24 |
| Armc4 | -3.933220157 | 6.87E-06 |
| Armt1 | -1.124846845 | 1.42E-13 |
| Asrgl1 | -1.237099425 | 5.96E-11 |
| Atp8b3 | -3.722515239 | 3.13E-69 |
| Aym1 | -3.311683903 | 0.008821014 |
| BC048644 | -1.835808522 | 1.15E-08 |
| BC049730 | -2.819357339 | 8.11E-17 |
| Bcl11a | -1.111020974 | 0.001609571 |
| Bpi | -3.002169878 | 0.014256911 |
| Bspry | -1.265779114 | 0.000469566 |
| Btbd16 | -3.472756589 | 2.16E-05 |
| Bzrap1 | -1.198295323 | 2.12E-05 |
| C2cd4b | -1.034870852 | 0.006265829 |
| C530008M17Rik | -1.043265443 | 4.18E-09 |
| CK137956 | -2.405224395 | 0.02143557 |
| Cage1 | -1.680778776 | 1.17E-14 |
| Capsl | -1.663309219 | 0.037061683 |
| Catsperb | -5.008373728 | 1.10E-09 |
| Catsperd | -1.407606863 | 0.001012977 |
| Catsperg1 | -1.409256807 | 1.09E-12 |
| Catsperg2 | -2.450352284 | 1.10E-30 |
| Ccdc108 | -3.148606147 | 1.33E-66 |
| Ccdc113 | -2.495045123 | 3.67E-13 |
| Ccdc13 | -1.786031602 | 1.61E-14 |
| Ccdc144b | -3.213893619 | 5.77E-05 |
| Ccdc146 | -1.853242577 | 7.15E-10 |
| Ccdc150 | -1.011458963 | 7.44E-05 |
| Ccdc151 | -1.249432904 | 6.66E-06 |
| Ccdc173 | -1.29023421 | 1.44E-07 |
| Ccdc175 | -3.51911171 | 6.07E-07 |
| Ccdc176 | -1.037358844 | 3.93E-06 |
| Ccdc180 | -2.040724827 | 1.05E-14 |
| Ccdc30 | -1.712975819 | 8.44E-07 |
| Ccdc38 | -1.937174622 | 3.50E-05 |
| Ccdc39 | -2.075759712 | 2.18E-28 |
| Ccdc60 | -2.586400842 | 2.47E-14 |
| Ccdc65 | -2.524078167 | 1.89E-16 |
| Ccdc74a | -1.515432941 | 0.000738054 |
| Ccdc87 | -1.428571541 | 4.29E-07 |
| Ccdc89 | -3.027420124 | 2.90E-17 |
| Ccdc96 | -1.074102198 | 3.84E-07 |
| Cct6b | -2.082989937 | 2.98E-09 |
| Cd55b | -3.8122697 | 0.013505578 |
| Cdc42ep3 | -1.530684404 | 3.26E-25 |
| Cdkl4 | -2.839836048 | 4.41E-10 |
| Cds1 | -1.009750878 | 3.16E-11 |
| Ceacam20 | -2.484872349 | 0.000869159 |
| Cep128 | -1.141102499 | 4.36E-14 |
| Cep83os | -1.111671883 | 2.35E-19 |
| Cers3 | -2.25426252 | 4.55E-28 |
| Cetn1 | -3.550235452 | 1.92E-14 |
| Cetn4 | -1.294857503 | 1.30E-10 |
| Cfap221 | -3.926372048 | 0.000126754 |
| Cfap43 | -1.802741565 | 1.36E-18 |
| Cfap44 | -1.944752252 | 0.000583847 |
| Cfap45 | -2.985415824 | 6.13E-10 |
| Cfap46 | -2.863567167 | 4.21E-47 |
| Cfap52 | -3.245287787 | 4.13E-17 |
| Cfap57 | -2.278432861 | 2.48E-19 |
| Cfap58 | -3.128425811 | 0.039751767 |
| Cfap69 | -1.145718967 | 1.29E-09 |
| Cfap70 | -3.364137154 | 1.83E-25 |
| Cfap74 | -2.04149041 | 4.73E-18 |
| ChkbCpt1b | -6.447670993 | 0.000185787 |
| Chst1 | -1.955371307 | 1.98E-24 |
| Clgn | -1.720159172 | 8.82E-55 |
| Clhc1 | -1.641930823 | 0.000864211 |
| Cmtm2a | -5.346355934 | 4.14E-16 |
| Cmtm2b | -7.567356578 | 5.56E-07 |
| Cntd1 | -1.326608901 | 1.65E-17 |
| Cox6b2 | -1.550632824 | 2.97E-17 |
| Cox7b2 | -1.168452567 | 5.42E-13 |
| Cox8c | -2.812956781 | 2.78E-30 |
| Cpn1 | -1.677713834 | 6.74E-06 |
| Cpt1b | -1.679750769 | 7.61E-17 |
| Crisp2 | -3.418886999 | 5.70E-29 |
| Csl | -2.565385025 | 2.63E-23 |
| Cyct | -1.48202456 | 1.68E-11 |
| D130017N08Rik | -1.763514478 | 6.92E-09 |
| D130043K22Rik | -1.809908849 | 8.37E-05 |
| Daw1 | -3.206699132 | 3.39E-12 |
| Dcdc2a | -2.370837444 | 4.22E-06 |
| Dis3l | -1.218220218 | 6.71E-23 |
| Dkkl1 | -3.180660423 | 1.99E-32 |
| Dlec1 | -1.364022662 | 2.14E-05 |
| Dmrtc2 | -1.116772683 | 5.16E-16 |
| Dnaaf1 | -1.643009743 | 1.36E-18 |
| Dnah1 | -1.956110182 | 8.57E-35 |
| Dnah17 | -2.456556771 | 8.35E-40 |
| Dnah2 | -1.021373611 | 4.57E-06 |
| Dnah6 | -3.24447138 | 9.12E-09 |
| Dnah7a | -2.21563557 | 9.75E-34 |
| Dnah7b | -1.346456337 | 4.34E-32 |
| Dnah8 | -2.440168735 | 1.79E-77 |
| Dnaic1 | -1.243993692 | 9.97E-11 |
| Dnajb13 | -2.988723346 | 2.47E-08 |
| Dnajb3 | -1.062564826 | 8.54E-05 |
| Dnajc5g | -1.222438712 | 0.000105683 |
| Dnali1 | -2.132657091 | 4.92E-09 |
| Dpy19l2 | -2.92212657 | 2.36E-18 |
| Drc1 | -2.860651399 | 3.23E-11 |
| Drc7 | -3.773937925 | 4.82E-13 |
| Dynlrb2 | -3.042311332 | 1.10E-09 |
| Dyx1c1 | -1.218740259 | 8.54E-05 |
| Efcab6 | -1.569048198 | 0.003401361 |
| Efhb | -1.462460235 | 0.042875263 |
| Efhc1 | -1.639776075 | 9.26E-13 |
| Eid3 | -3.163555421 | 1.52E-28 |
| Elfn2 | -2.656387022 | 9.11E-36 |
| Enkur | -1.061834991 | 0.001512331 |
| Epha10 | -2.063824288 | 0.001039911 |
| Eppk1 | -1.50605308 | 0.040702849 |
| Eps8l3 | -3.980139384 | 1.73E-10 |
| Erich2 | -2.784804366 | 3.25E-08 |
| Fabp9 | -2.618196429 | 2.37E-06 |
| Fam170a | -4.149970616 | 3.39E-11 |
| Fam181a | -4.256432753 | 2.17E-20 |
| Fam184a | -1.719278129 | 1.38E-09 |
| Fam186b | -2.8586862 | 2.51E-18 |
| Fam188b | -1.059225102 | 0.000163942 |
| Fam205a2 | -1.447290812 | 9.16E-11 |
| Fam227b | -1.446421698 | 0.01329615 |
| Fam228a | -1.627270233 | 4.50E-10 |
| Fam229b | -1.974673409 | 5.98E-11 |
| Fam50b | -2.943119493 | 2.55E-08 |
| Fank1 | -1.716805344 | 9.02E-12 |
| Fbp1 | -4.844897599 | 2.79E-51 |
| Fbxo43 | -1.225094634 | 3.48E-07 |
| Fbxw10 | -4.45588039 | 9.11E-09 |
| Fgb | -3.998084703 | 0.038502459 |
| Fhad1 | -1.720408454 | 6.13E-12 |
| Fhl4 | -1.588197857 | 4.32E-14 |
| Galnt3 | -1.401320822 | 0.000298892 |
| Gcnt3 | -2.826477478 | 0.006091307 |
| Ggn | -1.631706874 | 0.002783442 |
| Ggnbp1 | -1.772323468 | 2.99E-08 |
| Gk2 | -3.536405968 | 5.09E-23 |
| Gldnos | -5.77043255 | 0.002949784 |
| Glipr1l2 | -1.246206921 | 2.96E-17 |
| Gm10619 | -4.16799731 | 0.031340315 |
| Gm11837 | -1.150667162 | 8.28E-12 |
| Gm11992 | -1.229514228 | 0.000226946 |
| Gm128 | -1.778525823 | 1.25E-05 |
| Gm1527 | -5.102535811 | 0.030746694 |
| Gm15881 | -2.644122114 | 1.93E-09 |
| Gm16432 | -1.738605173 | 0.026687344 |
| Gm21269 | -3.501965706 | 7.63E-11 |
| Gm21284 | -2.275454166 | 1.54E-29 |
| Gm2762 | -2.217463265 | 1.57E-18 |
| Gm3417 | -2.490501793 | 6.51E-15 |
| Gm3448 | -2.490501793 | 6.51E-15 |
| Gm4763 | -2.397359541 | 1.71E-08 |
| Gm4871 | -6.278997329 | 0.000240917 |
| Gm4922 | -3.21306271 | 0.008036325 |
| Gm5134 | -1.611767017 | 4.38E-06 |
| Gm5136 | -1.476774825 | 0.000869573 |
| Gm5622 | -2.310488681 | 0.000751251 |
| Gm5878 | -5.330372987 | 3.85E-37 |
| Gm6455 | -5.598472195 | 0.001690553 |
| Gm6567 | -4.479924572 | 7.18E-33 |
| Gm6639 | -5.254132691 | 0.00042764 |
| Gm8267 | -1.685400319 | 0.000286857 |
| Gm8765 | -2.797004108 | 8.43E-06 |
| Gm906 | -3.308887435 | 0.014661729 |
| Gm9731 | -3.351733228 | 0.012011887 |
| Gnao1 | -1.107333554 | 2.77E-19 |
| Gramd1c | -1.276428567 | 2.45E-08 |
| Gsdmcl-ps | -5.684992201 | 0.001061483 |
| Gstt2 | -2.183367354 | 3.00E-23 |
| Gtf2a1l | -2.622172812 | 1.11E-11 |
| Gykl1 | -3.185990304 | 5.90E-11 |
| H2afy3 | -3.497835822 | 0.0413788 |
| Hdgfl1 | -2.092339862 | 5.78E-22 |
| Hdhd1a | -2.637637757 | 0.00726239 |
| Hrasls | -1.221623289 | 0.046807086 |
| Hrasls5 | -5.925389038 | 3.40E-13 |
| Hsf5 | -1.346552664 | 2.07E-19 |
| Hspa2 | -1.135271766 | 2.24E-26 |
| Hspb9 | -2.574905262 | 0.003394244 |
| Hydin | -2.46842656 | 2.05E-16 |
| Id4 | -1.05633006 | 1.11E-14 |
| Iglon5 | -1.073207984 | 0.000787018 |
| Insl6 | -2.699081062 | 1.55E-05 |
| Iqcd | -1.900538924 | 1.62E-13 |
| Iqch | -1.301506249 | 1.87E-09 |
| Iqub | -2.065446922 | 1.68E-06 |
| Irak3 | -1.269798085 | 2.52E-06 |
| Kbtbd6 | -1.062353062 | 4.88E-16 |
| Kcnj9 | -3.26392369 | 2.92E-05 |
| Kctd7 | -1.279174425 | 2.08E-22 |
| Khdrbs3 | -1.019374867 | 2.45E-09 |
| Kif4-ps | -2.641636 | 1.62E-07 |
| Kif9 | -1.091052424 | 8.79E-07 |
| Kng1 | -3.655533373 | 0.035827477 |
| LOC100125594 | -1.976428573 | 0.00369726 |
| Lca5l | -1.368222884 | 5.25E-17 |
| Ldhal6b | -3.132865305 | 2.42E-29 |
| Ldhc | -3.678301257 | 4.87E-20 |
| Lin7a | -2.521466302 | 9.82E-10 |
| Lrat | -1.831364479 | 1.44E-07 |
| Lrguk | -2.037089867 | 9.27E-15 |
| Lrrc23 | -3.596541752 | 1.24E-17 |
| Lrrc27 | -1.036838391 | 0.00030071 |
| Lrrc34 | -2.168949045 | 1.55E-23 |
| Lrrc36 | -1.355233361 | 1.98E-05 |
| Lrrc46 | -1.738186227 | 1.98E-08 |
| Lrrc6 | -1.760013129 | 3.42E-11 |
| Lrrc63 | -4.27245554 | 0.000199091 |
| Lrrc71 | -3.566464146 | 6.07E-48 |
| Lrrc73 | -1.090181899 | 0.000299815 |
| Lrriq1 | -1.601829443 | 0.003892945 |
| Lrriq3 | -1.368718407 | 9.93E-10 |
| Lypd4 | -2.348008772 | 2.30E-12 |
| Maats1 | -1.707271842 | 0.001427811 |
| Mag | -1.815965657 | 1.43E-06 |
| Mak | -1.553747619 | 8.24E-15 |
| Mapk15 | -2.997365086 | 1.94E-25 |
| Mapk8ip2 | -1.710526183 | 4.57E-12 |
| 10-Mar | -3.032175989 | 1.56E-25 |
| 11-Mar | -3.916555708 | 2.14E-13 |
| Mdh1b | -2.872571145 | 4.45E-20 |
| Med9os | -1.612667088 | 0.001006824 |
| Meig1 | -1.950955511 | 9.20E-15 |
| Meikin | -1.415923969 | 5.28E-05 |
| Mfsd6l | -2.280771672 | 0.002051068 |
| Mgat4d | -3.841519041 | 3.27E-08 |
| Mlc1 | -1.159842291 | 2.16E-09 |
| Morc2b | -2.247490889 | 1.19E-26 |
| Morn5 | -1.691833424 | 1.36E-06 |
| Mospd4 | -2.884079001 | 0.006698131 |
| Mroh2a | -4.388292505 | 8.30E-22 |
| Mroh2b | -4.158768574 | 5.09E-29 |
| Mroh4 | -2.920108951 | 5.70E-47 |
| Mroh8 | -1.960696838 | 8.77E-14 |
| Ms4a13 | -3.346911661 | 1.53E-07 |
| Mtl5 | -1.148966353 | 3.80E-11 |
| Myl10 | -2.154491834 | 0.013059228 |
| Ndufaf3 | -1.372893428 | 5.47E-09 |
| Nek11 | -1.358795037 | 0.001558085 |
| Nkapl | -1.202659126 | 0.000665811 |
| Nlrp9b | -5.743005482 | 0.004374595 |
| Nme5 | -1.217417078 | 0.007912252 |
| Nps | -2.296108937 | 0.023192328 |
| Nsun7 | -1.519959676 | 4.24E-13 |
| Nup210l | -1.166767943 | 4.90E-09 |
| Nup62-il4i1 | -2.052054511 | 1.67E-06 |
| Odf4 | -2.947957638 | 1.73E-17 |
| Oit1 | -4.795038883 | 4.51E-05 |
| Oscp1 | -1.248865085 | 1.43E-06 |
| Pabpc2 | -4.321486697 | 6.95E-19 |
| Pabpc6 | -1.280500809 | 4.74E-08 |
| Pacrg | -1.02136298 | 0.000428012 |
| Papolb | -1.244879689 | 2.25E-06 |
| Pbp2 | -3.525819302 | 1.70E-09 |
| Pbx4 | -1.648290865 | 1.46E-13 |
| Pcsk4 | -1.154008474 | 1.12E-08 |
| Pdcl2 | -2.252686126 | 9.69E-22 |
| Pde1c | -2.152121112 | 1.10E-27 |
| Pdha2 | -1.376367036 | 3.31E-23 |
| Pebp4 | -4.257995149 | 1.22E-08 |
| Perm1 | -1.080458491 | 0.00200357 |
| Pex11g | -1.195705062 | 3.00E-06 |
| Pfn4 | -1.453646396 | 2.96E-07 |
| Pgam2 | -3.598975348 | 8.29E-18 |
| Pgk2 | -3.84073739 | 3.23E-05 |
| Phf7 | -1.346797003 | 3.11E-11 |
| Pifo | -3.663840395 | 5.74E-13 |
| Pih1d3 | -1.638545513 | 0.018750198 |
| Piwil1 | -3.235174766 | 1.98E-148 |
| Pkmyt1 | -1.412241981 | 5.11E-23 |
| Pla2g2c | -1.081623275 | 0.000449088 |
| Plcd4 | -1.966944327 | 0.012336567 |
| Pmp2 | -2.183434911 | 1.22E-06 |
| Pnma1 | -1.719084615 | 5.61E-16 |
| Pnmal1 | -2.237462792 | 2.21E-15 |
| Pom121l12 | -4.528015754 | 9.93E-06 |
| Pom121l2 | -4.217277536 | 2.20E-15 |
| Poteg | -4.682080224 | 1.98E-06 |
| Pou2f2 | -1.822672619 | 2.97E-07 |
| Ppp1r36 | -2.24333205 | 2.27E-12 |
| Ppp1r42 | -2.137038027 | 0.018179267 |
| Ppp3r2 | -2.944782276 | 2.28E-08 |
| Prdx6b | -6.496839949 | 2.86E-06 |
| Prok2 | -4.26617137 | 4.01E-33 |
| Prps1l1 | -3.557390679 | 1.61E-14 |
| Prss43 | -2.271681625 | 0.000564953 |
| Prss44 | -1.914371844 | 7.66E-12 |
| Prss45 | -1.840156945 | 0.000238114 |
| Psma8 | -1.749874525 | 4.27E-22 |
| Ptchd3 | -3.051672061 | 1.19E-47 |
| Ptpn20 | -1.555756642 | 1.92E-17 |
| Ptprq | -1.878343097 | 0.004705765 |
| Pxt1 | -1.267961368 | 0.005832379 |
| Qrich2 | -5.063501829 | 8.98E-16 |
| Rbakdn | -5.97103467 | 2.42E-34 |
| Rfx4 | -3.440201478 | 1.55E-18 |
| Rfx8 | -1.739980972 | 2.22E-06 |
| Riiad1 | -1.485984101 | 0.00075118 |
| Rimbp3 | -1.435402416 | 1.09E-06 |
| Rnf32 | -1.617057629 | 2.97E-17 |
| Rnls | -1.273130921 | 2.47E-07 |
| Ropn1l | -2.188246475 | 1.58E-18 |
| Rpgrip1 | -1.531227244 | 5.28E-05 |
| Rsph1 | -1.57401416 | 4.32E-14 |
| Rsph6a | -3.501533005 | 3.40E-06 |
| Rsph9 | -1.530690985 | 1.01E-05 |
| Samd7 | -2.063151364 | 0.000150404 |
| Scgb2b23-ps | -2.88936385 | 6.70E-05 |
| Sel1l2 | -5.850538991 | 0.001717936 |
| 12-Sep | -2.336024429 | 2.07E-11 |
| Serpina1c | -2.557188073 | 0.017863506 |
| Sgca | -2.936983835 | 0.000100793 |
| Shcbp1l | -1.475156619 | 4.49E-18 |
| Slc13a5 | -3.305586835 | 0.00011103 |
| Slc22a16 | -4.118899053 | 2.49E-11 |
| Slc2a3 | -2.28291305 | 2.14E-51 |
| Slc2a5 | -2.508061781 | 5.36E-24 |
| Slc30a3 | -1.1771947 | 0.002910235 |
| Slc8a2 | -1.018950812 | 2.01E-14 |
| Slc9b1 | -1.30134833 | 2.17E-08 |
| Slc9c1 | -2.682734879 | 0.002730034 |
| Slco6c1 | -7.679129217 | 6.01E-08 |
| Slco6d1 | -3.893494471 | 0.015856609 |
| Smco2 | -1.733831569 | 0.014069955 |
| Smim24 | -2.456677934 | 7.92E-12 |
| Smpd5 | -2.887340011 | 1.13E-56 |
| Sord | -1.438609244 | 2.85E-22 |
| Spag16 | -2.109428554 | 9.56E-07 |
| Spag17 | -3.715987823 | 5.14E-44 |
| Spag6 | -3.656280365 | 1.80E-32 |
| Spag8 | -2.312850425 | 2.18E-08 |
| Spata16 | -3.535128503 | 5.10E-13 |
| Spata17 | -1.011294309 | 0.047639705 |
| Spata18 | -2.540946359 | 0.004546551 |
| Spata24 | -1.087649957 | 0.00014477 |
| Spata31d1a | -2.23761115 | 1.61E-05 |
| Spata33 | -1.141247636 | 0.000969616 |
| Spata4 | -2.793475339 | 1.20E-34 |
| Spats1 | -2.730328807 | 3.36E-09 |
| Spdye4a | -3.04113089 | 0.001896695 |
| Spdye4b | -2.121811892 | 4.97E-17 |
| Speer1 | -8.135268118 | 8.75E-09 |
| Speer2 | -4.821726024 | 0.003077926 |
| Spef2 | -1.358248952 | 0.005748265 |
| Spesp1 | -1.490419466 | 2.39E-12 |
| Sphkap | -3.273841647 | 1.45E-13 |
| Spink2 | -4.983994441 | 5.67E-18 |
| Ssmem1 | -1.362004786 | 0.002153946 |
| Stambp | -1.148556569 | 1.62E-11 |
| Stk33 | -1.287650577 | 4.04E-10 |
| Stkld1 | -1.922373647 | 0.000152364 |
| Sun3 | -3.390905466 | 0.000395053 |
| Susd5 | -1.447858067 | 8.60E-05 |
| Svop | -3.581553819 | 6.11E-07 |
| Svopl | -5.029814844 | 0.046143993 |
| Syce1l | -1.662124486 | 0.013114706 |
| Syngr4 | -1.265312604 | 2.66E-10 |
| Tcam1 | -1.670328054 | 8.69E-25 |
| Tcea2 | -1.160273173 | 1.04E-06 |
| Tcp10a | -3.277124584 | 3.85E-12 |
| Tcp10b | -4.082156106 | 1.51E-06 |
| Tcp10c | -7.606316151 | 3.30E-07 |
| Tcp11 | -3.716550231 | 5.46E-43 |
| Tcte1 | -2.78642945 | 0.000414128 |
| Tcte2 | -1.364750707 | 0.010741023 |
| Tcte3 | -1.795831463 | 6.44E-07 |
| Tctex1d2 | -1.008299648 | 4.51E-07 |
| Tdrd1 | -1.017734297 | 4.32E-23 |
| Tdrd12 | -1.019728915 | 3.23E-12 |
| Tdrd5 | -1.365196014 | 7.66E-21 |
| Tdrd6 | -3.830931387 | 1.70E-55 |
| Tekt1 | -2.762822327 | 3.32E-19 |
| Tekt4 | -2.872059896 | 0.00268197 |
| Tex22 | -3.103650735 | 0.001330554 |
| Tex40 | -3.587274432 | 6.92E-11 |
| Thegl | -3.545044012 | 0.01651578 |
| Tkfc | -1.091816863 | 1.73E-10 |
| Tktl2 | -1.053028681 | 4.17E-15 |
| Tmbim7 | -5.617474611 | 9.89E-15 |
| Tmem232 | -1.388425953 | 0.000711356 |
| Tmem30c | -3.963880989 | 7.00E-16 |
| Tmem89 | -5.127045684 | 0.00616694 |
| Tmprss12 | -1.917424071 | 4.02E-08 |
| Trim69 | -3.748820006 | 0.000700282 |
| Trpd52l3 | -1.284365372 | 1.42E-06 |
| Tsacc | -1.171086618 | 0.000543542 |
| Tsnaxip1 | -2.722259277 | 5.20E-20 |
| Ttbk1 | -1.131880988 | 3.81E-11 |
| Ttc16 | -1.027221233 | 2.59E-05 |
| Ttc21a | -1.196535315 | 0.000381836 |
| Ttc25 | -1.483135641 | 7.07E-07 |
| Ttc29 | -2.798846989 | 0.000137867 |
| Ttc30a2 | -2.543667782 | 9.82E-15 |
| Ttc39d | -1.183709039 | 0.016857603 |
| Ttll13 | -1.387994196 | 1.39E-11 |
| Ttll6 | -1.960882633 | 1.17E-10 |
| Ttll9 | -1.089406838 | 0.002161698 |
| Tuba3b | -1.0088693 | 3.16E-07 |
| Ube2d2b | -1.95072879 | 3.01E-09 |
| Ube2u | -1.619191876 | 1.83E-08 |
| Usp2 | -1.306915246 | 2.73E-15 |
| Usp44 | -1.398860743 | 0.010762604 |
| Wdr20rt | -1.488598838 | 2.24E-05 |
| Wdr63 | -3.185246134 | 1.03E-19 |
| Wdr93 | -1.48386364 | 3.12E-11 |
| Wfdc15a | -1.068451379 | 3.59E-05 |
| Xrra1 | -1.347767001 | 2.84E-07 |
| Ybx2 | -1.501523972 | 5.57E-28 |
| Ypel1 | -1.071561137 | 4.57E-08 |
| Zbtb3 | -1.030935311 | 6.56E-05 |
| Zfa-ps | -1.786435335 | 1.85E-06 |
| Zfand4 | -1.008020505 | 6.51E-15 |
| Zfp389 | -1.931799494 | 2.72E-05 |
| Zfp572 | -2.699708416 | 0.049746758 |
| Zfp820 | -2.257654649 | 7.09E-27 |
| Zmynd10 | -3.133826564 | 2.14E-43 |
| Zmynd12 | -1.689230488 | 0.011904573 |
| Zpbp2 | -1.617252788 | 4.21E-05 |
| Zswim2 | -3.208424077 | 0.024945098 |
