# Supplementary material for: Human-specific gene CT47 blocks PRMT5 degradation to lead to meiosis arrest
Source: Cell Death Discov. 2022 Aug 2;8:345. doi: 10.1038/s41420-022-01139-6 (PMC9345867; doi:10.1038/s41420-022-01139-6)
Supplement: Supplementary file 14 — Table S3 [file 41420_2022_1139_MOESM14_ESM.docx]

**Table S3. Resources table of experimental material.**

| **Experimental Models：Organisms/Strains** | | | | |
| --- | --- | --- | --- | --- |
| Mouse: C57BL/6J | | | | |
| Mouse: *CT47*-BAC | | | | |
| **Biological Sample** | | | | |
| Testis samples of obstructive and nonobstructive azoospermia patients | | | | |
| **Antibodies** | | | | |
| CT47 | Homemade |  | 1:1000(WB); 1:200(IF) | |
| PRMT5 | Millipore | 07-405 | 1:1000(WB); 1:200(IF) | |
| SYCP3 | Invitrogen | ab97672 | 1:200(IF) | |
| γH2AX | Abcam | ab11174 | 1:1000(IF) | |
| ZBTB16 | R&D Systems | AF2944 | 1:400(IF) | |
| FLAG | Sigma–Aldrich | F1804 | 1:1000(WB) | |
| HA | Sigma–Aldrich | A2095 | 1:1000(WB) | |
| H4R3me2s | Abcam | ab5823 | 1:100(IF); 1:500(WB) | |
| **Reagents** | | | | |
| mTeSR™1 Complete Kit | | STEMCELL | | #85850 |
| Accutase | | Millipore | | #07923 |
| DMEM/F12, Glutamax^TM^ | | ThermoFisher | | 10565018 |
| DMEM/HIGH GLUCOSE | | HyClone | | AF29483100 |
| Fetal bovine serum | | Gibco | | 10270 |
| Corn oil | | Absin | | abs42015838 |
| Matrixgel® Matrix | | Corning | | 354227 |
| KnockOut™ Serum Replacement | | ThermoFisher | | 10828010 |
| B-27™ Supplement | | Gibco | | 17504044 |
| MEM nonessential amino acids | | Gibco | | 11140-050 |
| Pen/strep | | Gibco | | 15070-063 |
| **Chemicals** | | | | |
| ROCK inhibitor | | Selleckchem | | Y-27632 |
| Cycloheximide | | Sigma–Aldrich | | C1988 |
| MG132 | | Sigma | | M8699 |
| Doxycycline hyclate | | Sigma | | D9891 |
| β-mercaptoethanol | | Gibco | | 21985023 |
| Testosterone proplonate | | 3A Chemical | | A42318 |
| Puromycin | | Sangon Biotech | | A610593 |
| Blasticidin | | Sigma | | 15205 |
| Testosterone | | Yeasen | | 60803ES08 |
| L-glutamine | | Gibco | | 25030081 |
| Sodium pyruvate | | Gibco | | 11360070 |
| **Recombinant Proteins** | | | | |
| bFGF | | BD Biosciences | | 356061 |
| BMP4 | | R&D Systems | | 314-BP |
| LIF | | STEM CELL | | 78055 |
| SCF | | R&D Systems | | 7466-SC |
| EGF | | R&D Systems | | 236-EG |
| Insulin | | ThermoFisher | | RP-10908 |
| GDNF | | R&D Systems | | 212-GD |
| **Critical Commercial Assays** | | | | |
| FLAG® HA Tandem Affinity Purification Kit | | Sigma–Aldrich | | TP0010-5RXN |
| ProteoSilver™ Plus Silver Stain Kit | | Sigma–Aldrich | | PROT-SIL2 |
| ApopTag® Fluorescein Situ Apotosis Detection Kit | | Millipore | | S7110 |
| P3 Primary Cell 4D-Nucleofector™ X Kit L | | Lonza | | V4XP-3024 |
| Testosterone ELISA Kit | | Abcam | | ab108666 |
| **Oligonucleotides** | | | | |
| **Primers for *CT47*-BAC mice genotyping** | | | | |
| Primer for *CT47*-BAC mice Forward | | CTGGGATTGGACCTGAG | | |
| Primer for *CT47*-BAC mice Reverse | | GGGTTGTGCCGCCTTT | | |
| **Primers for KO cell line identification** | | | | |
| CT47-F | | GTCTAAGTCCTCTCAGGCCAG | | |
| CT47-R | | ACCACCGCCAAGTCGAAGTTG | | |
| **Primer for CT47 expression** | | | | |
| CT47-F | | TGTTACAATTCCCACAGGCA | | |
| CT47-R | | AGAGAAGGATGCGGAAAACA | | |
| **gRNA used to generate CT47-KO hESC line** | | | | |
| CT47-1 | | GACCACCGCCAAGTCGAAGT TGG | | |
| CT47-2 | | GGTGGTCGCCCGTCGCTACC CGG | | |
| **Primers for copy number identification** | | | | |
| Primer for copy number Forward 1 | | TTTCCGTATCTACCATGTGGG | | |
| Primer for copy number Reverse 1 | | ATTGGTAATGAAGTCTTAAGG | | |
| Primer for copy number Forward 2 | | AGTGTCCCCATGAAGTGCTAG | | |
| Primer for copy number Reverse 2 | | TCACCAGGTTAAGCCATGCAG | | |
