# Supplementary material for: Human-specific gene CT47 blocks PRMT5 degradation to lead to meiosis arrest
Source: Cell Death Discov. 2022 Aug 2;8:345. doi: 10.1038/s41420-022-01139-6 (PMC9345867; doi:10.1038/s41420-022-01139-6)
Supplement: Supplementary file 15 — original western data [file 41420_2022_1139_MOESM15_ESM.pptx]

## Slide 1
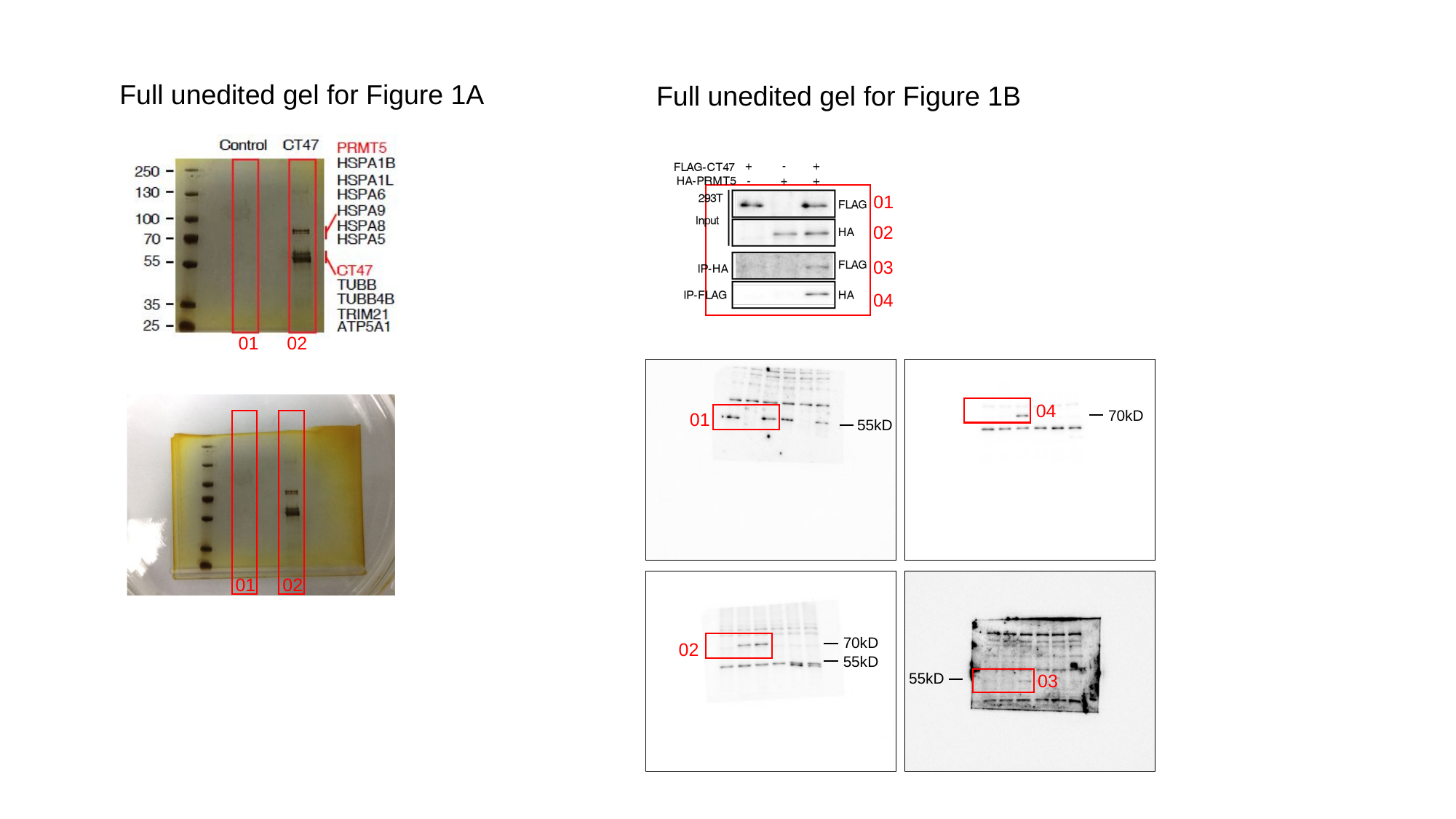

Full unedited gel for Figure 1A
Full unedited gel for Figure 1B
01
02
01
02
03
04
04
01
02
03
02
01
70kD
55kD
70kD
55kD
55kD

## Slide 2
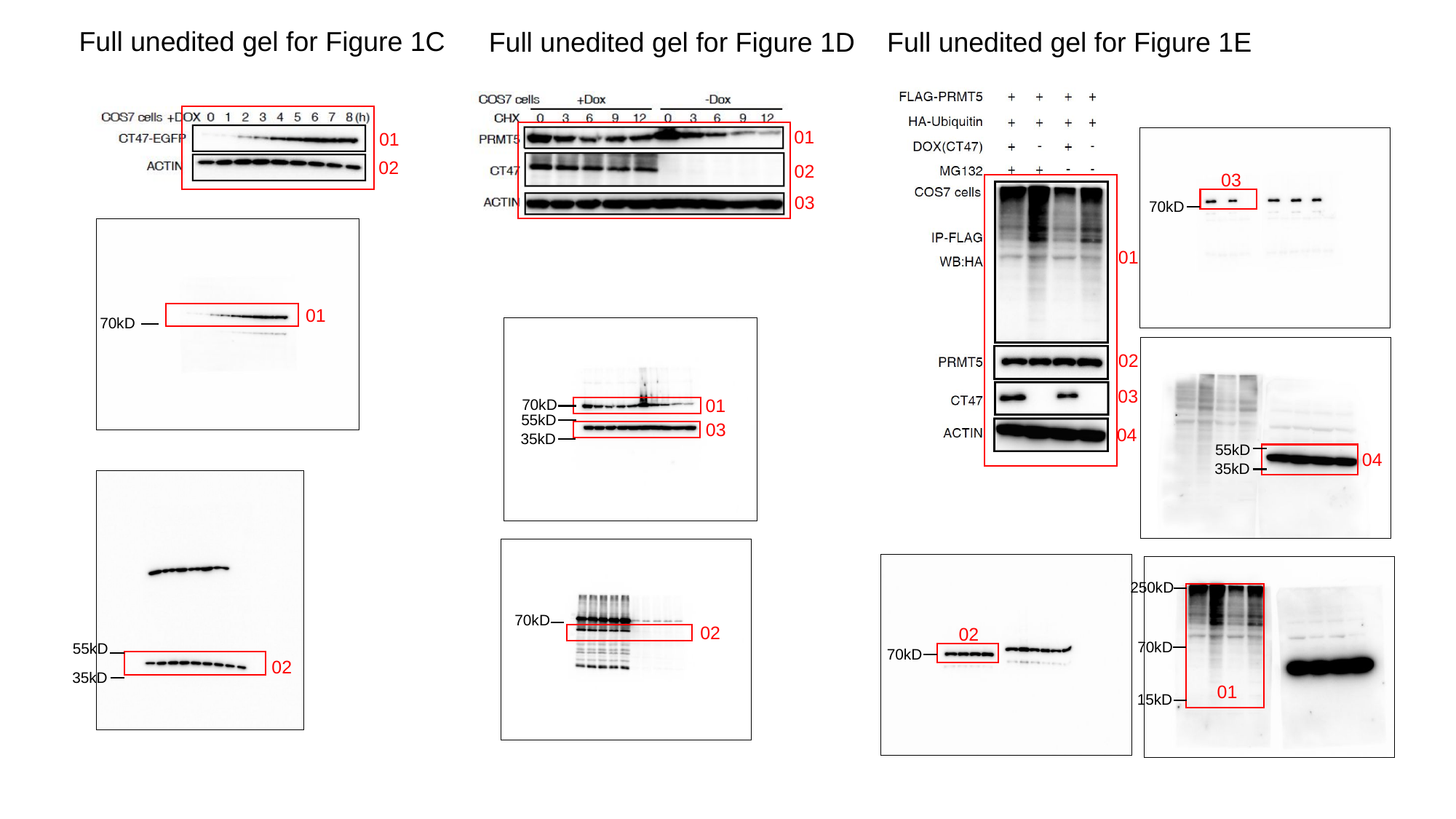

Full unedited gel for Figure 1C
Full unedited gel for Figure 1D
Full unedited gel for Figure 1E
01
01
02
02
03
03
70kD
01
01
70kD
02
03
01
70kD
55kD
03
04
35kD
55kD
04
35kD
250kD
70kD
02
02
70kD
55kD
70kD
02
35kD
01
15kD

## Slide 3
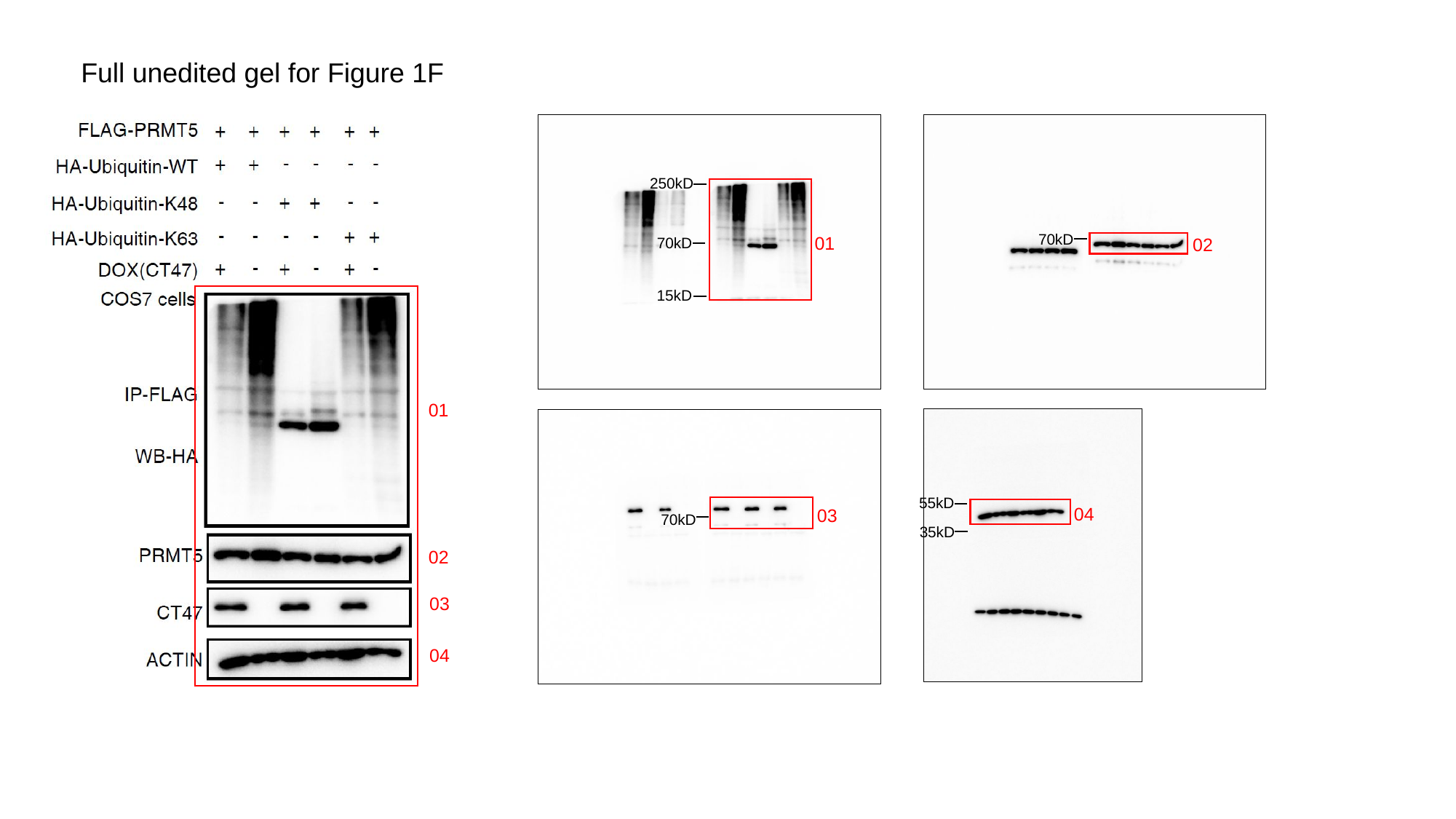

Full unedited gel for Figure 1F
250kD
70kD
01
70kD
02
15kD
01
55kD
04
03
70kD
35kD
02
03
04

## Slide 4
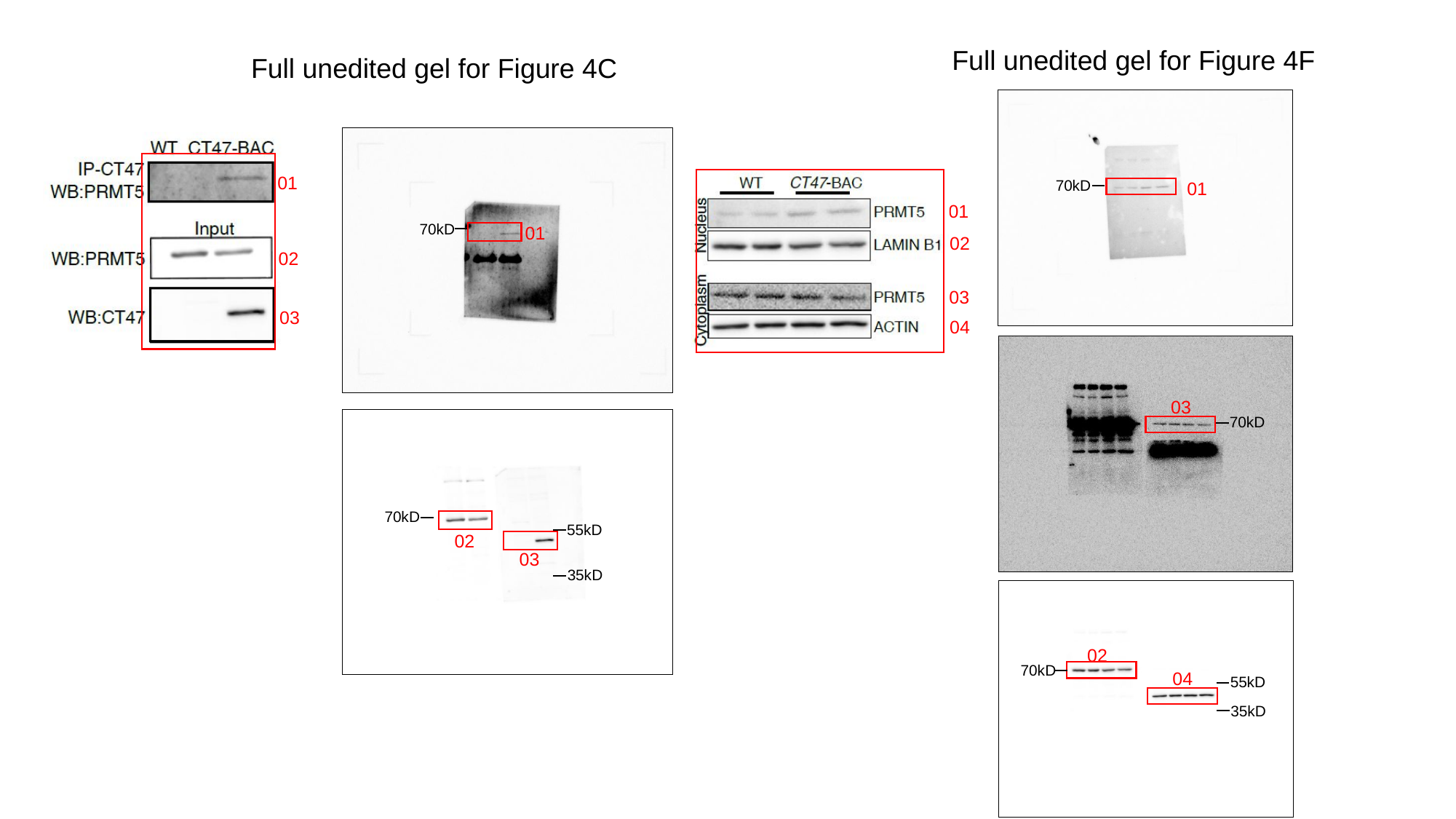

Full unedited gel for Figure 4F
Full unedited gel for Figure 4C
01
70kD
01
01
70kD
01
02
02
03
03
04
03
70kD
70kD
55kD
02
03
35kD
02
70kD
04
55kD
35kD

## Slide 5
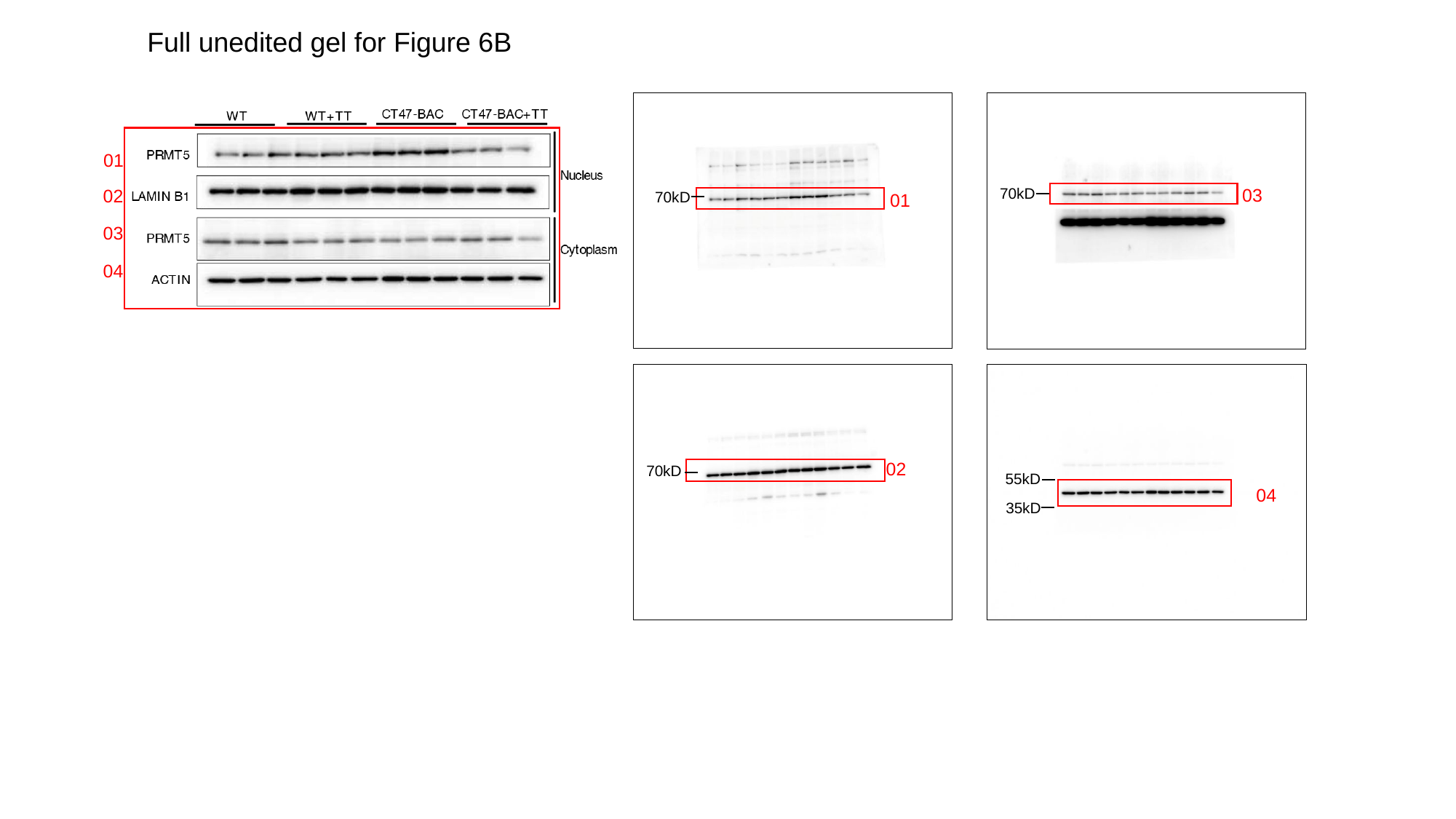

Full unedited gel for Figure 6B
03
01
70kD
02
70kD
01
03
04
02
70kD
55kD
04
35kD

## Slide 6
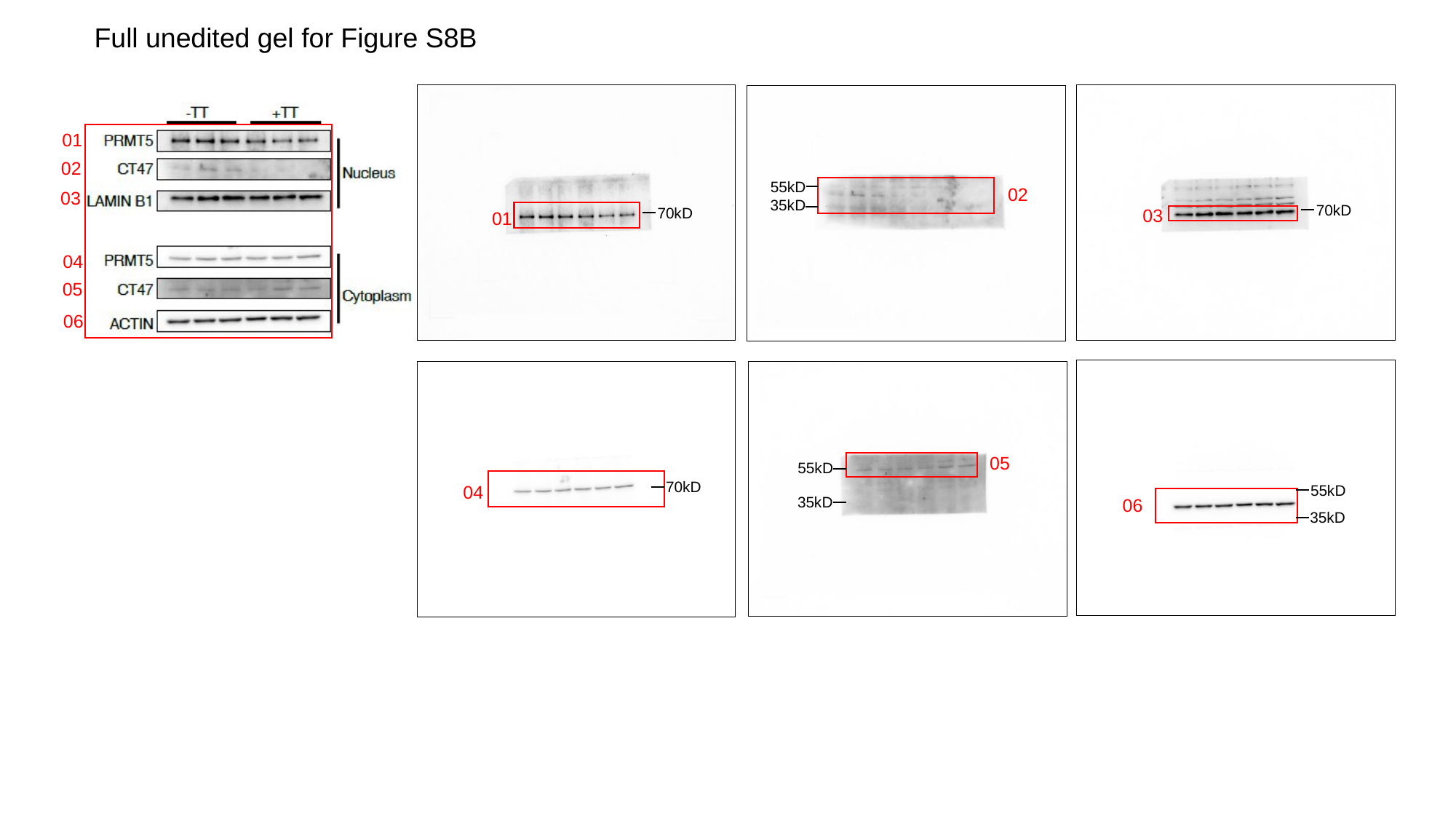

Full unedited gel for Figure S8B
02
01
02
55kD
03
35kD
70kD
70kD
03
01
04
05
06
05
04
55kD
70kD
55kD
35kD
06
35kD

## Slide 7
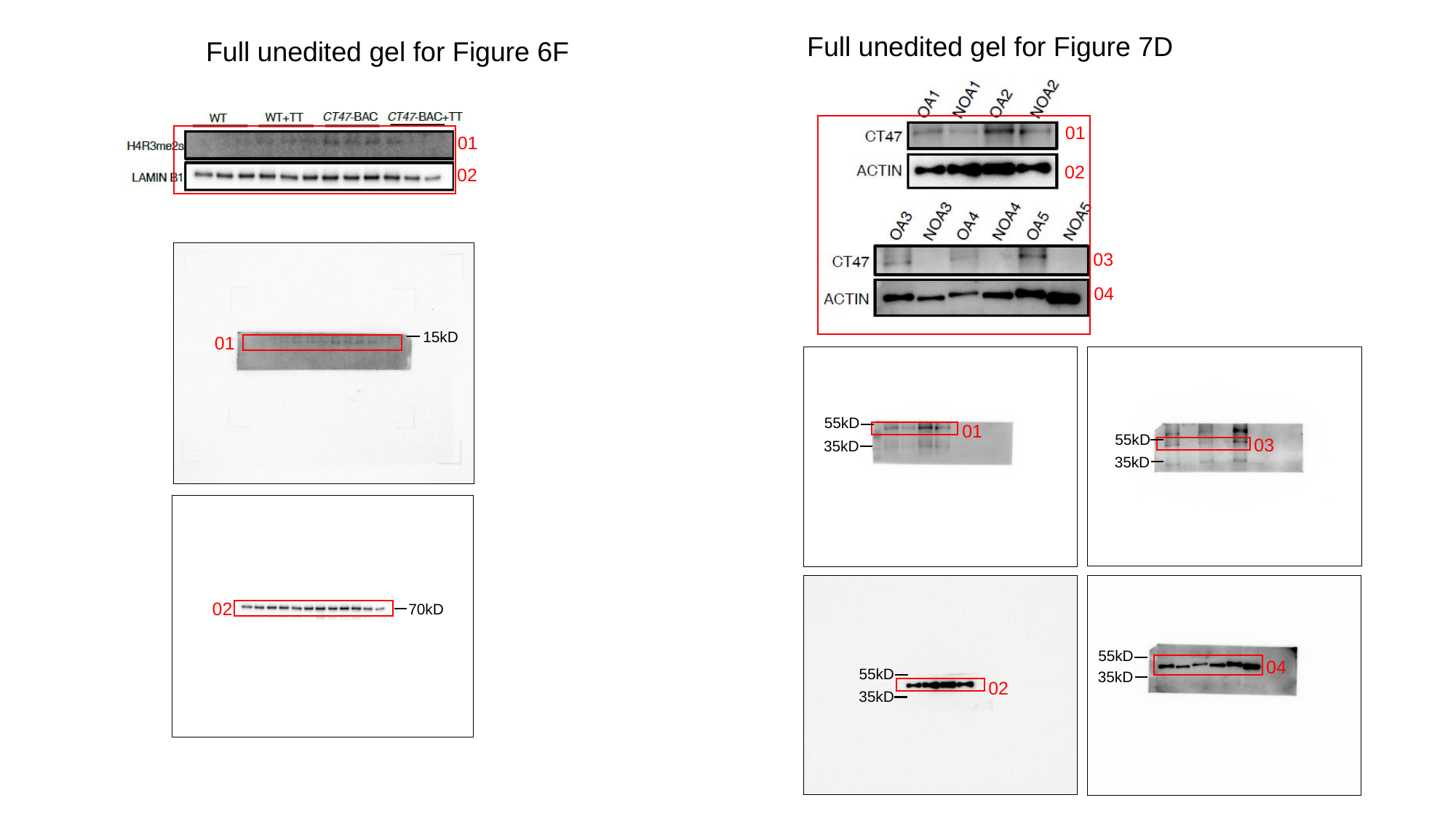

Full unedited gel for Figure 7D
Full unedited gel for Figure 6F
01
02
03
04
01
02
15kD
01
01
03
04
02
55kD
55kD
35kD
35kD
02
70kD
55kD
55kD
35kD
35kD
