# Supplementary material for: Human-specific gene CT47 blocks PRMT5 degradation to lead to meiosis arrest
Source: Cell Death Discov. 2022 Aug 2;8:345. doi: 10.1038/s41420-022-01139-6 (PMC9345867; doi:10.1038/s41420-022-01139-6)

| **FirstName** | **LastName** | **Email** |
| --- | --- | --- |
| Yingying | Dong | yydong@suda.edu.cn |
| Yi | Liu | yi.liu@utsouthwestern.edu |
| Steve D M | Brown | [s.brown@har.mrc.ac.uk](mailto:s.brown@har.mrc.ac.uk) |
| Chengji J | Zhou | [cjzhou@ucdavis.edu](mailto:cjzhou@ucdavis.edu) |
| Bing | Yao | [yaobing@nju.edu.cn](mailto:yaobing@nju.edu.cn) |
| Xin | Wu | [xinwu@njmu.edu.cn](mailto:xinwu@njmu.edu.cn) |
| Yuming | Feng | [fyming2013@126.com](mailto:fyming2013@126.com) |
| Zhengyun | Huang | [20184250016@stu.suda.edu.cn](mailto:20184250016@stu.suda.edu.cn) |
| Chao | Li | [chaoli1031@126.com](mailto:chaoli1031@126.com) |
| Yue | Gu | [20204050001@stu.suda.edu.cn](mailto:20204050001@stu.suda.edu.cn) |
| Yichen | Zhu | [zyc0728@suda.edu.cn](mailto:zyc0728@suda.edu.cn) |
| Junjie | Deng | [buzhimingtiankong@163.com](mailto:buzhimingtiankong@163.com) |
| Moli | Huang | [huangml@suda.edu.cn](mailto:huangml@suda.edu.cn) |
| Zhiwei | Liu | [zwliu@suda.edu.cn](mailto:zwliu@suda.edu.cn) |
| Shijun | Hu | [shijunhu@suda.edu.cn](mailto:shijunhu@suda.edu.cn) |
| Tao | Wang | [wangtao36@suda.edu.cn](mailto:wangtao36@suda.edu.cn) |
| Zhenxin | Fu | fzx17658133973@163.com |
| Hanben | Wang | ziderum@njmu.edu.cn |
| Yizhun | Zeng | 20194250014@stu.suda.edu.cn |
| Antonio | Vidal-Puig | ajv22@medschl.cam.ac.uk |


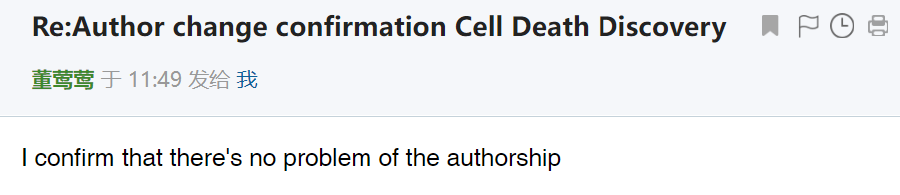


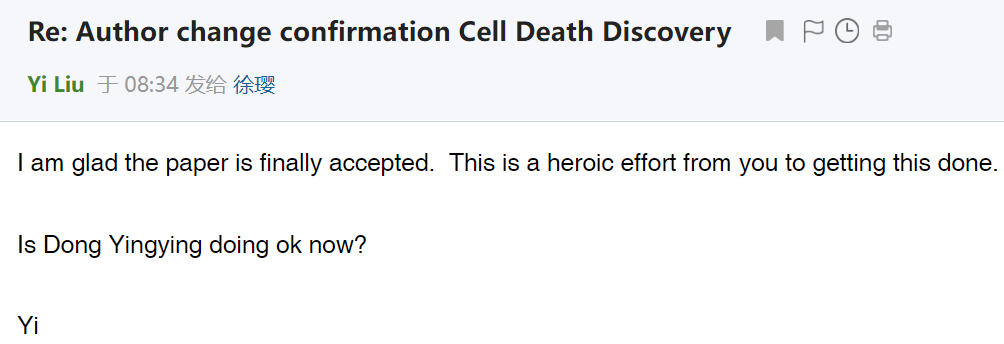


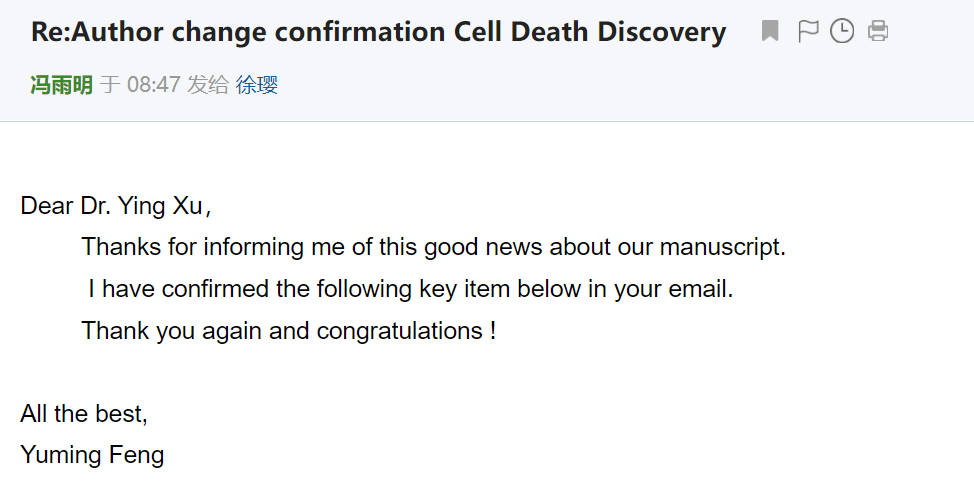


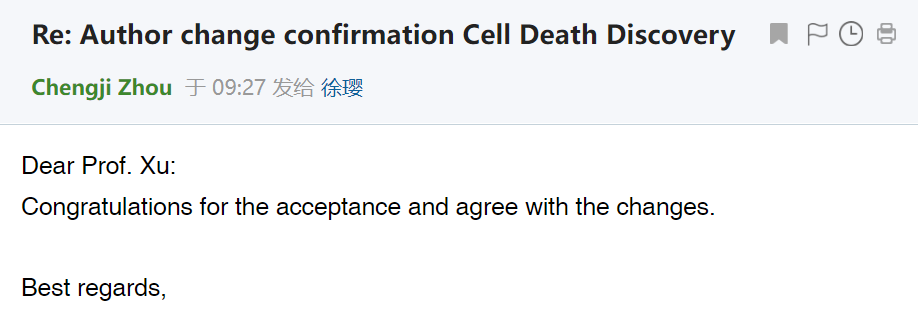


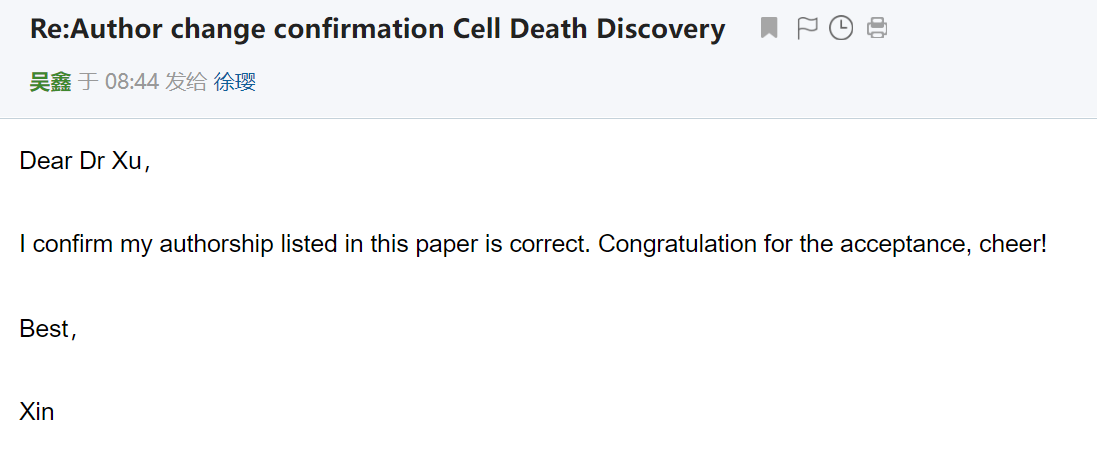


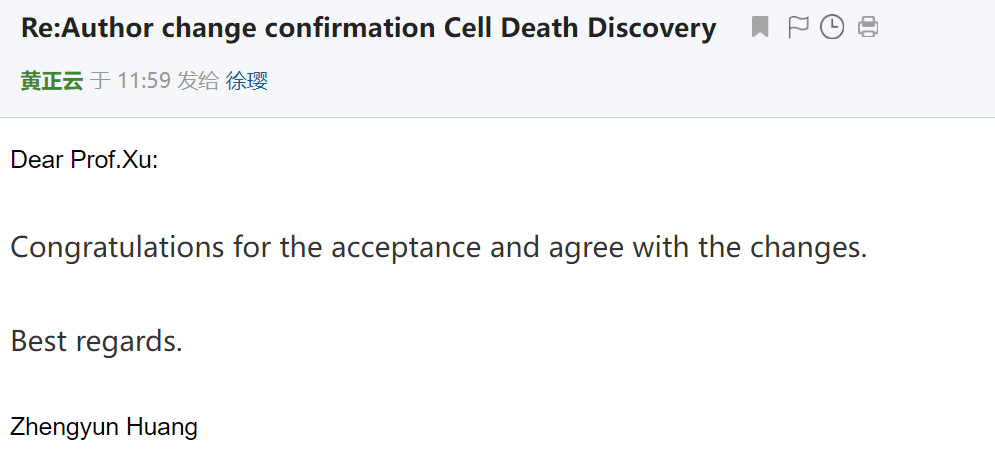


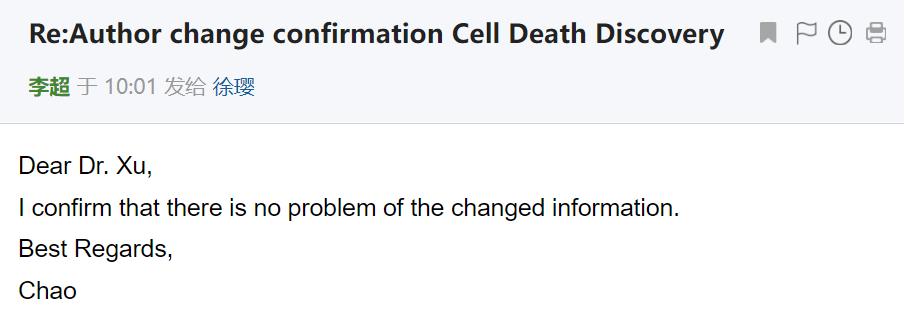


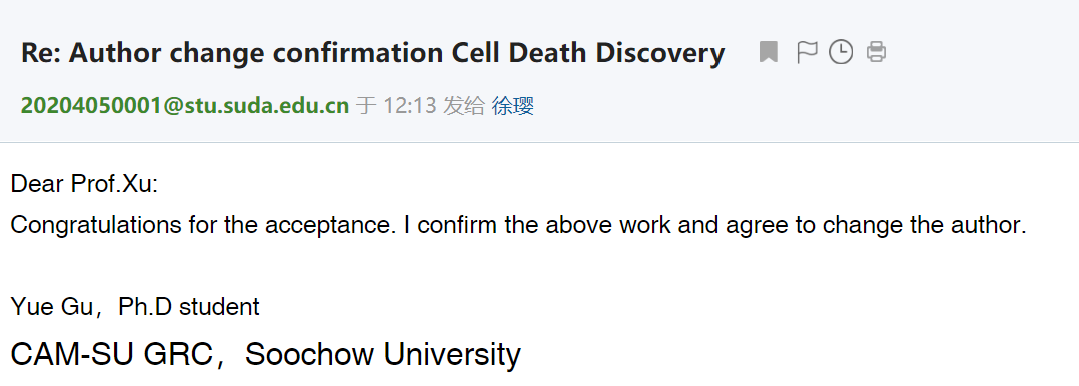


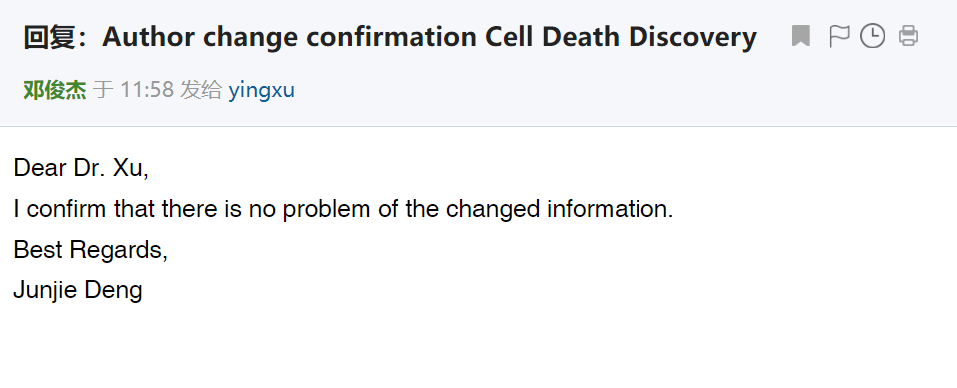


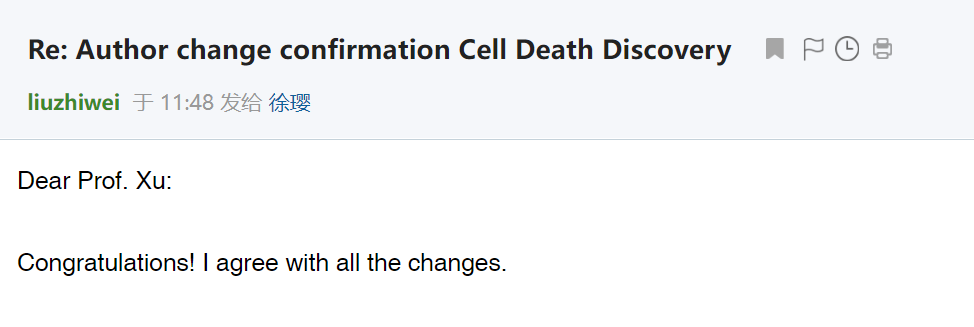


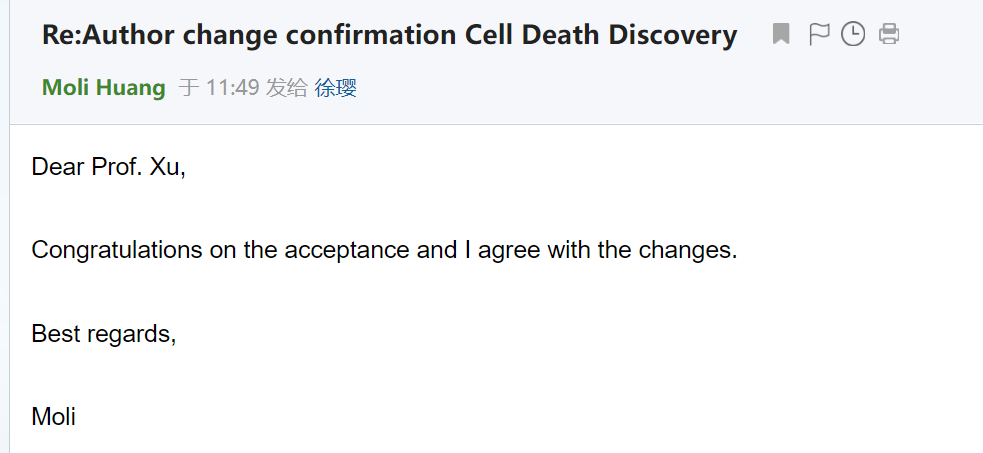


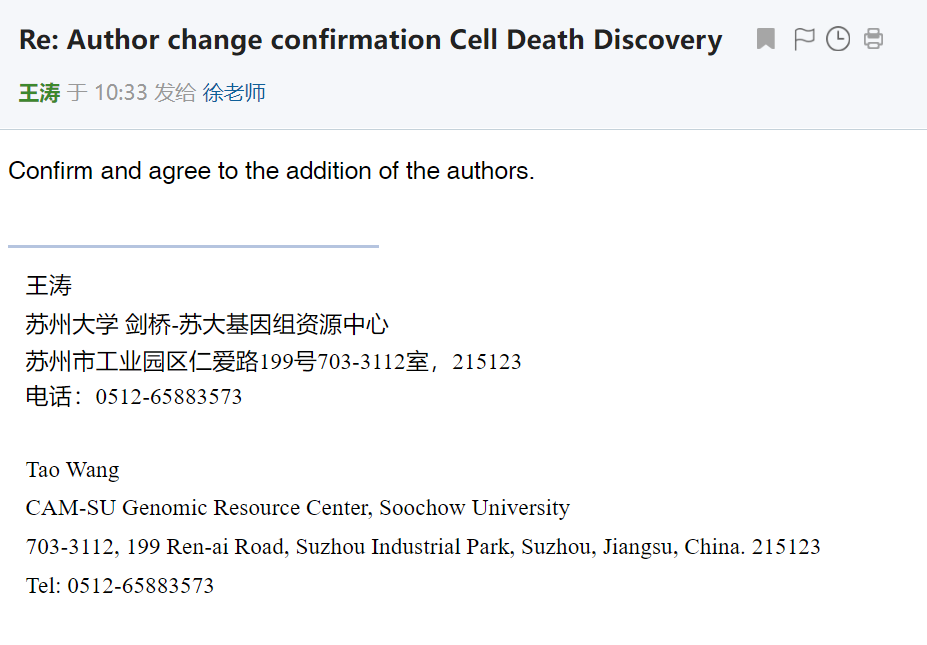


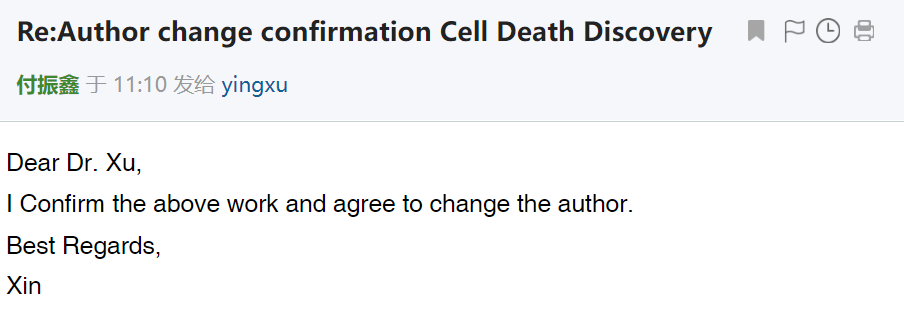


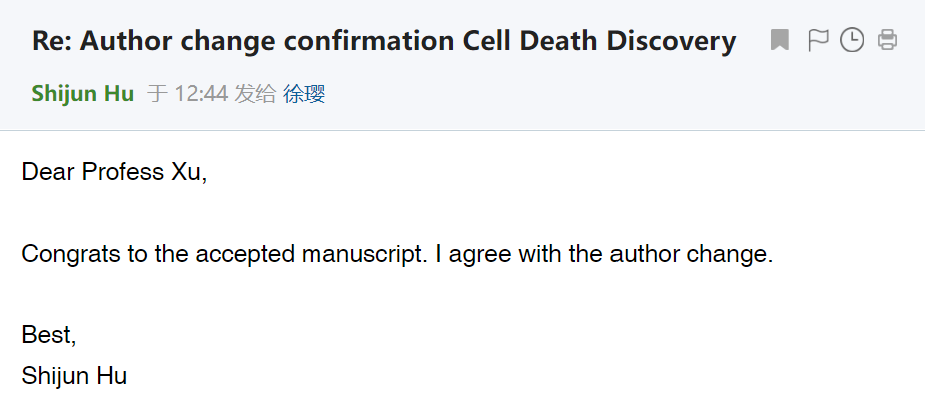


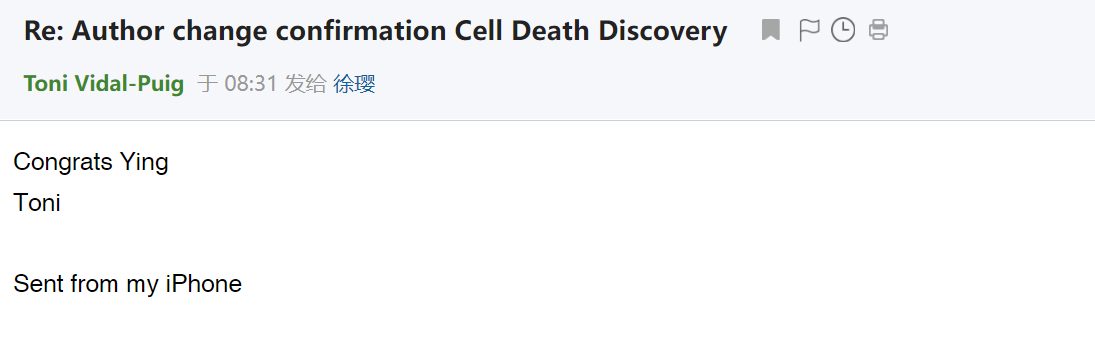


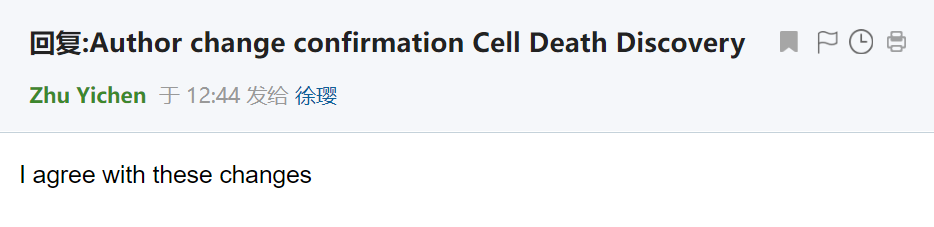


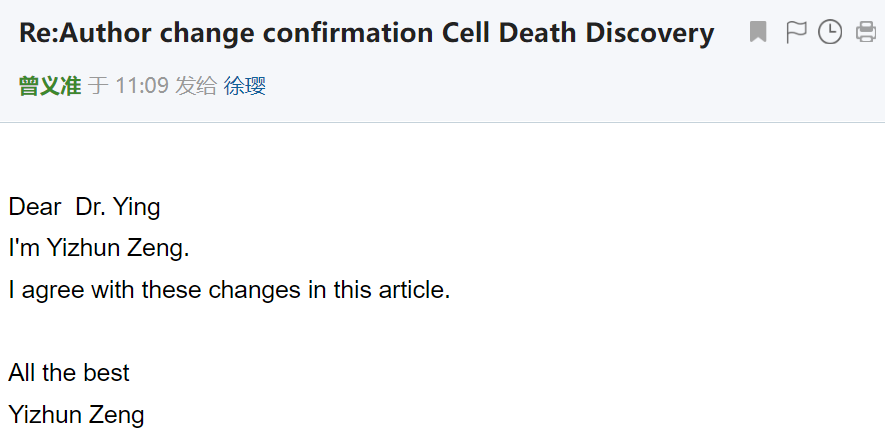


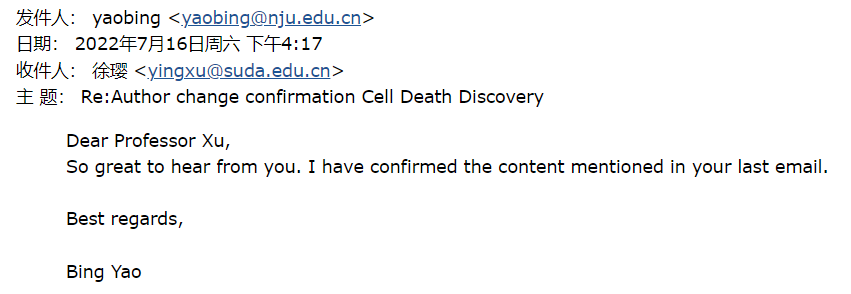


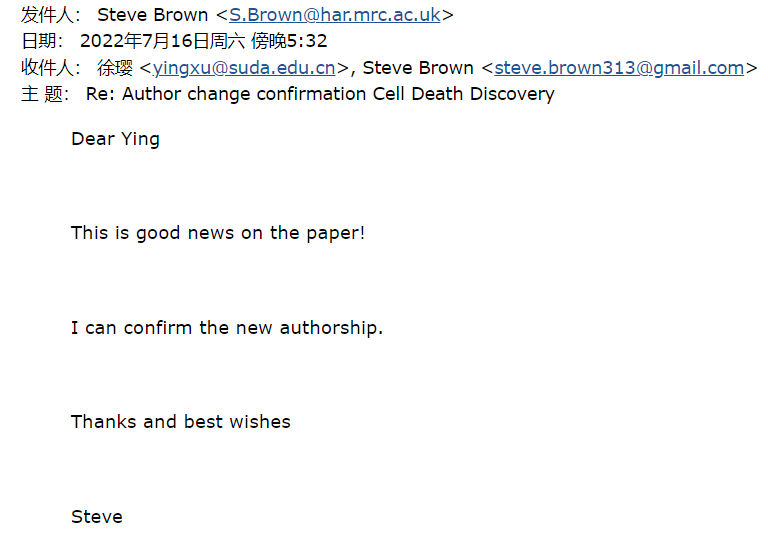


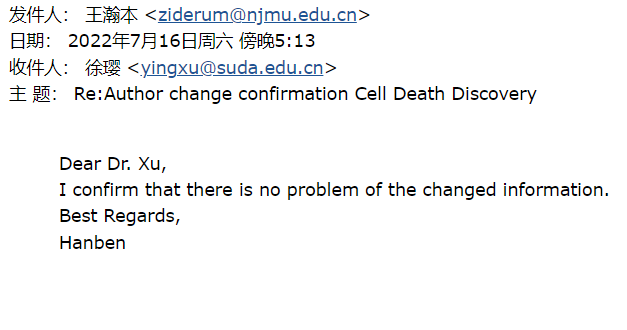

Supplement: Supplementary file 16 — Authorship confirmed [file 41420_2022_1139_MOESM16_ESM.docx]
