# Supplementary material for: Human-specific gene CT47 blocks PRMT5 degradation to lead to meiosis arrest
Source: Cell Death Discov. 2022 Aug 2;8:345. doi: 10.1038/s41420-022-01139-6 (PMC9345867; doi:10.1038/s41420-022-01139-6)
Supplement: Supplementary file 18 — Language proofs [file 41420_2022_1139_MOESM18_ESM.pdf]

SPRINGER NATURE

Author Services

Editing Certificate

This document certifies that the manuscript

Human-specific gene CT47 blocks PRMT5 degradation to lead to meiosis arrest

prepared by the authors

Chao Li1,9, Zhengyun Huang1,9, Yuming Feng2,9, Junjie Deng1,8, Yue Gu1, Hanben Wang3, Xin Wu3, Yichen Zhu1, Zhiwei Liu1, Moli Huang1, Tao Wang1, Zhenxin Fu1, Shijun Hu4, Bing Yao2, Chengji J. Zhou5, Steve D M Brown6, Yi Liu7, Antonio Vidal-Puig8, Yingying Dong1*, Ying Xu1,10*

was edited for proper English language, grammar, punctuation, spelling, and overall style by one or more of the highly qualified native English speaking editors at SNAS.

This certificate was issued on **January 24, 2022** and may be verified on the SNAS website using the verification code **E0B4-0B56-708D-A7FB-36C8** .

Neither the research content nor the authors' intentions were altered in any way during the editing process. Documents receiving this certification should be English-ready for publication; however, the author has the ability to accept or reject our suggestions and changes. To verify the final SNAS edited version, please visit our verification page at secure.authorservices.springernature.com/certificate/verify.
If you have any questions or concerns about this edited document, please contact SNAS at support@as.springernature.com.

SNAS provides a range of editing, translation, and manuscript services for researchers and publishers around the world.
For more information about our company, services, and partner discounts, please visit authorservices.springernature.com.
