# Supplementary material for: Human-specific gene CT47 blocks PRMT5 degradation to lead to meiosis arrest
Source: Cell Death Discov. 2022 Aug 2;8:345. doi: 10.1038/s41420-022-01139-6 (PMC9345867; doi:10.1038/s41420-022-01139-6)
Supplement: Supplementary file 19 — animal protocal [file 41420_2022_1139_MOESM19_ESM.pdf]

ANIMAL CARE AND USE PROTOCOL (AP) OF CAMBRIDGE-SUDA GENOMIC RESOURCE CENTER (CAM-SU GRC), SOOCHOW UNIVERSITY

CAM-SU-AP#: YX-2017-3

1. Administrative data.

"Other investigators" refers to all personnel involved in the experiment.

If you don't belong to CAM-SU GRC, you should cooperate with a PI of CAM-SU GRC who should take responsibility for your experiment. And, "co-principal investigator" refers to your own tutor.

**Principal investigator** （PI in CAM-SU）	**Ying Xu**		
**E-mail**	**yingxu@suda.edu.cn**		
**Office phone**	**051265883781**	**Cell phone**	**13390893405**
**Co-principal investigator**			
**E-mail**			
**Office phone**		**Cell phone**	
**Other investigators**	**Chao Li**	**Yingying Dong**	
**E-mail**	**472609914@qq.com**	**yydong@suda.edu.cn**	
**Office phone**			
**Cell phone**	**18862112094**	**17706211615**	
**Project title**	**Study the function of disease-related human new genes**		
**Funding origin (No.)**			
**Estimated start date**	2018.1.1	**Estimated end date**	2020.12.31

**Please check all that apply**	√	New	√	3 year rewrite	√	Transgenic creation
	√	Breeding/maintenance	√	Experimental		Knockout creation
	√	Survival surgery	√	Non-survival surgery	√	Behavior studies
	√	BrdU labeling	√	embryo collection	√	Source of tissues
		Immunization		Monoclonal antibody production		Polyclonal antibody production
	√	Anesthetize and release (blood collection)		Tumor induction or implantation		Other, please specify below:

2. Animal requirements.

(If more than one strain is required, please add the table below for each strain)

Strain/line	C57/BL6J
Gender	M & F

Age range	neonatal day to 2 years old	
weight range	2-50g	
Other requirement		
	Number of animals to be used	Number of cages to be used
Year 1	30	6
Year 2	30	6
Year 3	30	6
Total	90	18

Strain/line	*CT47-BAC*	
Gender	M & F	
Age range	neonatal to 2 years old	
weight range	5-50g	
Other requirement		
	Number of animals to be used	Number of cages to be used
Year 1	100	20
Year 2	100	20
Year 3	100	20
Total	300	60

3. Objective/Hypothesis

Briefly describe in non-technical terms the scientific aims of this project. This is where you will describe the `what' and `why' of your protocol.

Justify the project in terms of its potential value in advancing scientific knowledge and/or the benefits of the study to human and/or animal health. Provide sufficient information to indicate that the potential new knowledge from the project justifies the use of animals, improvement of animal management or production.

Jargon should be avoided or explicitly explained (please define all acronyms).

The origin of new genes with novel functions creates genetic and phenotypic diversity in organisms. Recent studies have identified lineage-specific and species-specific new genes with important phenotypic effects on diverse phenotypes, including development, sexual reproduction, brain functions and behavior. We explore the function of human new genes using bacterial artificial chromosome(BAC) transgenic mice.

4. Rationale for animal use.

Please list the alternative to animal use and potential harmful procedures, such as less-invasive procedures, other species, isolated organ preparation, cell or tissue culture, or computer simulation.

In vitro cell and tissue culture would be used instead of live animals where it is appropriate to do so. In order to explore the human new gene functions, especially their functions in nervous and reproduction system, we have to use transgenic mice to study the physiological

function and assessment their reproductive capacity. Tissues collection from mice of different ages for further analysis.6-8 week old male mice are used for behavioral experiment.

5. Justify the appropriateness of the species/strain selected.

Please indicate what the advantages of the species/strain you choose are, Such as, easy to model, or particular genetic background, or proven susceptibility to particular induction, or expression of particular gene, etc.

CT47-BAC mice are transgenic mice. Because this genes is a human specific new genes that mice do not have. Transgenic technology is a proven technique based on pronucleus injection technology. As for whether these new genes have an effect on mice, there have no literature support. But these new genes all express in testis in human.

✧ **6. Statistical analysis.** (The asterisk indicates this is the focus of review. The same below)

Insufficient justification of animal numbers will result in protocol rejection. Include the total numbers of animals used in each experiment and over a 3-year period. Identify any statistical analysis used to demonstrate why this number of animals is necessary for this study.

We have 3 strains of each transgenic mice. All these genes are functional in nervous system and reproduction. So we plan research nervous and reproduction related phenotype in these mice. 6 pairs breeding mice are needed in each line. We use 14 mice(7 transgenic mice and 7 wildtype) for Open Field Assay, Water Maze Test, EMP Test, Eight-arm radial maze task and Y maze test, total need 70 mice for one round experiment. For reproduction experiment we plan collect 5 pairs tissue(5 transgenic mice and 5 wildtype mice) for each line.

7. Hazardous agents.

Check if hazardous chemicals, toxins, biologicals and radioactive agents are to be used

(Hazardous agents include, but are not limited to: infectious agents including bacterial, chlamydiae, fungi, rickettsias, viruses, parasites, prions, human blood, body fluids, tissues or cell cultures, recombinant DNA and the creation (but not acquisition) of transgenic animals, mutagenic or teratogenic substances; sterilant or anesthetic gasses. Radioactive agents include: x-rays, lasers, sealed sources and radioisotopes.)

If yes, please attach a separate sop on handling substances, animals and equipment.

	**Yes**	√	**No**

**Hazardous agents Category**		Biological/infectious agents		Recombinant DNA
		Hazardous chemicals		Radioisotopes
		Select agents		
		Name of agent(s)		

Where will procedures be performed?

In the conventional side of animal facility

Where will animals be housed?

In the conventional side of animal facility

8. Will you use CAM-SU GRC SOPs?

Check and attach CAM-SU GRC SOPs (\\172.21.1.188\Share\03 CAM-SU Animal Protocol)

List the Number and title of CAM-SU GRC SOPs

AP1 Strain breeding and to maintain
AP18 Euthanasia of Mice (Cervical Dislocation)
AP19 Carcass dispose
AP11 Open field test

✧ 9. Description of experimental design and animal procedures.

Describe the experimental design as it relates to the number of animals indicated in **No. 2. Animal requirements**. Specify animal procedures including inoculations (sites, substances, dosages and schedules), blood withdrawals (volume, frequency and withdrawal sites), surgical procedures (provide details on separate form), radiation (dosage and schedule), tail biopsies. Euthanasia criteria (tumor size, percentage body weight gain or loss, inability to drink, clinical symptomatology or signs of toxicity) must be specified when administration of tumor cells, biologicals, infectious agents, radiation of toxic chemicals are expected to cause significant symptomatology or are potentially lethal. Use of death as an endpoint must be scientifically justified.

1. Marking of mice by toe clipping and tail biopsies for genotyping within 7 days.

2. Mice behavior experiment will in strict accordance with standard SOP of different experiment.

3. Sacrifice of mice by CO2 plus cervical dislocation.

4. All tissue, biochemical, gene expression profiles and cell line studies will be done immediately after cervical dislocation since it does not allow time delay. All samples are collected within 2 min.

10. What is the expected duration of survival after expression of the phenotype?

About 1.5 to 2.5 year.

✧ 11. Pain or Distress Category.

A generally acceptable method of determining whether or not a procedure would be painful is to consider whether it is considered a painful procedure in man. If it is, then appropriate anesthesia or analgesia should be used. CAM-SU GRC currently employs three Pain and Distress Categories C, D, and E (corresponding to the USDA reportable pain categories). Please indicate the type of pain to be experienced with this research.

√	PAIN CATEGORY C		PAIN CATEGORY D		PAIN CATEGORY E

For E, (must be scientifically justified) please cite references below:

Definitions:

Category C: Includes only procedures that are considered to produce minimal, transient, or no pain or distress in animals when performed by a competent individual. The definition of USDA category C also emphasizes that protocols involve no more than momentary or slight pain or distress and no use of pain-relieving drugs. Examples include: breeding protocols, injections of material in amounts that will not cause adverse reactions by the following routes: IV, SC, IM, IP; gavages, restraint, tail cuts.

Category D: Includes procedures that have the potential to produce pain or distress in animals, but which are performed using appropriate and adequate anesthetics, analgesics, or tranquilizers to alleviate the pain or distress. Examples include: retro-orbital bleeds, cannulation or catherization of blood vessels or body cavities under anesthesia, surgical procedures under anesthesia such as biopsies, hepatectomies, stroke, spinal injuries with post-op analgesia.

Category E: Includes potentially painful or distressing procedures that are performed without appropriate and adequate anesthesia, analgesia, or tranquilizers; or are not followed with appropriate measures to alleviate pain or distress; or are not amenable to relief by therapeutic measures. Provide written justification of your requirements. E protocols require the prior review and approval of the full IACUC members before they are initiated. Examples: death as an endpoint studies.

✧ 12. Release of pain or distress.

Will the animals experience pain or distress in association with the phenotype expressed or proposed procedures? What are your plans to avoid or alleviate pain? If your animal protocol involves major survival surgery procedure, please state the pain-releasing drug (component, dose, administration method and time-interval) used post-surgery.

13. Method of health treatment and euthanasia

√	Cervical dislocation*			Exsanguinations with anesthesia			Other***
	Decapitation*			Perfusion under anesthesia			
	Anesthesia overdose**		√	**CO_2 (Recommended)**			

***Cervical dislocation and decapitation are not recommended and if performed must be justified by scientific necessity. Please detail it.**

The separation occurs at the base of the brain or within the cervical spine area (the upper third of the neck). According to the Canadian Council on Animal Care (CCAC), cervical dislocation is normally only conducted on small animals

****Specify agent, dose, frequency and administration route:**

***Specify below:

Please list the health treatment and possible euthanasia in case of the possible animal sickness and failure of protocols.

Retain Carcass for subsequent experiments?

	Yes, 4℃		Yes, -20℃	√	No

14. Training.

All research personnel must be appropriately qualified to perform their work with animals.

Qualifications should be in the following areas:

*the basic biology of each species of animals used.

*proper handling of species used.

*adequate familiarity with experimental protocol and techniques as well as pre- and post-procedural care including aseptic techniques.

Training Certifications:

Researchers, including facility staff, have the knowledge and skills enumerated above.

Trained animal technicians will perform all breeding and/or experimental procedures.

List all the person training record of CAM-SU GRC/other facility on this ANIMAL PROTOCOL:

All of us have been trained by CAM-SU GRC 2016-2017 and hold LAUCC (laboratory animal use and care certificate of Jiangsu province)

15. IACUC notification:

	Request for immediate subcommittee review and action*
	For report at regular IACUC meeting

*Specify below:

16. Principal Investigator Assurance & Signatures:

Principal Investigator Assurance:

To the best of my knowledge, the information contained in the protocol application matching the Protocol Title listed above is accurate.

I affirm that all procedures involving animals will be carried out humanely and will be performed by IACUC certified persons, and that as the designated Principal Investigator, I am responsible for all work conducted under this protocol.

I affirm that the protocol noted above accurately reflects procedural information contained in the

grant application to the agency noted on this form.

I understand that national regulations authorize the attending veterinarian to utilize his/her discretion in the implementation of the procedures herein described in order to assure the welfare of the animal subjects. I further understand that any other variance from what is written in the protocol form would constitute a violation of Animal Welfare guidelines. Any revisions to animal care and use procedures in this project will be forwarded promptly to the IACUC for review. Revisions to protocols will not be implemented until IACUC clearance has been obtained. Animals will not be transferred between investigators without prior written approval.

PI' print name: YING XU

Signature: [illegible] **Date:** 2017/12/1

This Page For IACUC of CAM-SU Administration Use Only

Date Received:__________ Received by:__________

Pre-Reviewed by Veterinarian: Man Gao Signature: [signature] Date: 2017.12.3

Date of IACUC meeting:__________

Result of IACUC review:__________

Comments and Responses:

（Please respond to the comments one by one）

Animal Program Manager: Weiqi He

Signature: [signature] **Date:** 2017-12-10

ANIMAL CARE AND USE PROTOCOL (AP) OF CAMBRIDGE-SUDA GENOMIC RESOURCE CENTER (CAM-SU GRC), SOOCHOW UNIVERSITY

CAM-SU-AP#: YX-2021-1

1. Administrative data.

"Other investigators" refers to all personnel involved in the experiment.

If you don't belong to CAM-SU GRC, you should cooperate with a PI of CAM-SU GRC who should take responsibility for your experiment. And, "co-principal investigator" refers to your own tutor.

**Principal investigator （PI in CAM-SU）**	**Ying Xu**		
**E-mail**	**yingxu@suda.edu.cn**		
**Office phone**	**051265883781**	**Cell phone**	**13390893405**
**Co-principal investigator**			
**E-mail**			
**Office phone**		**Cell phone**	
**Other investigators**	**Chao Li**	**Yingying Dong**	
**E-mail**	**472609914@qq.com**	**yydong@suda.edu.cn**	
**Office phone**			
**Cell phone**	**18862112094**	**17706211615**	
**Project title**	**Study the function of disease-related human new genes**		
**Funding origin (No.)**			
**Estimated start date**	2021-1-1	**Estimated end date**	2023-12-31

**Please check all that apply**	√	New	√	3 year rewrite	√	Transgenic creation
	√	Breeding/maintenance	√	Experimental		Knockout creation
	√	Survival surgery	√	Non-survival surgery	√	Behavior studies
	√	BrdU labeling	√	embryo collection	√	Source of tissues
		Immunization		Monoclonal antibody production		Polyclonal antibody production
	√	Anesthetize and release (blood collection)		Tumor induction or implantation		Other, please specify below:

2. Animal requirements.

(If more than one strain is required, please add the table below for each strain)

Strain/line	C57/BL6J
Gender	M & F

Age range	neonatal day to 2 years old	
weight range	2-50g	
Other requirement		
	Number of animals to be used	Number of cages to be used
Year 1	30	6
Year 2	30	6
Year 3	30	6
Total	90	18

Strain/line	*CT47-BAC*	
Gender	M & F	
Age range	neonatal to 2 years old	
weight range	5-50g	
Other requirement		
	Number of animals to be used	Number of cages to be used
Year 1	100	20
Year 2	100	20
Year 3	100	20
Total	300	60

3. Objective/Hypothesis

Briefly describe in non-technical terms the scientific aims of this project. This is where you will describe the `what' and `why' of your protocol.

Justify the project in terms of its potential value in advancing scientific knowledge and/or the benefits of the study to human and/or animal health. Provide sufficient information to indicate that the potential new knowledge from the project justifies the use of animals, improvement of animal management or production.

Jargon should be avoided or explicitly explained (please define all acronyms).

The origin of new genes with novel functions creates genetic and phenotypic diversity in organisms. Recent studies have identified lineage-specific and species-specific new genes with important phenotypic effects on diverse phenotypes, including development, sexual reproduction, brain functions and behavior. We explore the function of human new genes using bacterial artificial chromosome(BAC) transgenic mice.

4. Rationale for animal use.

Please list the alternative to animal use and potential harmful procedures, such as less-invasive procedures, other species, isolated organ preparation, cell or tissue culture, or computer simulation.

In vitro cell and tissue culture would be used instead of live animals where it is appropriate to do so. In order to explore the human new gene functions, especially their functions in nervous and reproduction system, we have to use transgenic mice to study the physiological

function and assessment their reproductive capacity. Tissues collection from mice of different ages for further analysis.6-8 week old male mice are used for behavioral experiment.

5. Justify the appropriateness of the species/strain selected.

Please indicate what the advantages of the species/strain you choose are, Such as, easy to model, or particular genetic background, or proven susceptibility to particular induction, or expression of particular gene, etc.

humanized CT47 bacterial artificial chromosomes (BACs) transgenic mouse lines (CT47 mice) through microinjection of pbelobac11-ctd-2010p8 (200kb) that carries the cis-acting genomic regulatory elements into the pronucleus of the one-cell mouse embryo. Because this genes is a human specific new genes that mice do not have. Transgenic technology is a proven technique based on pronucleus injection technology. This transgenic mouse can most realistically mimic CT47's function in human. As for whether these new genes have an effect on mice, there have no literature support. But these new genes all express in testis in human.

✧ 6. Statistical analysis. (The asterisk indicates this is the focus of review. The same below)

Insufficient justification of animal numbers will result in protocol rejection. Include the total numbers of animals used in each experiment and over a 3-year period. Identify any statistical analysis used to demonstrate why this number of animals is necessary for this study.

We have 3 strains of CT47 transgenic mice. All these genes are functional in nervous system and reproduction. So we plan research nervous and reproduction related phenotype in these mice. 6 pairs breeding mice are needed in each line. We use 14 mice(7 transgenic mice and 7 wildtype) for Open Field Assay, Water Maze Test, EMP Test, Eight-arm radial maze task and Y maze test, total need 70 mice for one round experiment. For reproduction experiment we plan collect 5 pairs tissue(5 transgenic mice and 5 wildtype mice) for each line.

7. Hazardous agents.

Check if hazardous chemicals, toxins, biologicals and radioactive agents are to be used

(Hazardous agents include, but are not limited to: infectious agents including bacterial, chlamydiae, fungi, rickettsias, viruses, parasites, prions, human blood, body fluids, tissues or cell cultures, recombinant DNA and the creation (but not acquisition) of transgenic animals, mutagenic or teratogenic substances; sterilant or anesthetic gasses. Radioactive agents include: x-rays, lasers, sealed sources and radioisotopes.)

If yes, please attach a separate sop on handling substances, animals and equipment.

	**Yes**	√	**No**

**Hazardous agents Category**		Biological/infectious agents		Recombinant DNA
		Hazardous chemicals		Radioisotopes
		Select agents		
		Name of agent(s)		

Where will procedures be performed?

In the conventional side of animal facility

Where will animals be housed?

In the conventional side of animal facility

8. Will you use CAM-SU GRC SOPs?

Check and attach CAM-SU GRC SOPs (\\172.21.1.188\Share\03 CAM-SU Animal Protocol)

List the Number and title of CAM-SU GRC SOPs

AP1 Strain breeding and to maintain<br>AP18 Euthanasia of Mice (Cervical Dislocation)<br>AP19 Carcass dispose<br>AP11 Open field test

✧ 9. Description of experimental design and animal procedures.

Describe the experimental design as it relates to the number of animals indicated in **No. 2. Animal requirements**. Specify animal procedures including inoculations (sites, substances, dosages and schedules), blood withdrawals (volume, frequency and withdrawal sites), surgical procedures (provide details on separate form), radiation (dosage and schedule), tail biopsies. Euthanasia criteria (tumor size, percentage body weight gain or loss, inability to drink, clinical symptomatology or signs of toxicity) must be specified when administration of tumor cells, biologicals, infectious agents, radiation of toxic chemicals are expected to cause significant symptomatology or are potentially lethal. Use of death as an endpoint must be scientifically justified.

**1. Marking of mice by toe clipping and tail biopsies for genotyping within 7 days.**<br><br>**2. Mice behavior experiment will in strict accordance with standard SOP of different experiment.**<br><br>**3. Sacrifice of mice by CO2 plus cervical dislocation.**<br><br>**4. All tissue, biochemical, gene expression profiles and cell line studies will be done immediately after cervical dislocation since it does not allow time delay. All samples are collected within 2 min.**<br><br>**5. The mice were injected subcutaneously at a concentration of 10 μl (0.5 mg/ml Testosterone propionate solution)/g (Mouse body weight).**

10. What is the expected duration of survival after expression of the phenotype?

About 1.5 to 2.5 year.

✧ 11. Pain or Distress Category.

A generally acceptable method of determining whether or not a procedure would be painful is to consider whether it is considered a painful procedure in man. If it is, then appropriate anesthesia or analgesia should be used. CAM-SU GRC currently employs three Pain and Distress Categories C, D,

and E (corresponding to the USDA reportable pain categories). Please indicate the type of pain to be experienced with this research.

√	PAIN CATEGORY C		PAIN CATEGORY D		PAIN CATEGORY E

For E, (must be scientifically justified) please cite references below:

Definitions:

Category C: Includes only procedures that are considered to produce minimal, transient, or no pain or distress in animals when performed by a competent individual. The definition of USDA category C also emphasizes that protocols involve no more than momentary or slight pain or distress and no use of pain-relieving drugs. Examples include: breeding protocols, injections of material in amounts that will not cause adverse reactions by the following routes: IV, SC, IM, IP; gavages, restraint, tail cuts.

Category D: Includes procedures that have the potential to produce pain or distress in animals, but which are performed using appropriate and adequate anesthetics, analgesics, or tranquilizers to alleviate the pain or distress. Examples include: retro-orbital bleeds, cannulation or catherization of blood vessels or body cavities under anesthesia, surgical procedures under anesthesia such as biopsies, hepatectomies, stroke, spinal injuries with post-op analgesia.

Category E: Includes potentially painful or distressing procedures that are performed without appropriate and adequate anesthesia, analgesia, or tranquilizers; or are not followed with appropriate measures to alleviate pain or distress; or are not amenable to relief by therapeutic measures. Provide written justification of your requirements. E protocols require the prior review and approval of the full IACUC members before they are initiated. Examples: death as an endpoint studies.

✧ 12. Release of pain or distress.

Will the animals experience pain or distress in association with the phenotype expressed or proposed procedures? What are your plans to avoid or alleviate pain? If your animal protocol involves major survival surgery procedure, please state the pain-releasing drug (component, dose, administration method and time-interval) used post-surgery.

13. Method of health treatment and euthanasia

√	Cervical dislocation*		Exsanguinations with anesthesia		Other***
	Decapitation*		Perfusion under anesthesia		
	Anesthesia overdose**	√	**CO_2 (Recommended)**		

***Cervical dislocation and decapitation are not recommended and if performed must be justified by scientific necessity. Please detail it.**

The separation occurs at the base of the brain or within the cervical spine area (the upper third of the neck). According to the Canadian Council on Animal Care (CCAC), cervical dislocation is

normally only conducted on small animals

****Specify agent, dose, frequency and administration route:**

*****Specify below:**

Please list the health treatment and possible euthanasia in case of the possible animal sickness and failure of protocols.

Retain Carcass for subsequent experiments?

	Yes, 4℃		Yes, -20℃	√	No

14. Training.

All research personnel must be appropriately qualified to perform their work with animals.
Qualifications should be in the following areas:
*the basic biology of each species of animals used.
*proper handling of species used.
*adequate familiarity with experimental protocol and techniques as well as pre- and post-procedural care including aseptic techniques.

Training Certifications:
Researchers, including facility staff, have the knowledge and skills enumerated above.
Trained animal technicians will perform all breeding and/or experimental procedures.
List all the person training record of CAM-SU GRC/other facility on this ANIMAL PROTOCOL:

All of us have been trained by CAM-SU GRC 2016-2017 and hold LAUCC (laboratory animal use and care certificate of Jiangsu province)

15. IACUC notification:

	Request for immediate subcommittee review and action*
	For report at regular IACUC meeting

*Specify below:

16. Principal Investigator Assurance & Signatures:

Principal Investigator Assurance:
To the best of my knowledge, the information contained in the protocol application matching the Protocol Title listed above is accurate.
I affirm that all procedures involving animals will be carried out humanely and will be performed by IACUC certified persons, and that as the designated Principal Investigator, I am responsible for all

work conducted under this protocol.

I affirm that the protocol noted above accurately reflects procedural information contained in the grant application to the agency noted on this form.

I understand that national regulations authorize the attending veterinarian to utilize his/her discretion in the implementation of the procedures herein described in order to assure the welfare of the animal subjects. I further understand that any other variance from what is written in the protocol form would constitute a violation of Animal Welfare guidelines. Any revisions to animal care and use procedures in this project will be forwarded promptly to the IACUC for review. Revisions to protocols will not be implemented until IACUC clearance has been obtained. Animals will not be transferred between investigators without prior written approval.

PI' print name:

Signature: **Date: 2020-12-18**

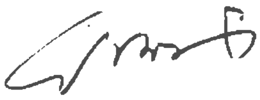

Date Received:________________________Received by:__________________________

Pre-Reviewed by Veterinarian: Man Gao Signature: 高嫚 Date: 2020-12-25

Date of IACUC meeting:____

Result of IACUC review:____

Comments and Responses:

（Please respond to the comments one by one）

Animal Program Manager: Weiqi He

Signature： [illegible] **Date: 2020-12-25**
